# The segmentation and intelligent recognition of structural surfaces in borehole images based on the U^2^-Net network

**DOI:** 10.1371/journal.pone.0299471

**Published:** 2024-03-07

**Authors:** Qingjun Yu, Guannan Wang, Hai Cheng, Wenzhi Guo, Yanbiao Liu

**Affiliations:** 1 Chifengshan Jinhongling Nonferrous Mining Co., Ltd, Inner Mongolia, PR China; 2 Key Laboratory of Ministry of Education on Safe Mining of Deep Metal Mines, Northeastern University, Shenyang, PR China; University of Kragujevac, SERBIA

## Abstract

Structural planes decrease the strength and stability of rock masses, severely affecting their mechanical properties and deformation and failure characteristics. Therefore, investigation and analysis of structural planes are crucial tasks in mining rock mechanics. The drilling camera obtains image information of deep structural planes of rock masses through high-definition camera methods, providing important data sources for the analysis of deep structural planes of rock masses. This paper addresses the problems of high workload, low efficiency, high subjectivity, and poor accuracy brought about by manual processing based on current borehole image analysis and conducts an intelligent segmentation study of borehole image structural planes based on the U^2^-Net network. By collecting data from 20 different borehole images in different lithological regions, a dataset consisting of 1,013 borehole images with structural plane type, lithology, and color was established. Data augmentation methods such as image flipping, color jittering, blurring, and mixup were applied to expand the dataset to 12,421 images, meeting the requirements for deep network training data. Based on the PyTorch deep learning framework, the initial U^2^-Net network weights were set, the learning rate was set to 0.001, the training batch was 4, and the Adam optimizer adaptively adjusted the learning rate during the training process. A dedicated network model for segmenting structural planes was obtained, and the model achieved a maximum F-measure value of 0.749 when the confidence threshold was set to 0.7, with an accuracy rate of up to 0.85 within the range of recall rate greater than 0.5. Overall, the model has high accuracy for segmenting structural planes and very low mean absolute error, indicating good segmentation accuracy and certain generalization of the network. The research method in this paper can serve as a reference for the study of intelligent identification of structural planes in borehole images.

## Introduction

Structural planes are various geological interfaces that exist in rock masses and divide the complete rock mass into blocks [1]. The existence of structural planes reduces the strength and stability of rock masses [2], affecting their mechanical properties and deformation and failure characteristics [3–6]. Therefore, it is an important task in rock mass quality investigation to identify the types, distribution, and scales of structural planes. Currently, digital panoramic borehole imaging technology [7–11] uses optical imaging principles to directly obtain images of the borehole wall and utilizes borehole images to obtain distribution characteristics of the structural planes. As a direct method for observing the inside of a rock mass, it provides technical conditions for accurately investigating structural planes. Currently, the processing of borehole images is mainly completed by naked eye recognition of structural planes and manual sketching of traces. This method is labor-intensive, and the accuracy of identification depends largely on the subjective influence. Therefore, carrying out intelligent recognition of borehole images is of great significance for improving the efficiency and accuracy of borehole image analysis and reducing workload.

Traditional image processing techniques analyze borehole images by segmenting the structural plane areas based on the significant differences in color, brightness, gray gradient, and texture between the structural planes and background rock mass images, and then further analyzing the characteristics of structural planes. For example, in the research by Wang Chuan-ying et al. [12], the vertical depth projection segmentation of structural plane areas was based on the pixel grayscale and gradient mutation characteristics, and the structural plane feature curve was obtained using the standard sine function matching method. Such processing methods are often limited by their low algorithmic generalization and sensitivity to noise, and cannot clearly and completely segment the structural plane areas, requiring secondary processing or manual assistance, which is a semi-automatic processing method.

In recent years, the development of deep learning image processing techniques has promoted the progress of borehole image automatic processing technology. For example, Gao Xu used an unsupervised heterogeneous adaptive algorithm called Deep CORAL [13], which uses a convolutional network to extract borehole image features, defines the CORAL loss function to minimize the difference between source domain and target domain features, and finally outputs pixel points and structural plane classification information using the SoftMax layer. This study demonstrates the feasibility of deep learning for structural plane recognition in borehole images, but the deficiency is that the network uses fully connected layers and cannot handle images of arbitrary pixels. In addition, the recognition accuracy also needs to be improved. Anna Fabijańska et al. [14] realized the layered extraction and thickness automatic measurement of glacier lake sediment core images by constructing a fully convolutional image segmentation network called Deep Varve Net. Glacier lake sediment layering images are fracture-like images with significant color differences between layers. This study demonstrates that multi-layer fully convolutional networks have a certain effect on image segmentation of fracture-like traces.

This paper applies the U^2^-Net [15], a deep semantic segmentation network, to the task of segmenting structural planes in borehole images. U-Net is a deep learning model designed for image segmentation, based on the fully convolutional neural network (FCN) architecture. Its distinctive feature is the U-shaped structure that connects the encoder and decoder, enabling accurate pixel-level segmentation of input images. It is capable of handling images with complex structures and details, exhibiting good performance and scalability. Many improvements have been made to the U-Net architecture. Based on the U-Net architecture, Guo Yan et al. proposed an expanded of U-Net, which can retain the texture details and edge features of tumors, and realize accurate segmentation and multi-class recognition of breast ultrasound images more quickly and automatically [16]. Liu Tao et al. proposed a bridge crack segmentation method based on densely connected U-Net network (BC-DUnet), which can eliminate the influence of complex background on bridge crack segmentation accuracy, achieve higher precision segmentation, additionally, it is resistant to overfitting due to its enhanced generalization capability [17]. Using the MP-Net model derived from U-Net, Park et al. proposed an image segmentation method based on deep learning, which can correctly distinguish fluorescent MP from other elements in a given microscope image, improving the accuracy of predicting MP concentration [18]. Yuan proposes a flexible method CSM-Net for the joint segmentation of IMC and Lumen in carotid ultrasound images, introducing a cascaded dilation convolution and squeezing excitation module to utilize more contextual features at the highest level of the encoder [19]. Wang analyzes digital rock images, which are important for geological research and the flow in the subsurface. It presents a workflow forassessing digital rock petrophysical properties based on machine learning techniques [20].

U^2^-Net, another variation of U-Net, adopts extensive feature fusion to preserve the resolution of output feature maps, and its two-level nested network structure effectively prevents overfitting. Through training and evaluation on a self-constructed borehole image dataset, the network demonstrates precise segmentation results with accurate boundaries and no noise, when dealing with structurally coherent surfaces, the U^2^-Net’s segmentation results require no post-processing, and can be directly used for structural surface feature analysis.

Therefore, this approach achieves accurate segmentation of structural plane regions in borehole images and provides insights for fully automated analysis of such images.

## Segmentation of structural planes in borehole images

### U^2^-Net algorithm

**Fig 1:**
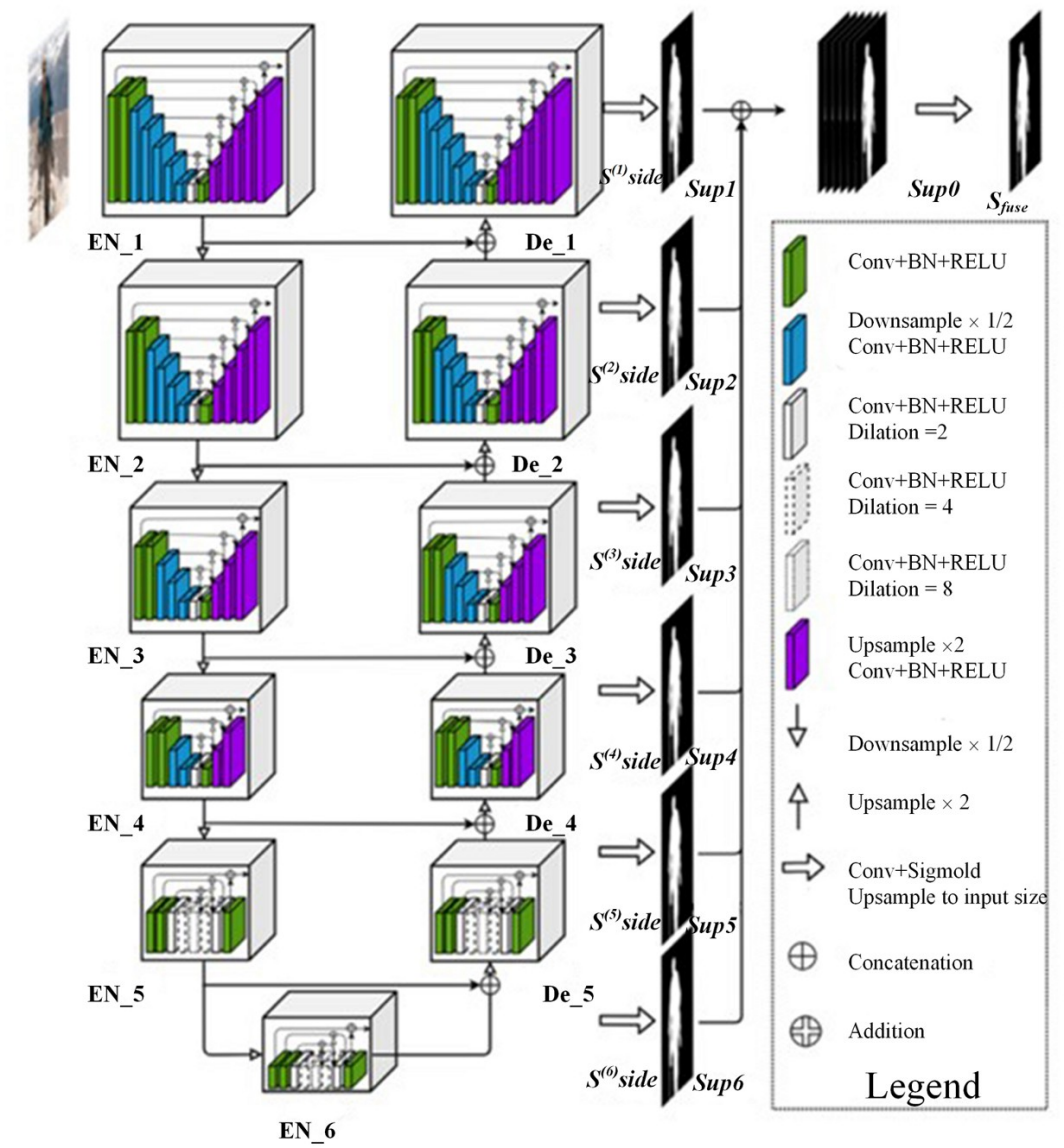
U^2^-Net network structure diagram [8].

U^2^-Net [21–24] is an image semantic segmentation network that combines multi-scale feature extraction and a two-level nested residual network structure. It ensures the output of high-resolution feature maps while controlling memory usage and computational costs at a lower level. Fig 1 shows the overall structure of the network. The U^2^-Net network consists of an encoder and a decoder with a U-shaped structure. Each encoder-decoder is a U-shaped residual block. The network forms 2 layers of nested U-shaped networks in its structure. The network uses 5 times 2x down sampling and up sampling in total, with a total sampling factor of 32x. The feature map down sampling of each layer is fused with the up sampled feature map of the next layer, and the up sampled feature map of each layer is output, denoted as $S_{side}^{\left(i\right)}\in R^{H\times W},\left(i=1,2,\ldots ,6\right)$. The 6 output feature maps are then combined through feature fusion to obtain S_side_ ∈ R^H×W×6^. The S_side_ is then processed by a single-layer convolution operation and the sigmoid activation function, which maps any real number to the (0,1) interval, to output the final predicted feature map S_fuse_ ∈ R^H×W^.

Due to the camera being taken inside the hole, relying on the equipment’s built-in light source for camera shooting; The object is the rock and joints and fractures in the rock wall of the borehole. Due to the different absorption and reflection of light by different lithology rocks and mineral components, the image quality of borehole photography is relatively poor and the resolution is not high. Compared with the U-Net method, it greatly improves the ability to extract multi-scale features from input feature maps of any resolution. So we choose to use U^2^-net for drilling image joint recognition.


$$sigmoid\left(z\right)=\frac{1}{1+e^{-z}}$$ (1)

**Fig 2:**
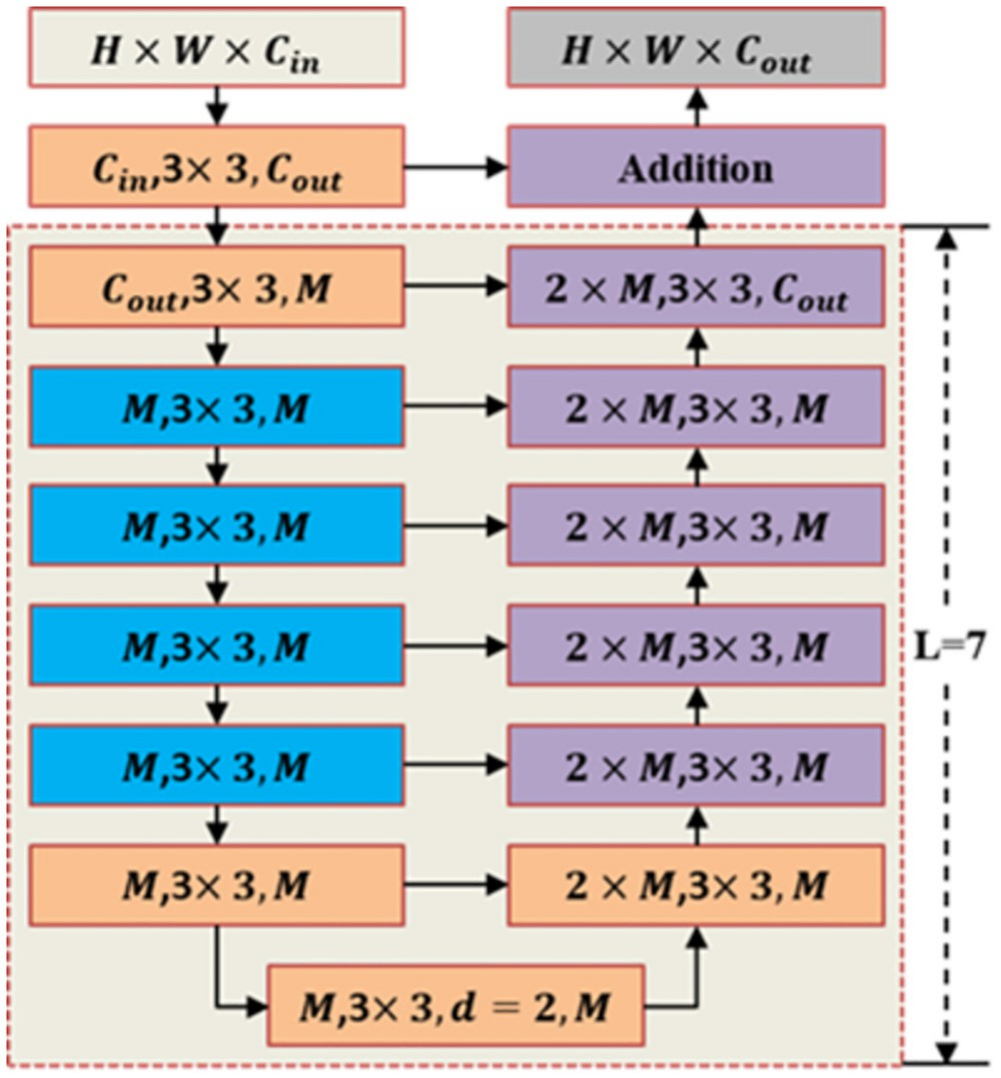
RSU-7 structure diagram [8].

The encoder-decoder structure of each layer in the U^2^-Net network is the same, but the size of the encoder-decoder at different layers is different. The encoder-decoder used in the first layer of the network is a seven-layer U-shaped residual block, as shown in Fig 2. The higher layers of the network use U-shaped residual blocks with fewer layers. From the first to the sixth layer, the number of layers in each U-shaped residual block decreases sequentially. It can be seen from the structure of the U-shaped residual block that the encoder-decoder does not change the shape of the feature map.

### Borehole image segmentation

**Fig 3:**
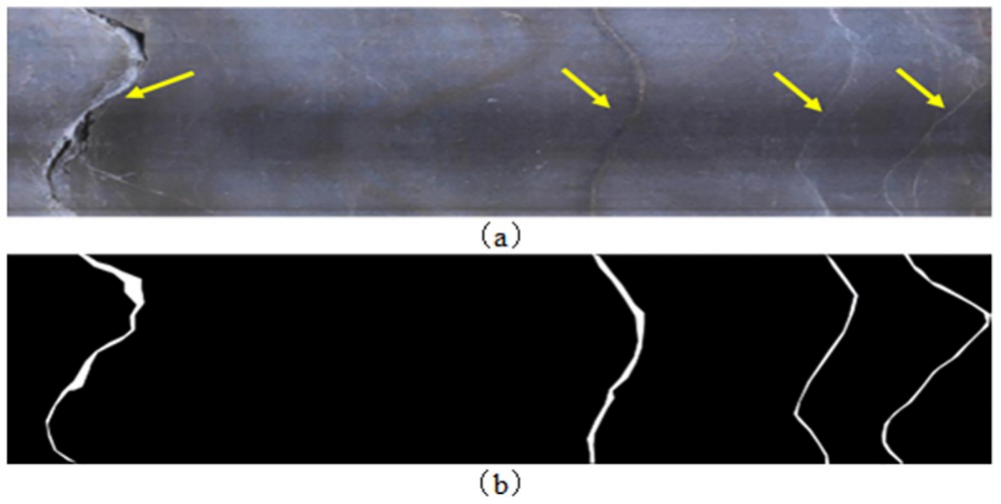
Example of drilling image and structural plane segmentation results: (a) Drill hole image (yellow arrow indicates structural plane); (b) Structural plane segmentation results (white areas represent structural planes).

**Fig 4:**
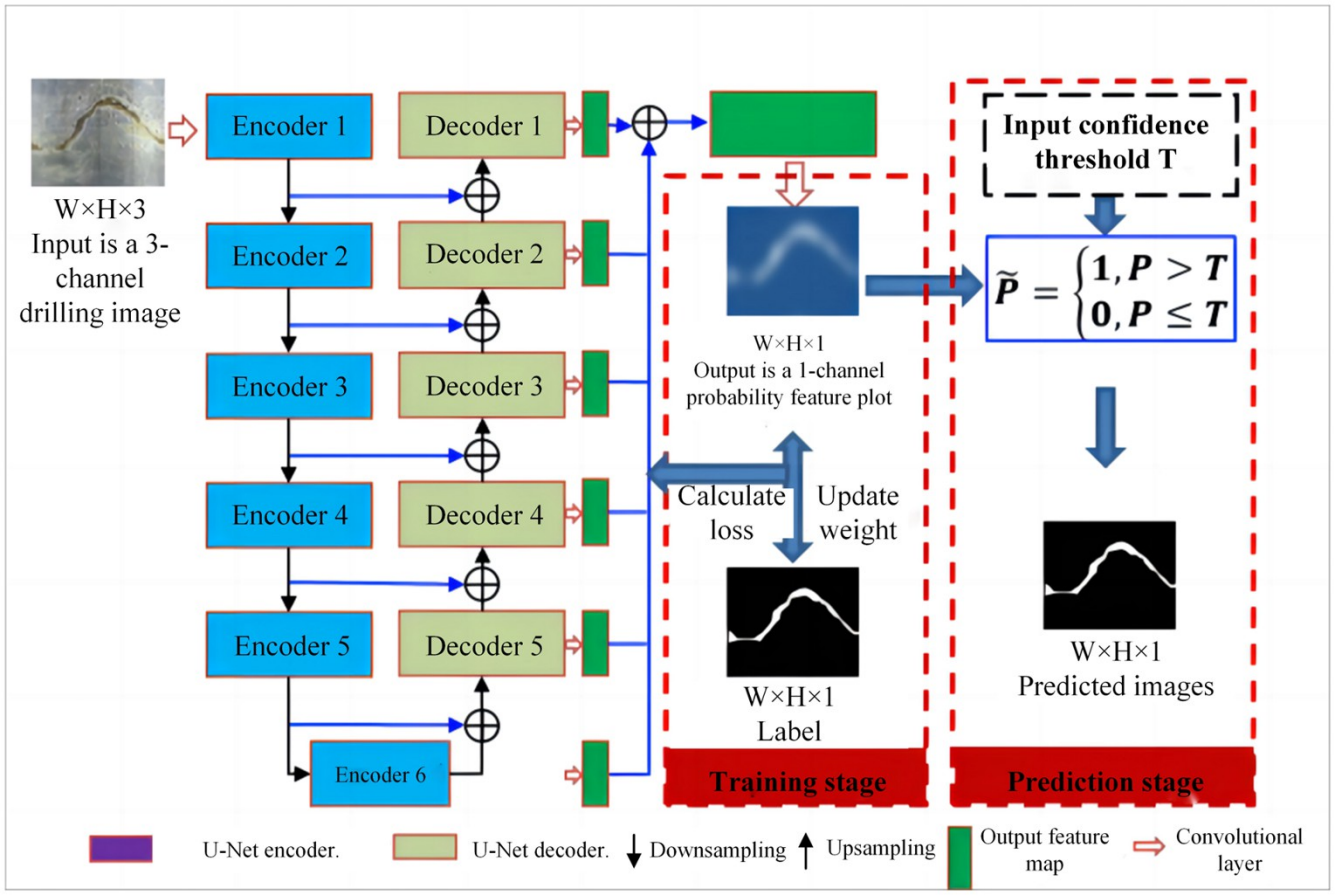
Schematic diagram of structural plane segmentation.

The drilling image is an unfolded image of the inner wall image of a borehole. The morphology of the structure plane on the image is represented by a sine wave curve, and the area of the rock mass that is different from the background in terms of color and texture, as shown in Fig 3(a), is segmented using the U^2^-Net network to form a segmented image with white foreground for the structure plane and black background for the rock mass area, as shown in Fig 3(b). The U^2^-Net network is a fully convolutional network that can process borehole images of any size, and the segmentation goal is a 2-class problem that distinguishes the structural plane foreground from the rock mass background. Therefore, for an RGB borehole image inputting with a size of W×H×3, the network output is a single-channel feature map with a size of W×H×1 after calculation. The value range of the output feature map is (0,1). A confidence threshold T is set ∈(0,1). For each value in the feature map, those greater than T are set to 0 and those less than T are set to 1. The feature map processed by the function C(z) is the final structure plane segmentation map. The selection of threshold T has an impact on the segmentation effect of the structure plane. In this paper, the T value that provides the best evaluation indicator is selected as the threshold. The schematic diagram of structural plane segmentation is shown in Fig 4.


Cz=0,ifz<T1,ifz≥T (2)

### Evaluation indicators for structural plane segmentation

The structural plane segmentation index utilizes image semantic segmentation index values. In the segmentation map, pixel values represent categories, and 0 represents rock background, denoted as negative categories; 1 represents the structural plane class, denoted as a positive class. Therefore, the classification of individual pixels by the network can be divided into the following four situations.

① TP (True Positions): The number of samples that are positively classified.
② FP (False Positions): The number of samples that are judged as positive for negative classes.
③ FN (False Negatives): The number of samples that are judged as positive or negative.
④ TN (True Negatives): The number of samples that are judged as negative.

As a single indicator cannot comprehensively evaluate the segmentation effect, five indicators are used, including precision, recall, PR curve, F-measure, and mean absolute error. Precision and recall are used to observe the training process, draw the PR curve, and calculate the F-measure. The PR curve is used to macroscopically compare the performance of different models, and the F-measure is used to quantitatively compare the superiority or inferiority of the models.

1. Precision (P) is the proportion of true positive pixels that are correctly classified as positive among all pixels that are predicted as positive.
$$P=\frac{TP}{TP+FP}$$ (3)
2. Recall (R) is the proportion of true positive pixels that are correctly classified as positive among all positive pixels.
$$R=\frac{TP}{TP+FN}$$ (4)
3. The PR curve is a curve plotted with recall as the abscissa and precision as the ordinate. For the output results of the same network, a classification threshold is applied to classify the categories. One threshold corresponds to a recall-precision value pair. By adjusting the threshold, a set of recall-precision value pairs is obtained, from which the PR curve is drawn. For different networks, the higher the position of the PR curve, the better the model.
4. The F-measure (Fβ) is a metric used to comprehensively evaluate recall and precision, which is the weighted harmonic mean of recall and precision under non-negative weight β.
$$F_{\beta }=\frac{\left(1+\beta^{2}\right)PR}{\beta^{2}P+R}$$ (5)
  In reference to previous studies on similar image segmentation tasks, this paper believes that precision is more important than recall. As an empirical value for β2, 0.3 is chosen to increase the weight of precision.
$$F_{\beta }=\frac{1.3P\times R}{0.3P+R}$$ (6)
5. The mean Absolute Error (MAE) represents the average difference between the predicted feature map and the label map. Let the position of a pixel in the image be denoted by (r,c), the value of the pixel in the output feature map at the position (r,c) is denoted by P(r,c), the value of the pixel in the label map at the position (r,c) is denoted by G(r,c), the height of the image is H, and the width of the image is W, then the MAE is defined as follows:
$$MAE=\frac{1}{W\times H}\sum_{r=1}^{H}\sum_{c=1}^{W}\left|P\left(r,c\right)-G(r,c)\right|$$ (7)
  The smaller the MAE value, the smaller the difference between the output feature map and the label, indicating a more accurate prediction. The larger the value, the less accurate the prediction.

## Borehole image dataset

**Fig 5:**
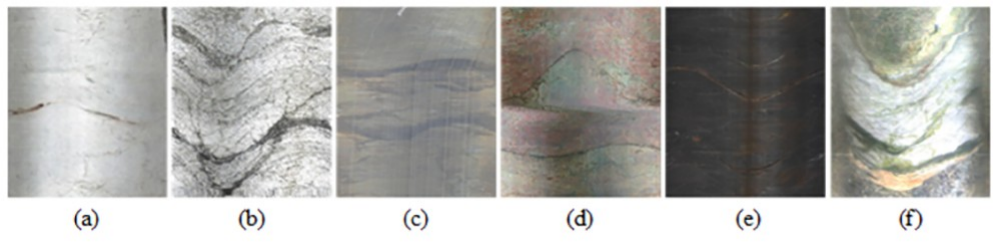
The color distribution of borehole images: (a) White; (b) Grey; (c) Light Red; (d) Light Green; (e) Black Brown; (f) Heather.

A drilling image dataset was constructed to train and evaluate the structure plane extraction network. The drilling images were collected from 20 drilling data in multiple engineering examples, and the rock mass where the drilling was located consisted of six rock types including granite, slate, marble, silica-carbonate rock, quartz sandstone, and dolomite. The drilling images contain white, gray, light red, dark brown, green, and miscellaneous colors(Fig 5). In addition, they also include three types of defective images, i.e., blur, color abnormality, and reflection. The images collected from different engineering projects have different sizes. To facilitate batch training of the network, the drilling images were uniformly cropped to 640 pixels in the length direction and scaled to 360 pixels in the width direction, resulting in 1,013 unmarked drilling images with a size of 640×360.

### Marking structural planes

#### (1) Principles of structure plane annotation

Recognizing structure planes is important for evaluating rock mass quality, with a focus on the types of structure planes that cause damage to the rock mass. However, not all structure planes have a negative impact on rock mass; in fact, some even strengthen it. If too much attention is paid to these types of structure planes during analysis, it may affect the accuracy of rock mass evaluation. Therefore, when annotating structure planes, types of structure planes with little or no impact on the rock mass should be excluded.

According to the formation reason of structure planes, the structure planes can be classified as shown in Table 1. Interface mixing in igneous structure planes and well-developed material interfaces in sedimentary structure planes have a relatively small impact on the rock mass. Therefore, these two types of structure planes are not annotated as structure planes in drilling images and are treated as rock background regions. In addition, this paper does not recognize the dissolution and weathering types of secondary structure planes and does not include images of cave walls in dissolved or weathered rock mass in drilling images.

**Table 1:** Structural plane classification.

Structural plane type			Annotation
Parent Categories	Subcategories	Tertiary Categories	
Primary Structural Planes	Igneous Structural Planes	Fracture Contact	Yes
		Crush Contact	Yes
		Mixture Contact	No
		Primary Joint Surface	Yes
	Sedimentary Structural Planes	Material Interface	No
		Fissure Surface	Yes
		Nonconformity Surface	Yes
		Interbed	Yes
	Metamorphic Structural Planes	Schistosity	Yes
		Slaty Cleavage	Yes
Structural Planes	Fault Zone	Fault Zone	Yes
		Joint Surface	Yes
		Cleavage, etc.	Yes
Secondary Structural Planes	Formed by Excavation	Blast Fracture	Yes
		Stress Relief Fracture	Yes
	Formed Naturally	Unloading Fracture	Yes
		Dissolution Fracture	No
		Weathering Fault	No
		Weathering Interbed	No
		Weathering Fracture	No
		Weathering Vein	No

**Fig 6:**
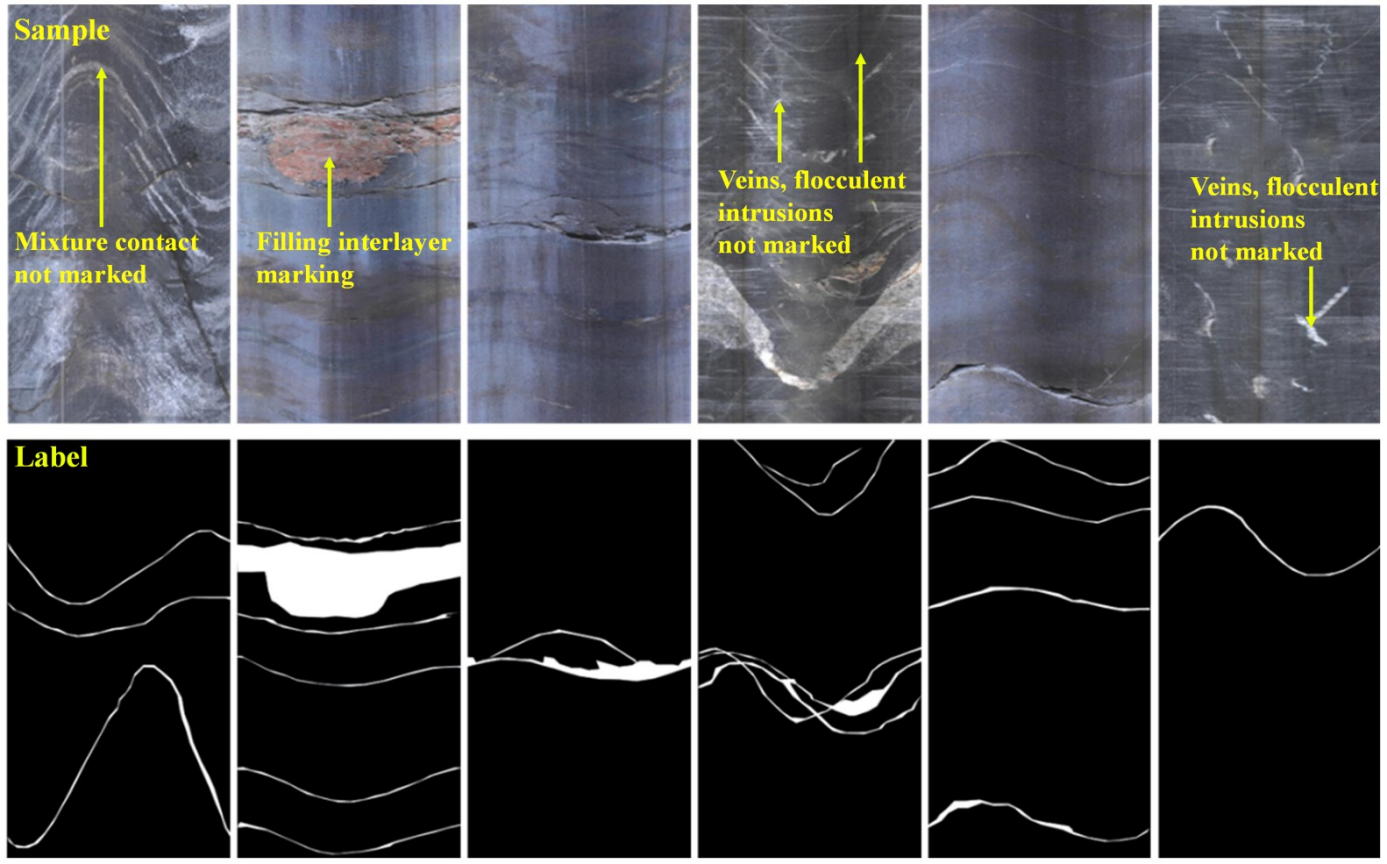
Marking example.

After excluding two types of structure planes, the remaining types of structure planes are not further distinguished and are considered structure plane types. Therefore, the structure plane type does not include material interface and interface mixing types of structure planes and is marked as white on the image. The other type is the rock type in the background, marked in black. The results of structure plane marking are shown in Fig 6.

#### (2) Annotation method

The open-source annotation tool LabelMe was used for image annotation, which is an image annotation software in the field of image recognition. The engineering personnel judged the content of drilling images, identified the structure plane area, and used the polyline tool to outline the structure plane and set the category as the structure plane type. After the annotation was completed, the software output the label image, as shown in Fig 6.

### Dataset partitioning

To train the model, validate the model’s ability, improve the model’s performance, and satisfy the needs of model testing during the experimental process, the dataset was divided into a training set, a validation set, and a testing set. The training set was used to train the deep learning model; the validation set was used to adjust the network parameters and select features; the testing set was used to evaluate the algorithm performance without adjusting the model or parameters. All indicators in this paper were obtained from the testing set. The size of the training set, validation set, and testing set is 60%, 20%, and 20% of the total dataset, respectively. To ensure that the training set, validation set, and testing set samples follow the same distribution, each dataset contains as many types of structure planes as possible. Initially, 607 images (about 60% of the total) were randomly selected and assigned to the training set; 203 images (about 20% of the total) were randomly selected from the remaining images and assigned to the validation set; and the remaining 203 images were assigned to the testing set.

### Data expansion

**Fig 7:**
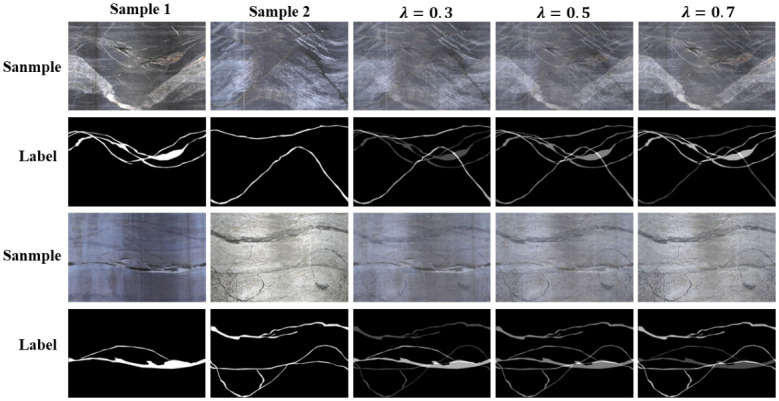
Mixup data expansion.

Data augmentation, also known as data expansion, aims to generate more data from limited data, increase the number and diversity of training samples, add noise data, and improve model robustness. Data augmentation is applied to the training set data in the training stage. With sufficient computing resources, augmented data is generated when loading images during the training process. Due to limited computing resources, this paper pre-augmented the images in the training set before training and unifiedly loaded the original training set images and augmented data during training without distinction, to achieve the purpose of expanding training samples. In addition to commonly used methods such as flipping, color transformation, and blurring, the mixup method is used as a data augmentation method in this study(Fig 7). The mixup method is a way to expand data to twice the original amount. Specifically, two data (x_i_, y_i_) and (x_j_, y_j_) are randomly selected from the samples, and the new sample data $\left(\tilde{x},\tilde{y}\right)$ is obtained from their difference. The data generation method is:

$$\tilde{x}=\lambda x_{i}+(1-\lambda )x_{j}$$ (8)

$$\tilde{y}=\lambda y_{i}+(1-\lambda )y_{j}$$ (9)

Where λ is the interpolation coefficient, with values between 0 and 1. The larger the λ value, the closer the generated data is to (x_i_, y_i_), and the smaller the λ value, the closer it is to (x_i_, y_i_).

The data augmentation increased the amount of training data from 607 images to 12421 images. Specifically, each image was horizontally and vertically flipped to produce 607 more images, while color jitter and mean blur were applied to randomly selected images from the original training set to produce an additional 600 images. The mixup method was used to generate 5000 images with λ = 0.5 and 5000 images with λ = 0.7, as shown in Table 2.

**Table 2:** Training set data expansion.

Original Training Set Images	Horizontal Flipping	Vertical Flipping	Color Jittering	Mean Blurring	Mixup Taking λ values of 0.5, 0.7	Total
607	607	607	300	300	10,000	12,421

## Network training and evaluation

### Network training

The model training used the open-source deep learning framework PyTorch and loaded the original U^2^-Net network weights as a pre-training model to initialize the network weights. The initial learning rate was set to 0.001, batch size was 4, and the Adam optimizer was used as the optimization algorithm, which can adaptively adjust the learning rate during the training process, with an exponential decay rate of β1 = 0.9, β2 = 0.999, and a weight decay coefficient λ = 0.

**Fig 8:**
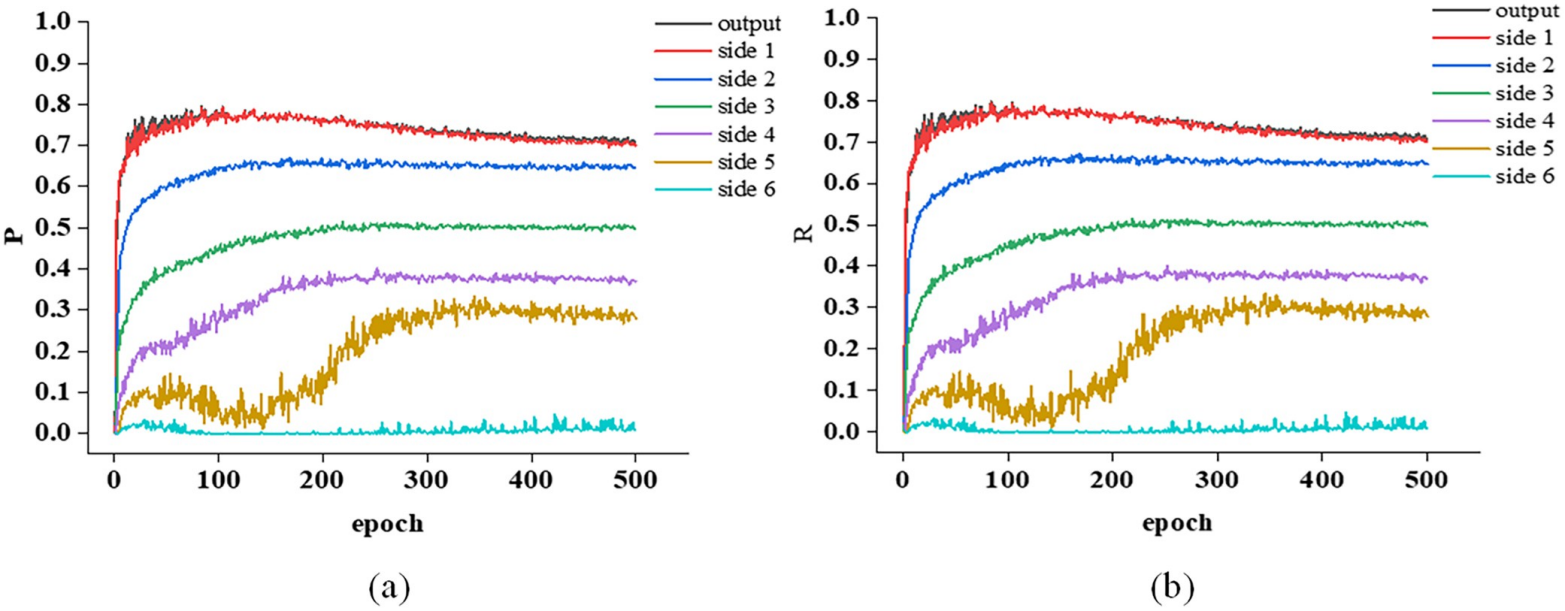
Verification of Indicator Values on the Set: (a) Precision Round Curve; (b) Recall rate round curve.

During the model training process, the precision and recall of the model learned in each round were statistically verified on the validation set, with a confidence threshold of T set to 0.5. In addition to checking the overall output feature map of the model, the output of each layer of the model was also checked. The output of each layer was named from top to bottom as side 1, side 2, …, side 6, and the precision-recall-round curve graph during the training process is shown in Fig 8.

From the metric values on the validation set, the outputs of each layer from layers 1 to 6 of the model, the higher the layer, the lower its precision and recall, and the better the output metric values of the upper layers. The output feature indicators of side 6 are the lowest, and with the increase of training rounds, the indicator values tend to stabilize, indicating that the model has converged.

### Network assessment

**Fig 9:**
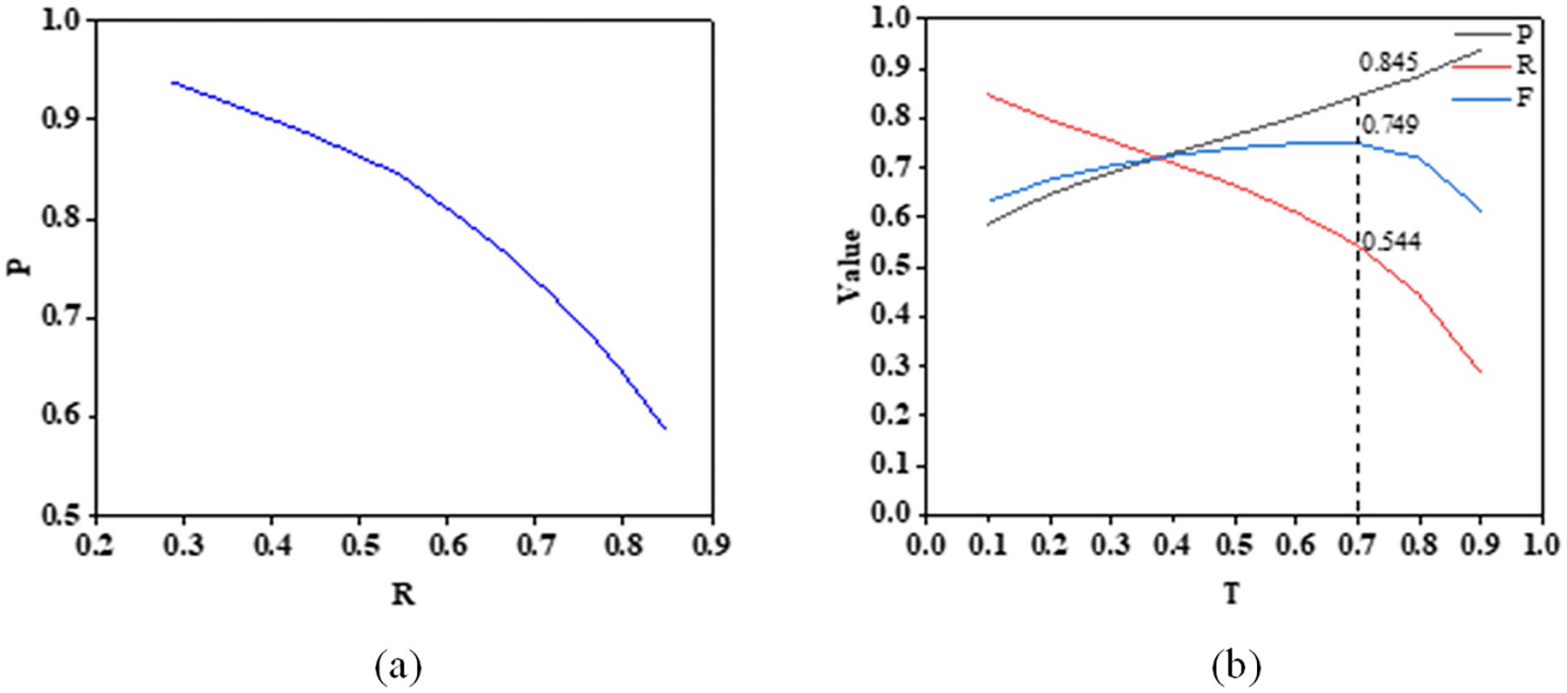
Indicator Values on the Test Set: (a) Confidence Accuracy, Recall, and F-metric Curve; (b) P-R Curve.

By setting different confidence thresholds and testing the model on the test set, precision, recall, and F-measure values can be obtained under different thresholds. The confidence threshold T corresponding to the maximum F-measure value is selected as the confidence threshold used for model prediction. T values were set from 0.1 to 0.9, with an interval of 0.1, resulting in a total of 9 values. The model’s precision, recall, F-measure, and P-R curves were calculated on the test set(Fig 9).

**Fig 10:**
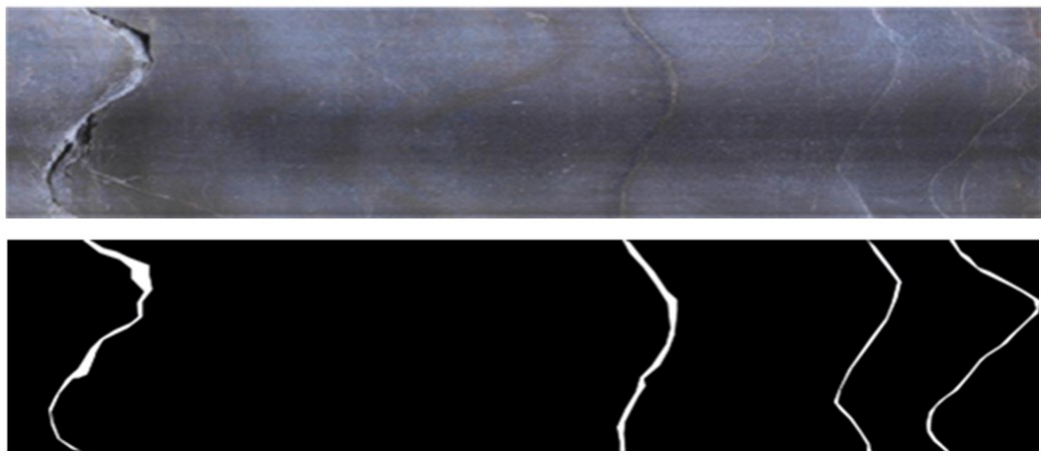
Structural plane recognition effect.

From the evaluation metrics of the model on the test set, it can be seen that the F-measure value reaches the maximum value of 0.749 when the confidence threshold is set to 0.7, and the highest precision can reach 0.845 within the recall range greater than 0.5. Overall, the model has high precision and extremely low average absolute error in segmenting the structural surface, indicating a cleaner segmentation result, which has a strong inhibitory effect on noise data in the image. However, compared with precision, the recall metric is relatively low, resulting in a thinner segmentation of the structural surface area. The main reason is that the edge area of the structural surface contains the most noise, and under high precision, the model eliminates these edge areas during the segmentation process and retains relatively central areas. This result is acceptable because, for the recognition result of the structural surface, this paper focuses more on the position and shape of the central line of the structural surface. The edges of the structural surface in the image are affected by the imaging conditions, and the definition of the edges is relatively vague. It is reasonable and necessary to remove the edge areas appropriately. The identification result is shown in Fig 10.

## Discussion

### Segmentation performance

To verify that the semantic segmentation method of deep learning has better segmentation performance on the structural surface compared to traditional methods, the U^2^-Net method was evaluated on the entire test set of 203 images, along with global threshold segmentation, Otsu method, Sobel operator edge detection, and Canny operator edge detection. To ensure fairness in the comparison between different methods, the metrics of traditional methods were the best results on the test set.

**Fig 11:**
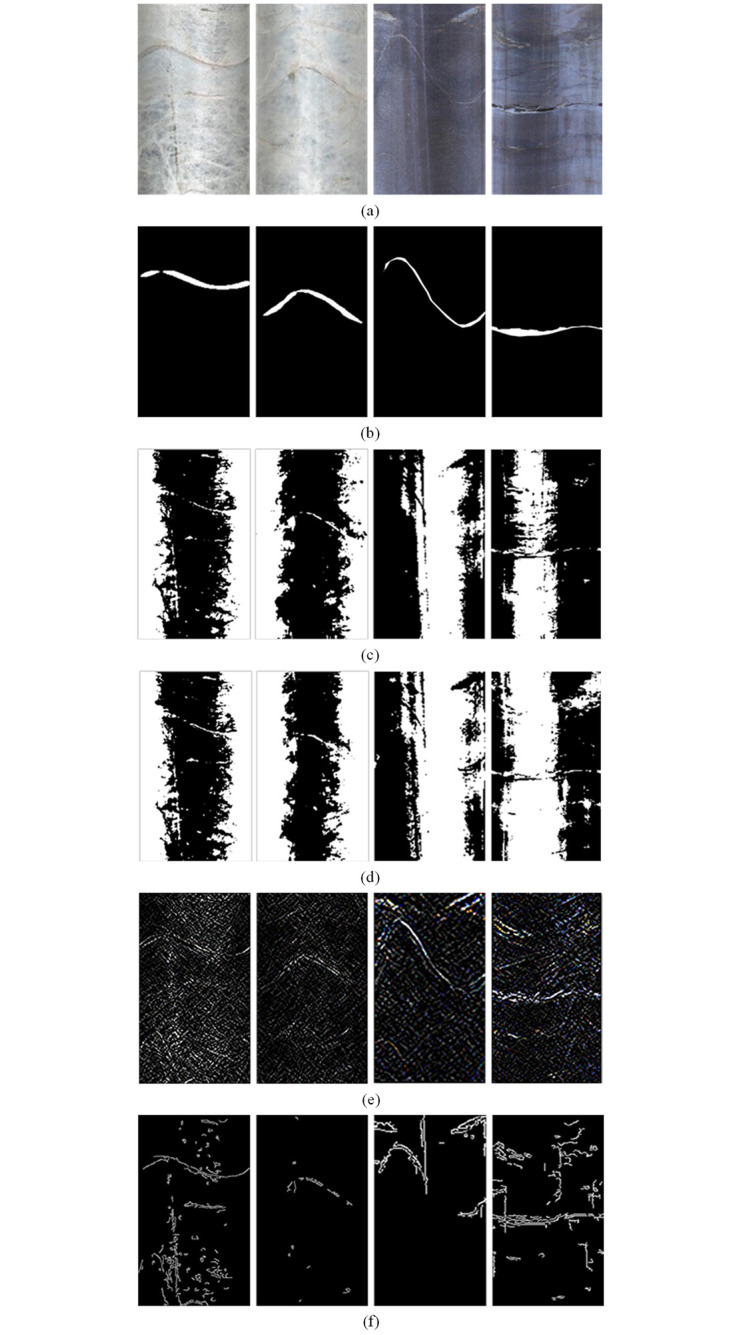
Display of segmentation effects of structural planes using various methods: (a) Original image; (b) U^2^-Net segmentation; (c) Global Maximum Threshold Method for Segmentation; (d) Otsu method segmentation; (e) Sobel operator segmentation; (f) Canny operator segmentation.

For the parameter values of traditional segmentation methods, different parameter values were obtained by enumerating the parameter space, and different parameter values were set on the test set to calculate the corresponding metrics. Using the F-measure value as a benchmark, the parameter value corresponding to the maximum F-measure value was the optimal parameter of each algorithm. With the help of the function interface provided by CV2 (Python version of the OpenCV image processing library), it was found through experiments that the best segmentation threshold for global threshold segmentation was 121; the Otsu method is an adaptive threshold segmentation algorithm without additional parameter settings; Sobel operator segmentation was the highest on the test set when the operator was first-order and the size was 3; Canny operator segmentation was the best when the minimum and maximum thresholds were 80 and 150, respectively. The segmentation metrics of each method on the test set are shown in Table 3, and Fig 11 shows examples of different methods for segmenting the structural surface.

**Table 3:** The indicator values of each method on the test set.

Methods	Precision	Recall	Mean absolute error	F-measure
U^2^-Net	0.8452	0.5438	0.0040	0.7494
global threshold	0.0063	0.5675	0.5618	0.0081
Otsu method	0.0073	0.5579	0.5531	0.0094
Sobel operator	0.0118	0.4982	0.3989	0.0153
Canny operator	0.0072	0.3708	0.3721	0.0093

From the segmentation metrics, the U^2^-Net method has an absolute advantage over the other four traditional methods, including global thresholding, the Otsu method, Sobel operator edge detection, and Canny operator edge detection. Compared to the four traditional methods, the U^2^-Net method has an absolute high precision while maintaining a similar recall rate. In terms of segmentation results, the U^2^-Net method has no noise points and clear and coherent structural surface shapes, while the four traditional methods result in more noise points and unclear structural surface shapes. Overall, the image semantic segmentation method of deep learning has better segmentation performance on the structural surface compared to traditional single segmentation methods.

### Defect image segmentation effect

**Fig 12:**
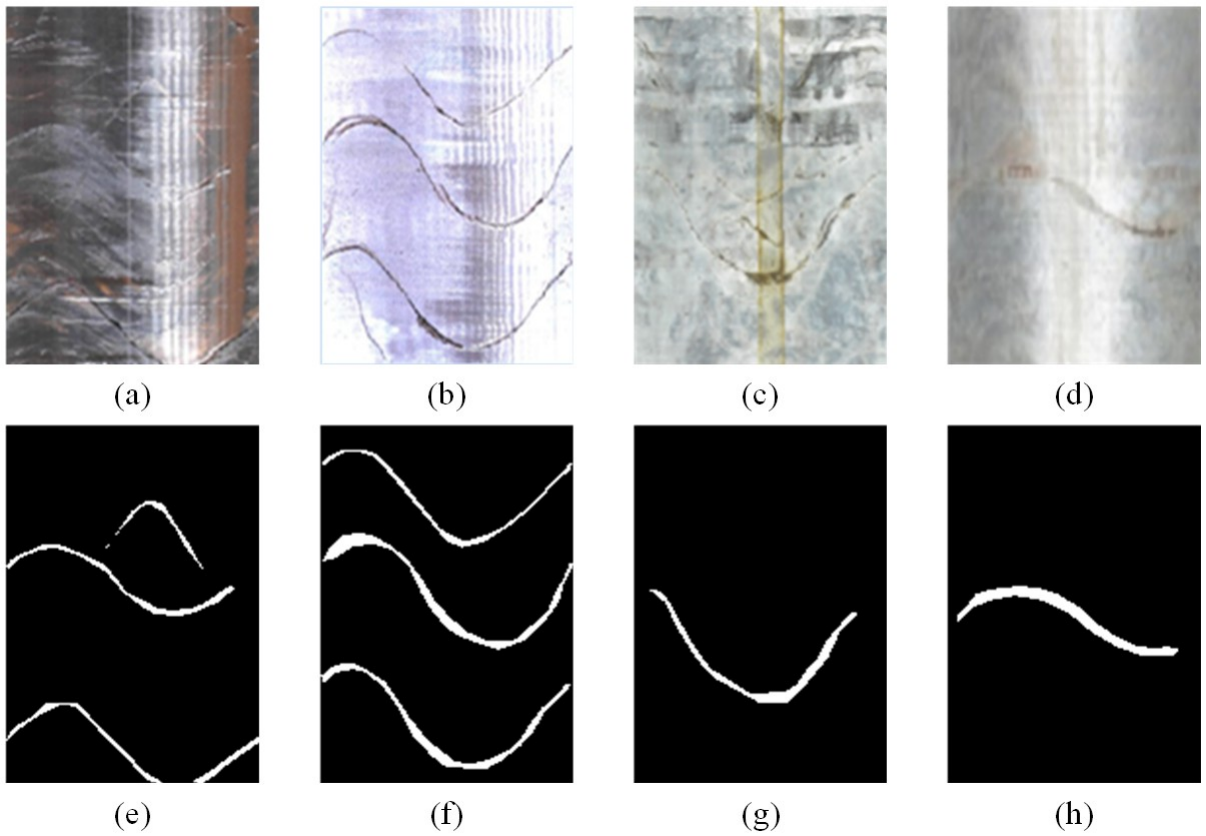
Defect Image Segmentation Effect: (a) Reflective+color anomaly; (b) color anomaly+reflective; (c) local color anomaly; (d) blurry+reflective; (e) Image (a) segmentation result; (f) image (b) segmentation result; (g) image (c) segmentation result; (h) image (d) segmentation result.

Generally, for the segmentation of structural surfaces in borehole images, high-quality and high-resolution images are required from borehole imaging devices. However, the data acquisition environment is complicated, and it is difficult to clean the inner wall of the borehole, with rock powder adhering to the surface, causing image blurring. In some boreholes, the inner wall is smooth and reflective, resulting in local highlights in the generated images. In addition, imaging faults of the equipment can cause abnormal color in the images. Given that borehole image acquisition work is costly and often time-sensitive, segmentation of defective images is necessary. From the segmentation results of the three types of defective images in Fig 12, it can be seen that the method has a good suppression effect on glare interference and vertical stripes in the images, indicating that it has certain adaptability for the segmentation of defective images. The downside is that the shape of the structural surface will change, becoming generally wider.

## Conclusions

1. Constructed a borehole image dataset for training networks for the segmentation of structural surfaces. The dataset includes 1,013 borehole images and uses four data augmentation methods, including image flipping, color jitter, blurring, and mixup to expand the dataset. During training, the number of training samples can be increased to 12,421, enriching the training samples and solving the problem of insufficient samples during training of the structural surface segmentation network.
2. The U^2^-Net semantic segmentation network was used for structural surface segmentation, and through network training and evaluation, the highest F-measure value on the test set reached 0.749, and the precision reached 0.85, achieving complete segmentation of the structural surface area.
3. Due to the long collection cycle and high cost of acquiring borehole images, the image dataset constructed in this paper is slightly insufficient in quantity and structural surface types. Follow-up research should expand the number of borehole images and enrich the included types of structural surfaces to establish a good data foundation for intelligent recognition of structural surfaces. Secondly, although the structural surface recognition method can obtain clear outlines of the structural surfaces, it cannot recognize the specific types of structural surfaces. Follow-up research should reasonably divide the types of structural surfaces and achieve intelligent recognition of the types of structural surfaces.

## Supporting information

S1 File Drill image dataset for training part I. (ZIP)

S2 File Drill image dataset for training part II. (ZIP)
