# Supplementary figures and images for: The segmentation and intelligent recognition of structural surfaces in borehole images based on the U2-Net network (part 2 of 4)
Source: PLoS One. 2024 Mar 7;19(3):e0299471. doi: 10.1371/journal.pone.0299471 (PMC10919631; doi:10.1371/journal.pone.0299471)

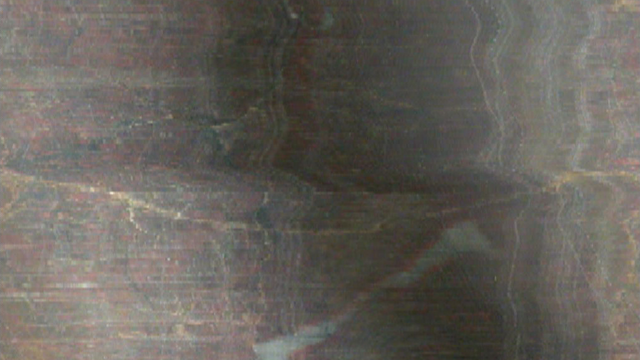

Supplement: S1 File — (ZIP) [file pone.0299471.s001.zip › 0100.png]

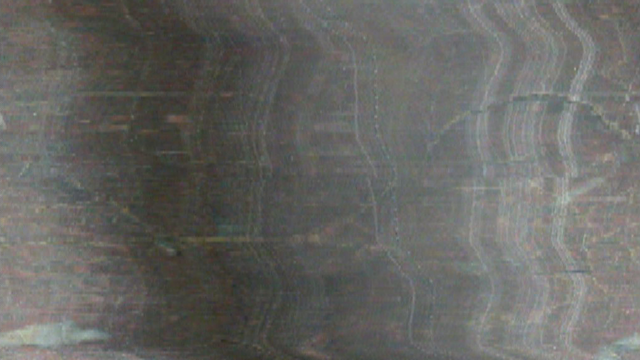

Supplement: S1 File — (ZIP) [file pone.0299471.s001.zip › 0101.png]

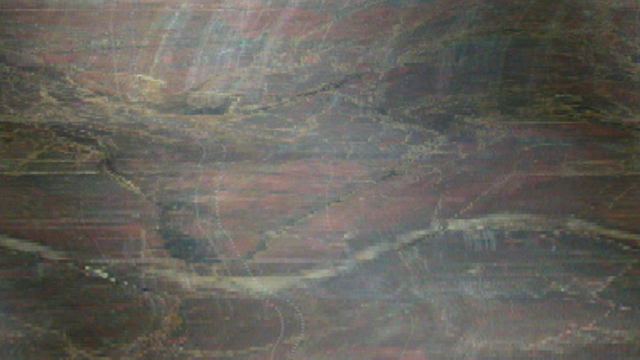

Supplement: S1 File — (ZIP) [file pone.0299471.s001.zip › 0102.png]

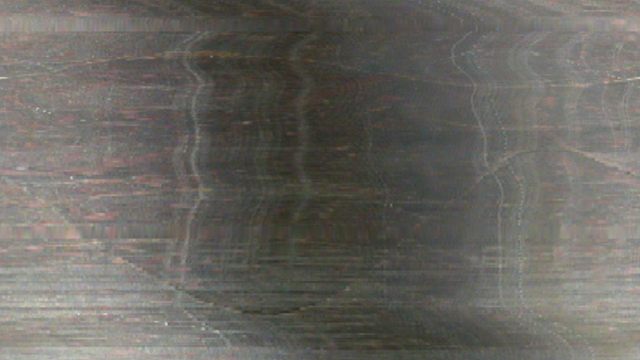

Supplement: S1 File — (ZIP) [file pone.0299471.s001.zip › 0103.png]

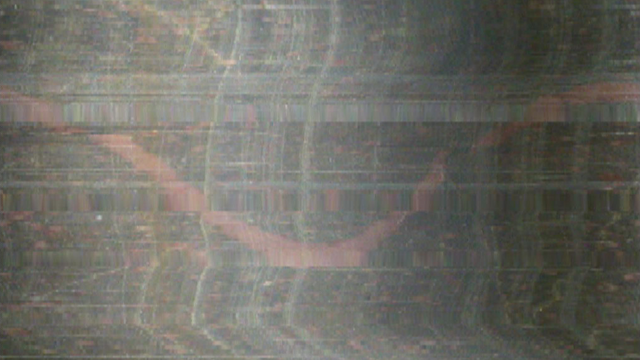

Supplement: S1 File — (ZIP) [file pone.0299471.s001.zip › 0104.png]

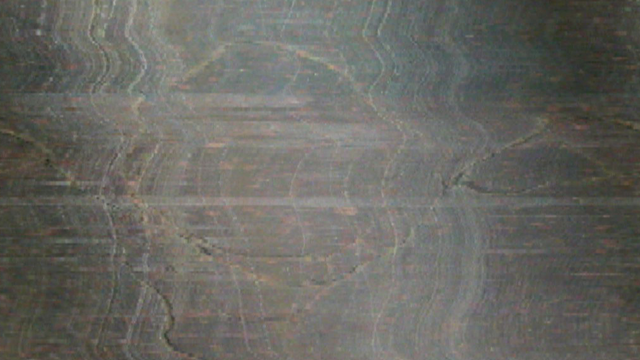

Supplement: S1 File — (ZIP) [file pone.0299471.s001.zip › 0105.png]

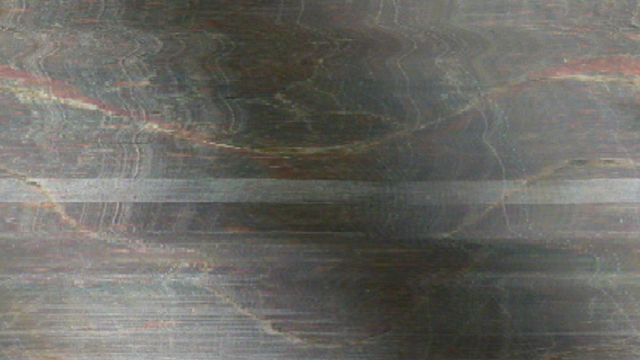

Supplement: S1 File — (ZIP) [file pone.0299471.s001.zip › 0106.png]

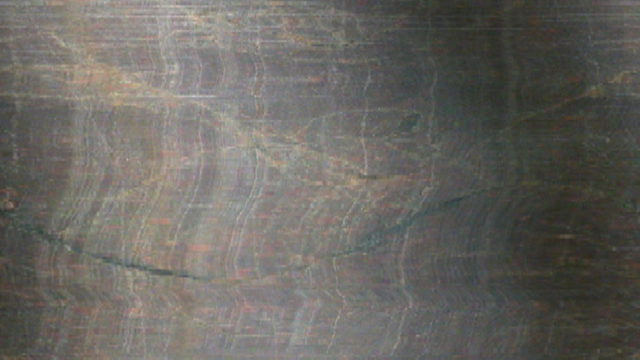

Supplement: S1 File — (ZIP) [file pone.0299471.s001.zip › 0107.png]

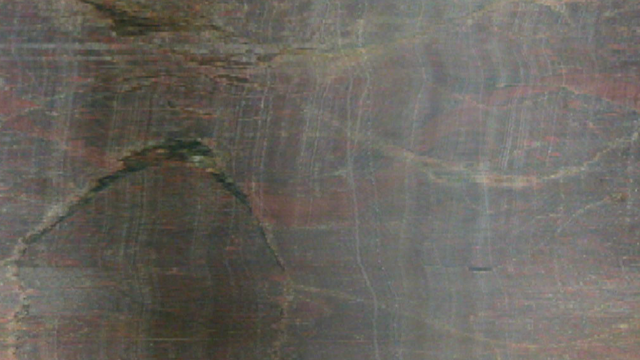

Supplement: S1 File — (ZIP) [file pone.0299471.s001.zip › 0108.png]

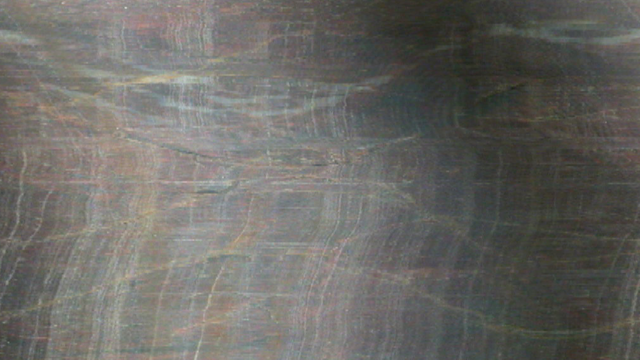

Supplement: S1 File — (ZIP) [file pone.0299471.s001.zip › 0109.png]

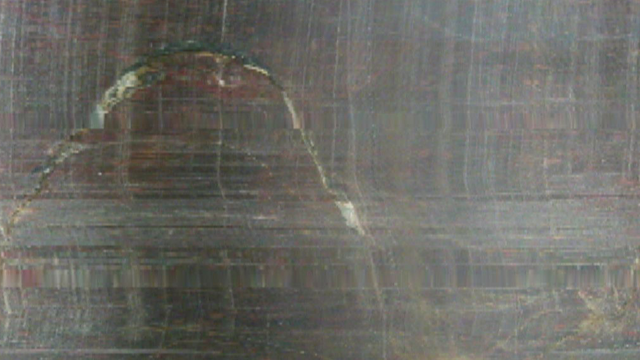

Supplement: S1 File — (ZIP) [file pone.0299471.s001.zip › 0110.png]

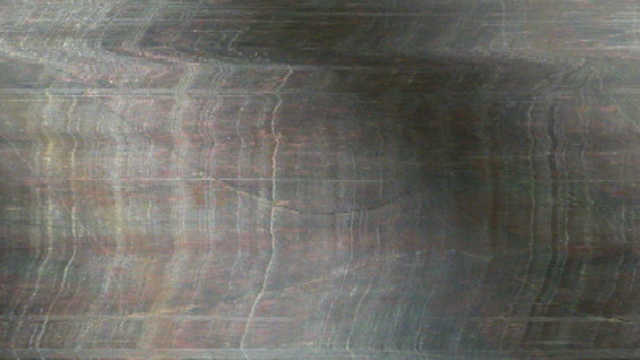

Supplement: S1 File — (ZIP) [file pone.0299471.s001.zip › 0111.png]

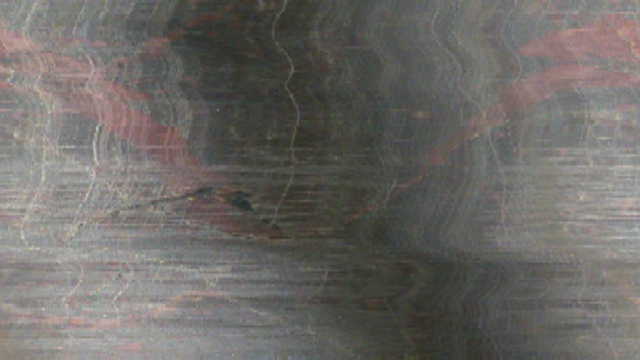

Supplement: S1 File — (ZIP) [file pone.0299471.s001.zip › 0112.png]

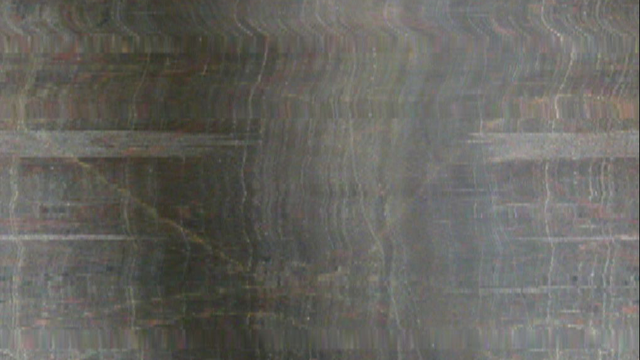

Supplement: S1 File — (ZIP) [file pone.0299471.s001.zip › 0113.png]

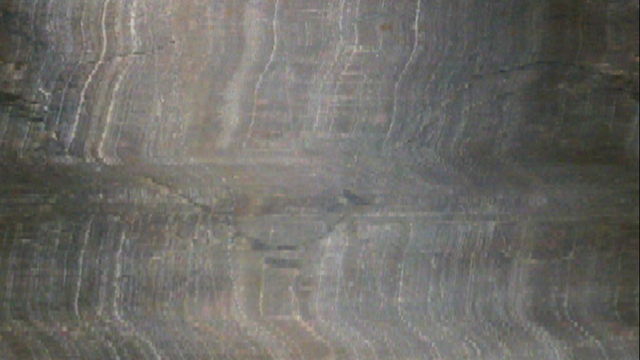

Supplement: S1 File — (ZIP) [file pone.0299471.s001.zip › 0114.png]

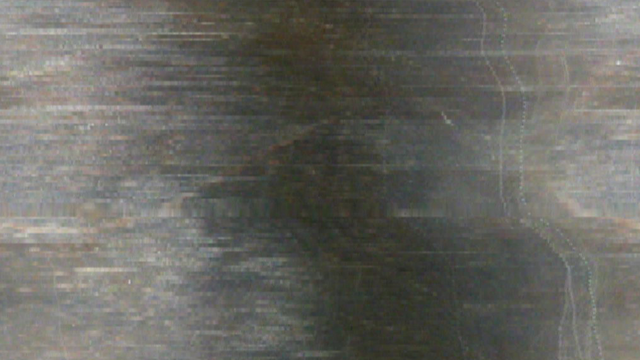

Supplement: S1 File — (ZIP) [file pone.0299471.s001.zip › 0115.png]

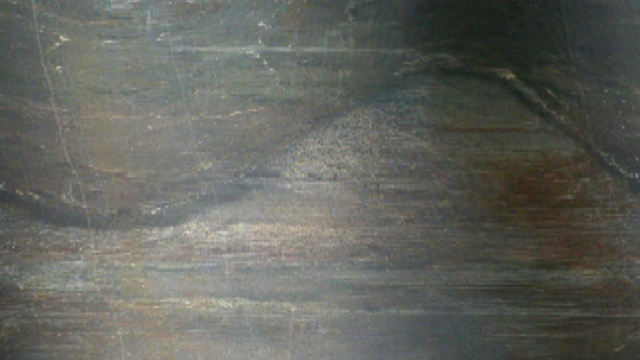

Supplement: S1 File — (ZIP) [file pone.0299471.s001.zip › 0116.png]

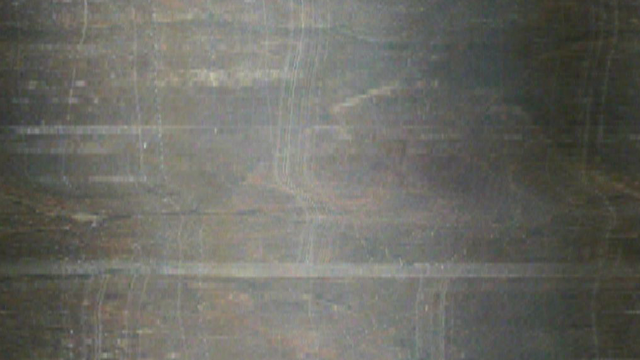

Supplement: S1 File — (ZIP) [file pone.0299471.s001.zip › 0117.png]

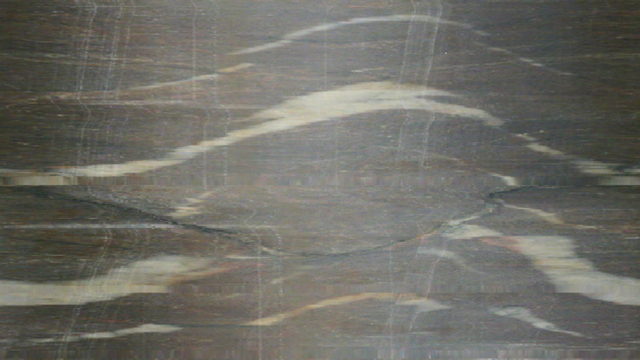

Supplement: S1 File — (ZIP) [file pone.0299471.s001.zip › 0118.png]

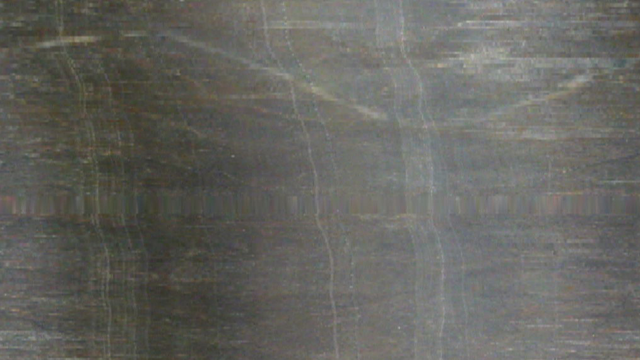

Supplement: S1 File — (ZIP) [file pone.0299471.s001.zip › 0119.png]

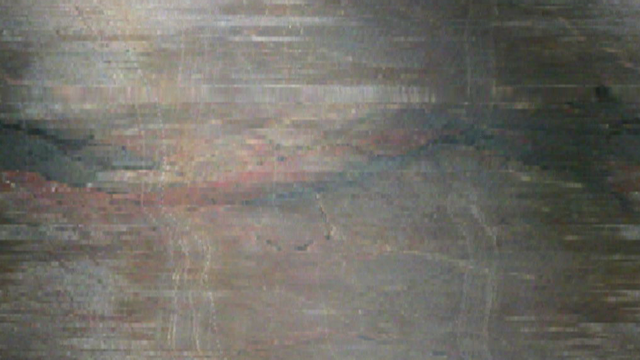

Supplement: S1 File — (ZIP) [file pone.0299471.s001.zip › 0120.png]

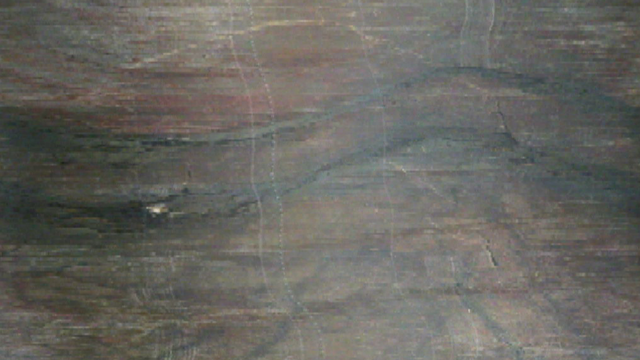

Supplement: S1 File — (ZIP) [file pone.0299471.s001.zip › 0121.png]

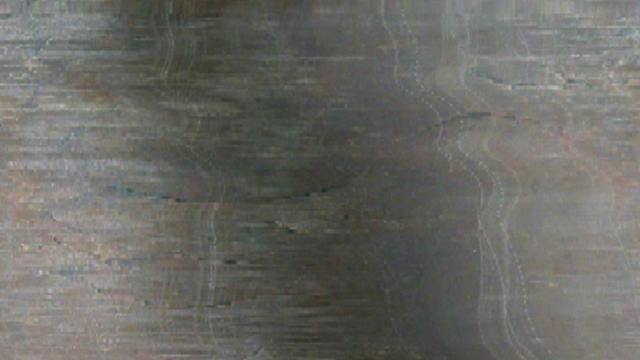

Supplement: S1 File — (ZIP) [file pone.0299471.s001.zip › 0122.png]

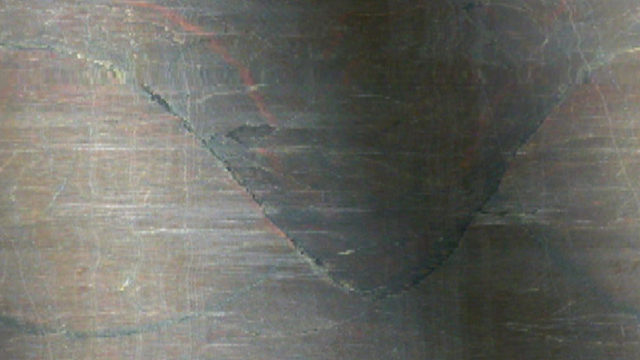

Supplement: S1 File — (ZIP) [file pone.0299471.s001.zip › 0123.png]

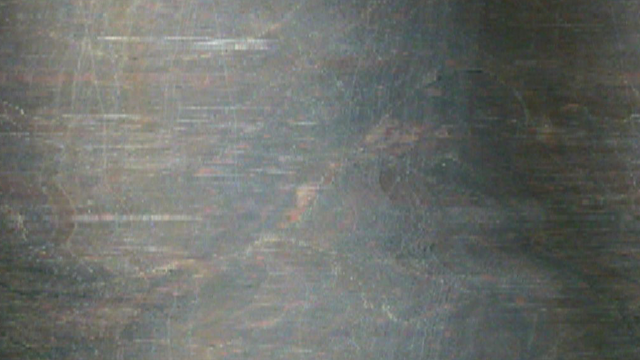

Supplement: S1 File — (ZIP) [file pone.0299471.s001.zip › 0124.png]

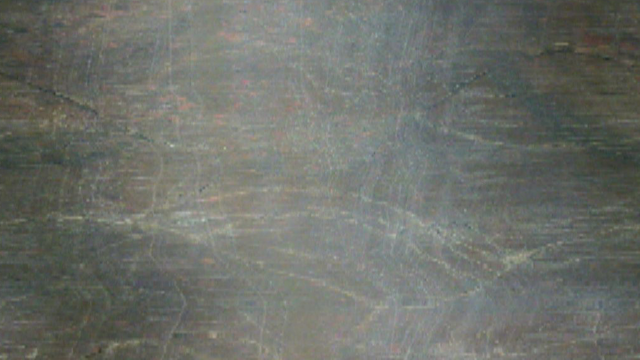

Supplement: S1 File — (ZIP) [file pone.0299471.s001.zip › 0125.png]

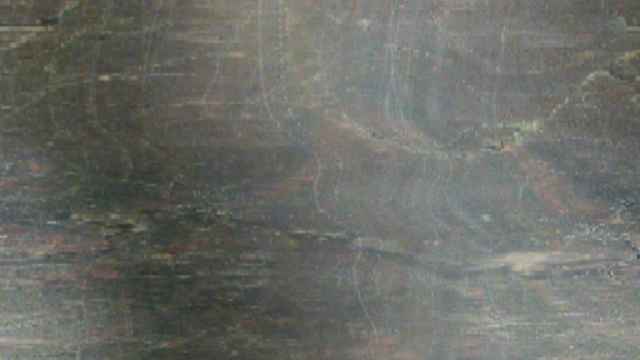

Supplement: S1 File — (ZIP) [file pone.0299471.s001.zip › 0126.png]

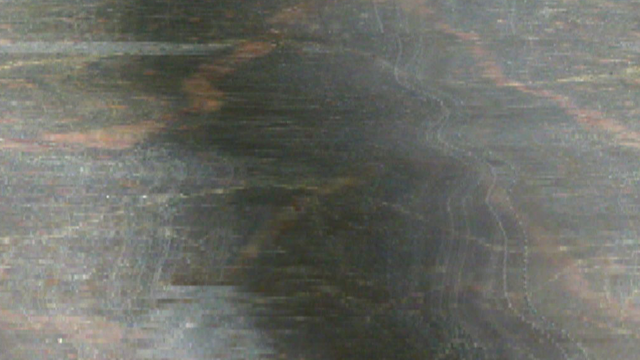

Supplement: S1 File — (ZIP) [file pone.0299471.s001.zip › 0127.png]

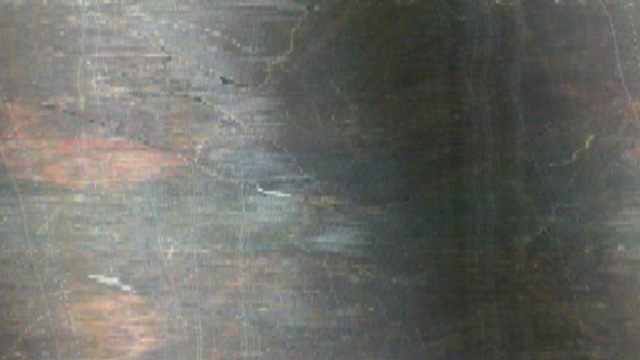

Supplement: S1 File — (ZIP) [file pone.0299471.s001.zip › 0128.png]

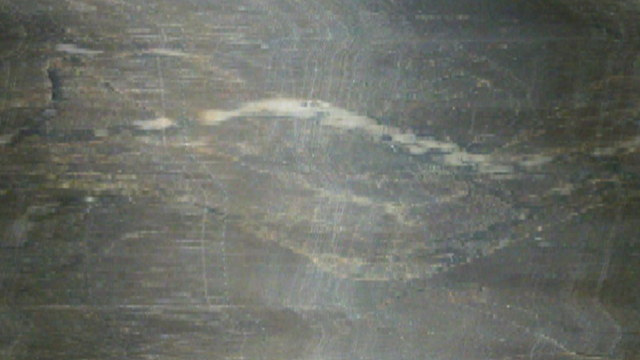

Supplement: S1 File — (ZIP) [file pone.0299471.s001.zip › 0129.png]

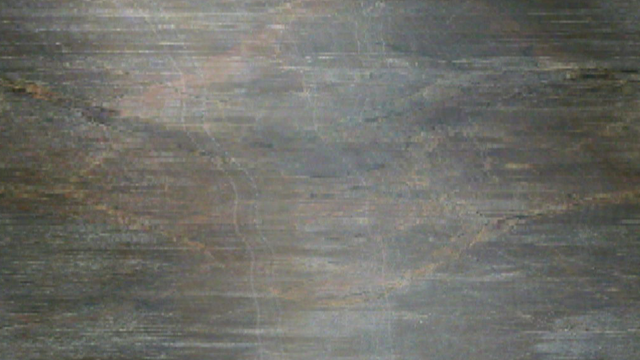

Supplement: S1 File — (ZIP) [file pone.0299471.s001.zip › 0130.png]

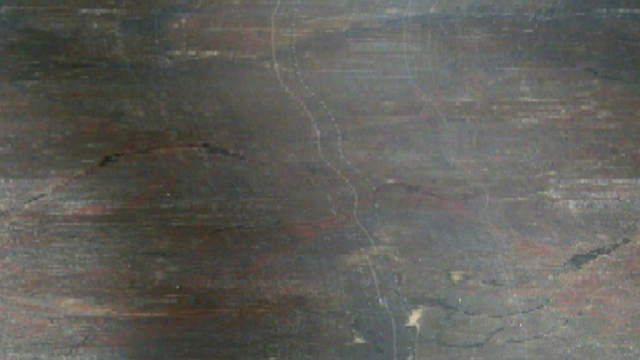

Supplement: S1 File — (ZIP) [file pone.0299471.s001.zip › 0131.png]

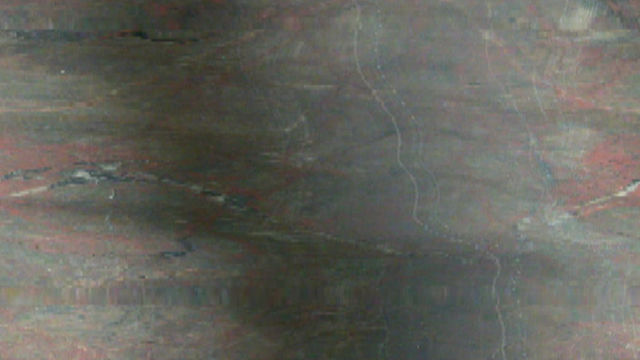

Supplement: S1 File — (ZIP) [file pone.0299471.s001.zip › 0132.png]

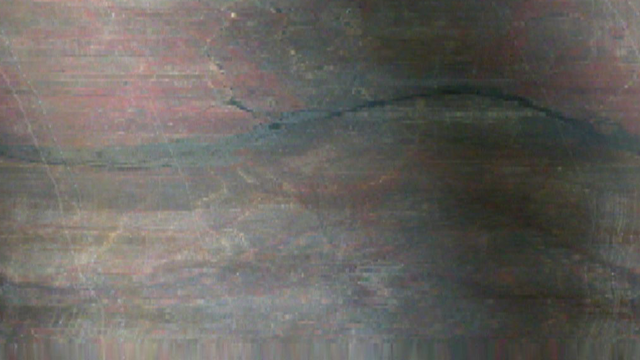

Supplement: S1 File — (ZIP) [file pone.0299471.s001.zip › 0133.png]

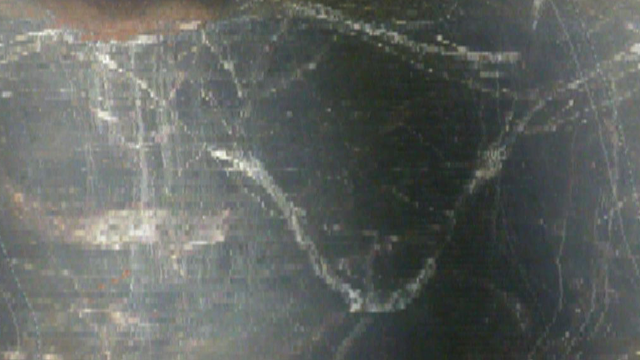

Supplement: S1 File — (ZIP) [file pone.0299471.s001.zip › 0134.png]

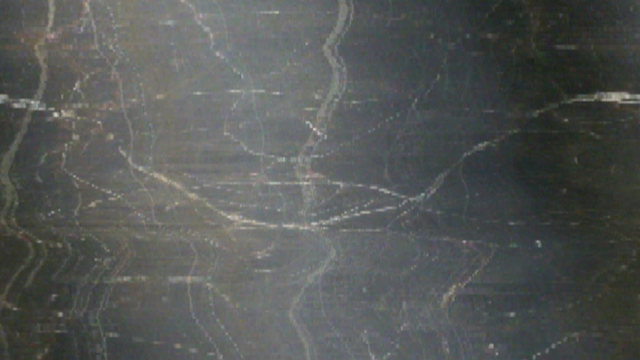

Supplement: S1 File — (ZIP) [file pone.0299471.s001.zip › 0135.png]

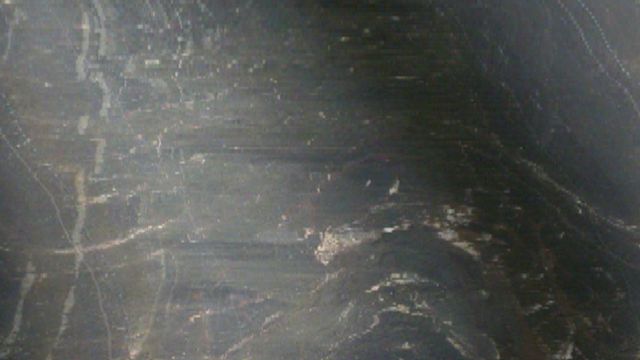

Supplement: S1 File — (ZIP) [file pone.0299471.s001.zip › 0136.png]

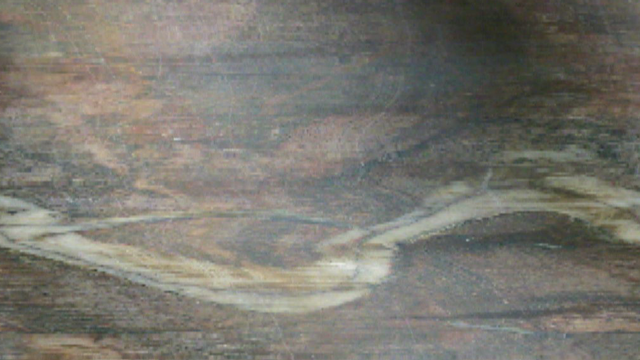

Supplement: S1 File — (ZIP) [file pone.0299471.s001.zip › 0137.png]

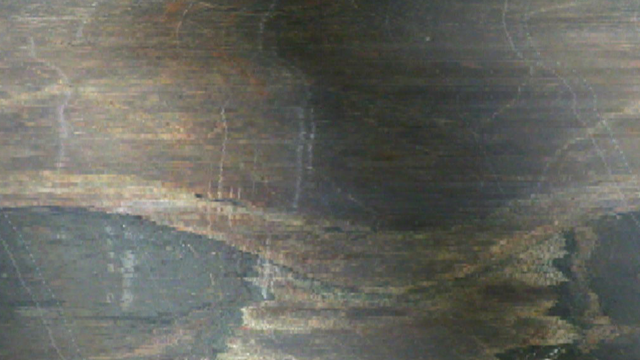

Supplement: S1 File — (ZIP) [file pone.0299471.s001.zip › 0138.png]

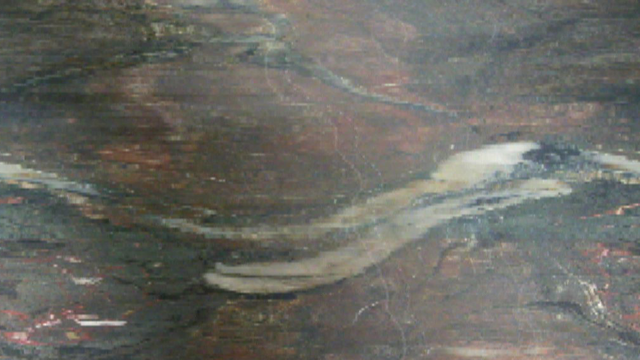

Supplement: S1 File — (ZIP) [file pone.0299471.s001.zip › 0139.png]

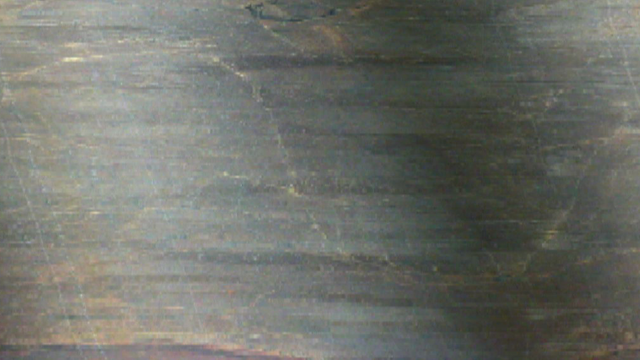

Supplement: S1 File — (ZIP) [file pone.0299471.s001.zip › 0140.png]

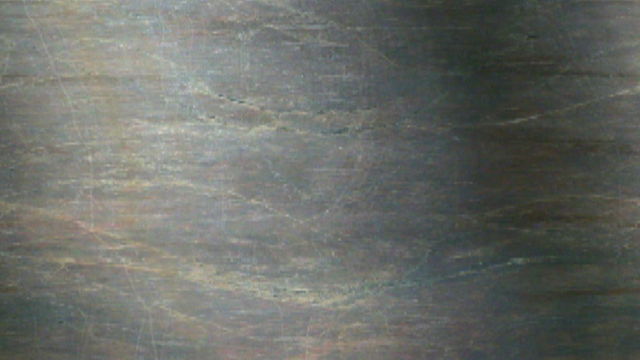

Supplement: S1 File — (ZIP) [file pone.0299471.s001.zip › 0141.png]

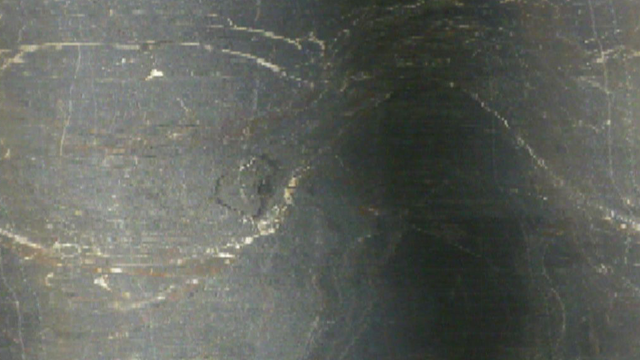

Supplement: S1 File — (ZIP) [file pone.0299471.s001.zip › 0142.png]

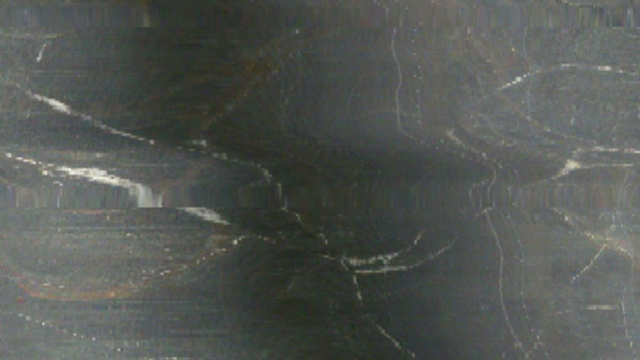

Supplement: S1 File — (ZIP) [file pone.0299471.s001.zip › 0143.png]

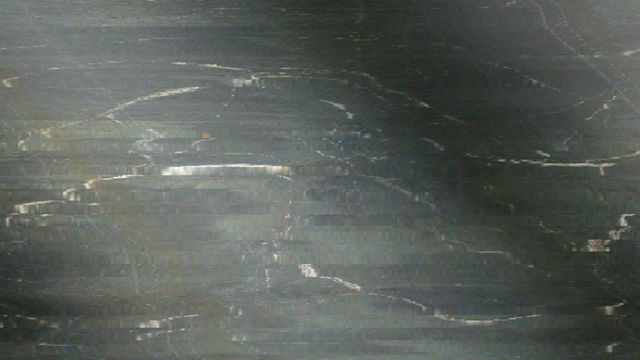

Supplement: S1 File — (ZIP) [file pone.0299471.s001.zip › 0144.png]

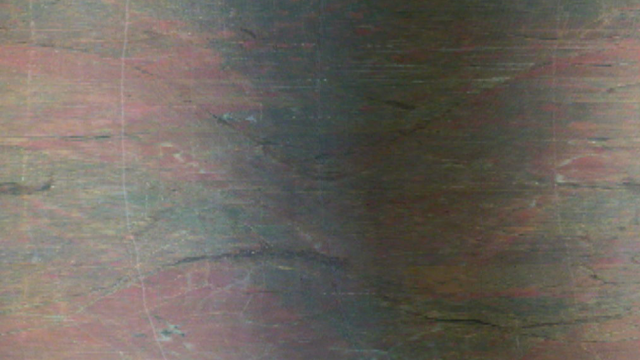

Supplement: S1 File — (ZIP) [file pone.0299471.s001.zip › 0145.png]

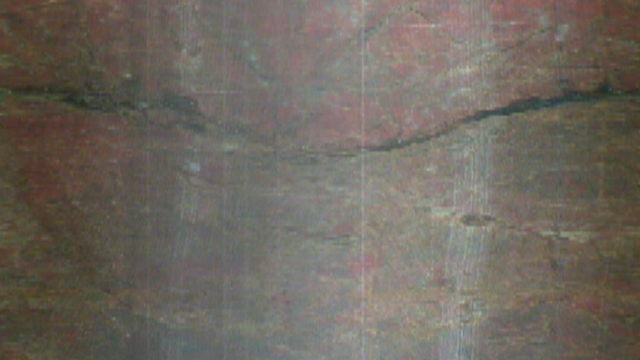

Supplement: S1 File — (ZIP) [file pone.0299471.s001.zip › 0146.png]

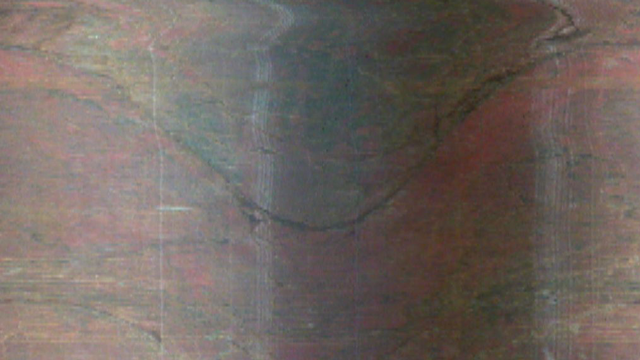

Supplement: S1 File — (ZIP) [file pone.0299471.s001.zip › 0147.png]

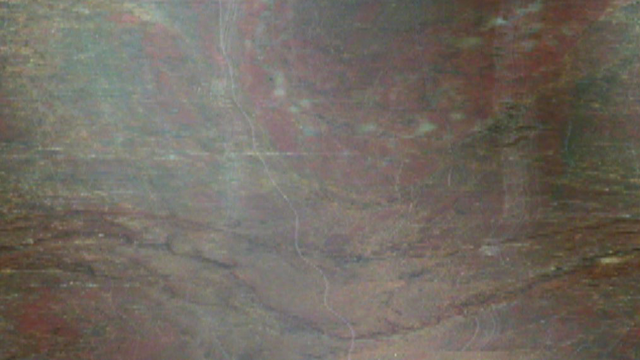

Supplement: S1 File — (ZIP) [file pone.0299471.s001.zip › 0148.png]

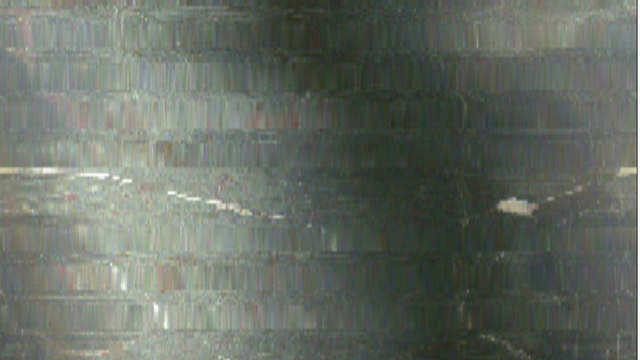

Supplement: S1 File — (ZIP) [file pone.0299471.s001.zip › 0149.png]

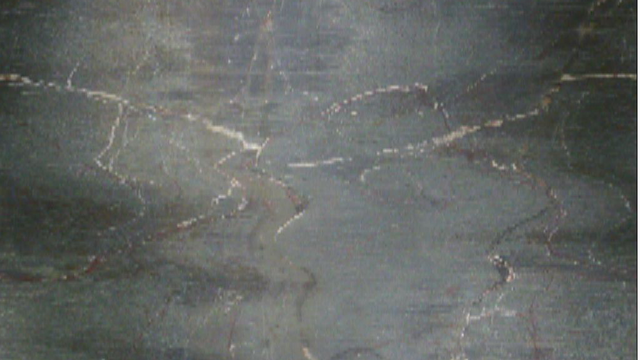

Supplement: S1 File — (ZIP) [file pone.0299471.s001.zip › 0150.png]

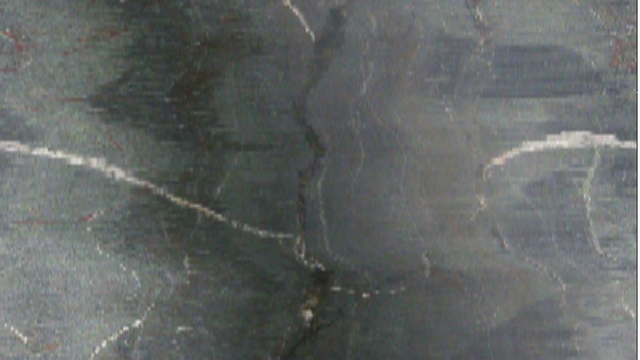

Supplement: S1 File — (ZIP) [file pone.0299471.s001.zip › 0151.png]

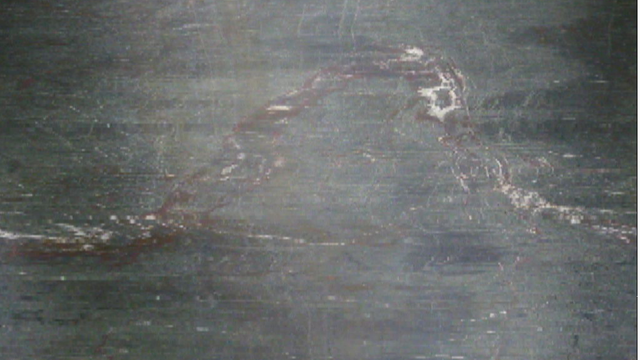

Supplement: S1 File — (ZIP) [file pone.0299471.s001.zip › 0152.png]

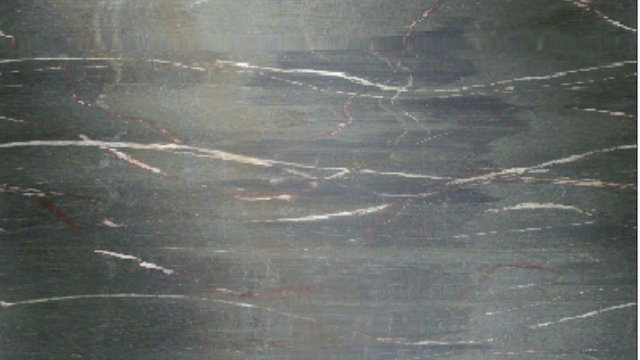

Supplement: S1 File — (ZIP) [file pone.0299471.s001.zip › 0153.png]

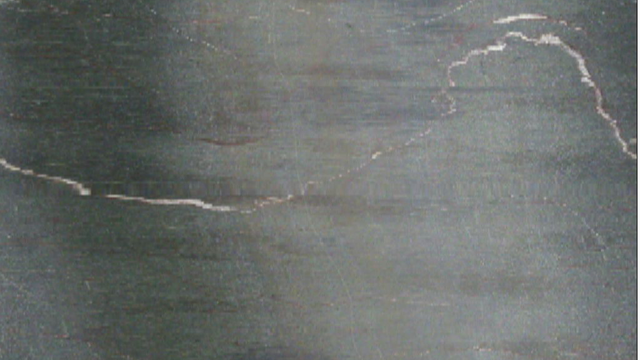

Supplement: S1 File — (ZIP) [file pone.0299471.s001.zip › 0154.png]

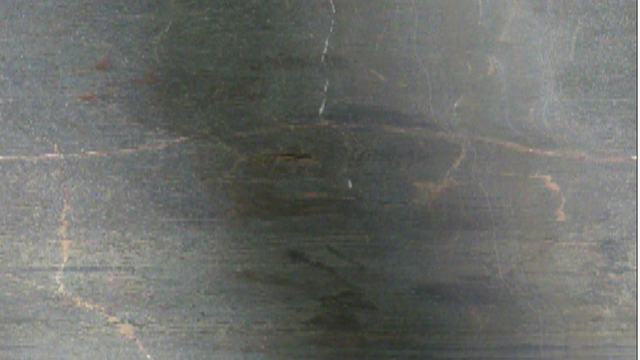

Supplement: S1 File — (ZIP) [file pone.0299471.s001.zip › 0155.png]

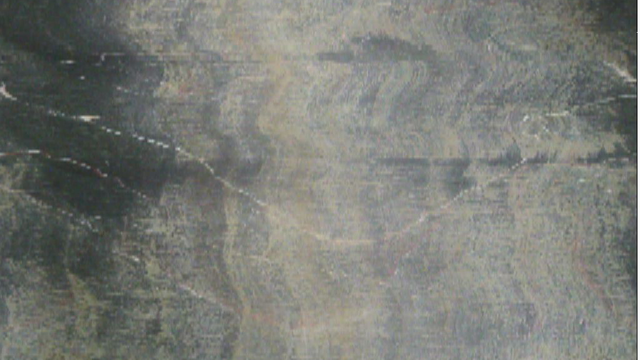

Supplement: S1 File — (ZIP) [file pone.0299471.s001.zip › 0156.png]

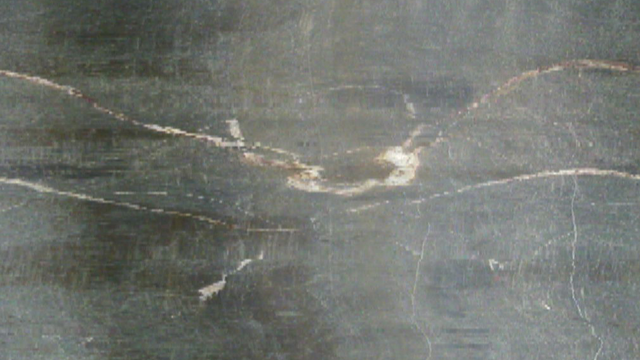

Supplement: S1 File — (ZIP) [file pone.0299471.s001.zip › 0157.png]

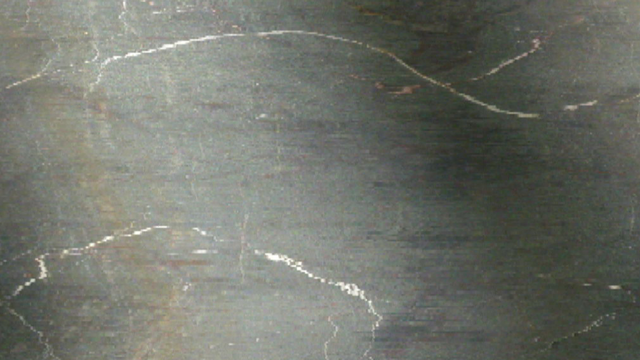

Supplement: S1 File — (ZIP) [file pone.0299471.s001.zip › 0158.png]

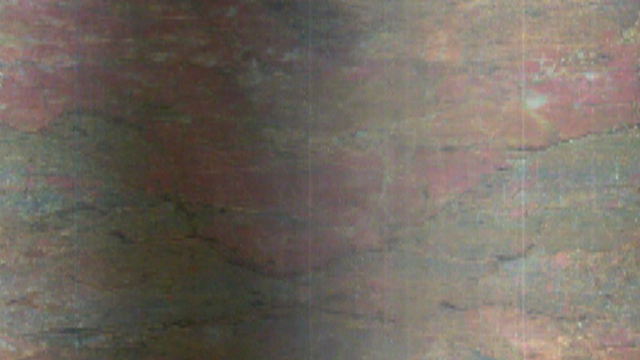

Supplement: S1 File — (ZIP) [file pone.0299471.s001.zip › 0159.png]

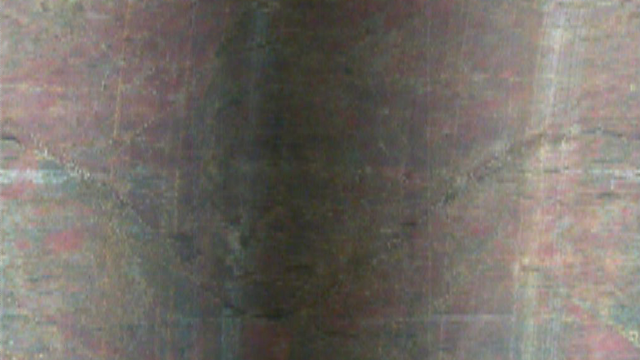

Supplement: S1 File — (ZIP) [file pone.0299471.s001.zip › 0160.png]

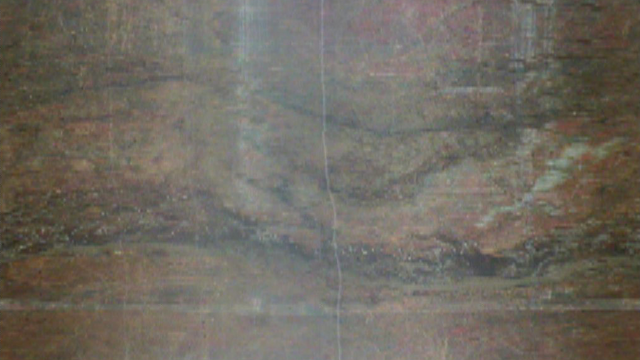

Supplement: S1 File — (ZIP) [file pone.0299471.s001.zip › 0161.png]

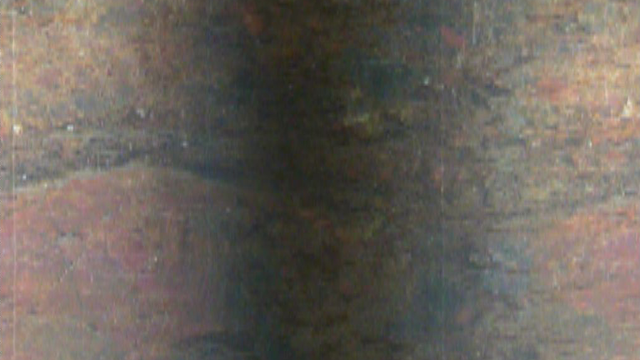

Supplement: S1 File — (ZIP) [file pone.0299471.s001.zip › 0162.png]

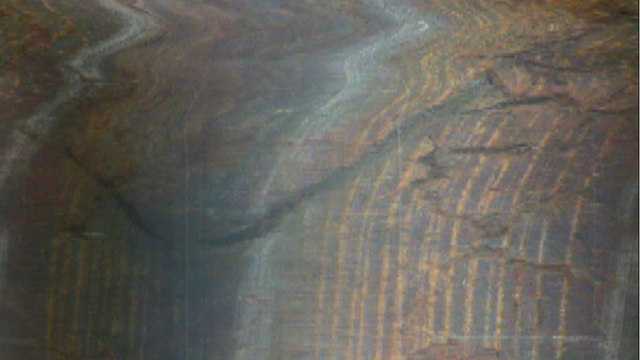

Supplement: S1 File — (ZIP) [file pone.0299471.s001.zip › 0163.png]

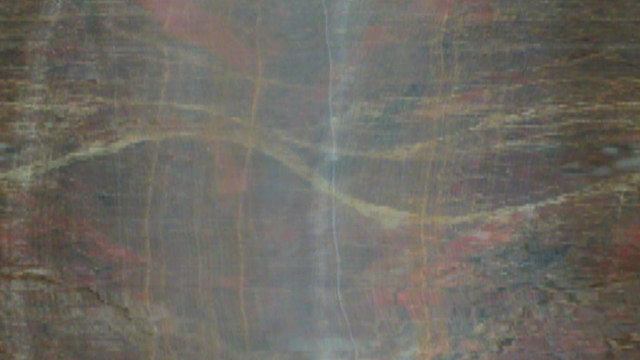

Supplement: S1 File — (ZIP) [file pone.0299471.s001.zip › 0164.png]

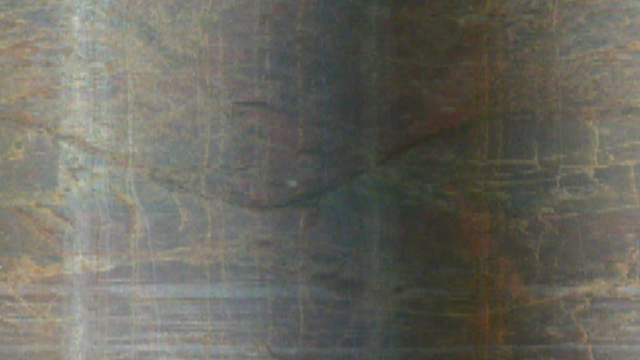

Supplement: S1 File — (ZIP) [file pone.0299471.s001.zip › 0165.png]

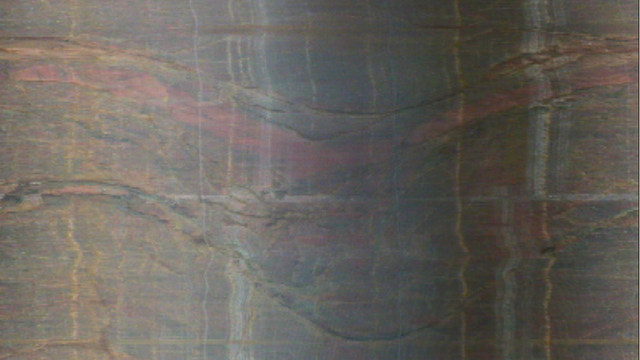

Supplement: S1 File — (ZIP) [file pone.0299471.s001.zip › 0166.png]

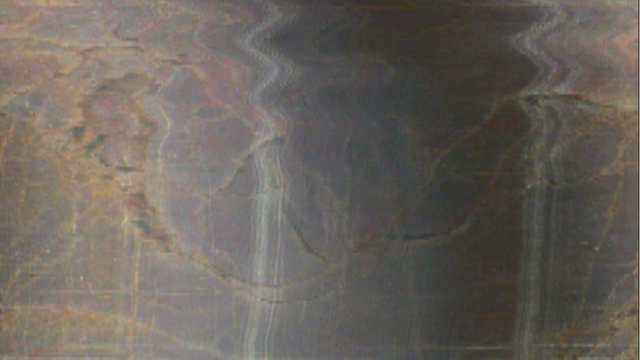

Supplement: S1 File — (ZIP) [file pone.0299471.s001.zip › 0167.png]

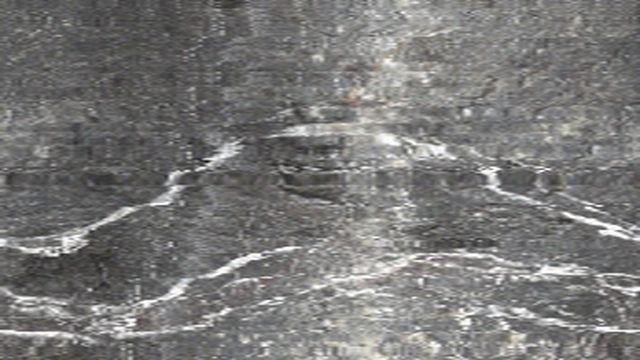

Supplement: S1 File — (ZIP) [file pone.0299471.s001.zip › 0168.png]

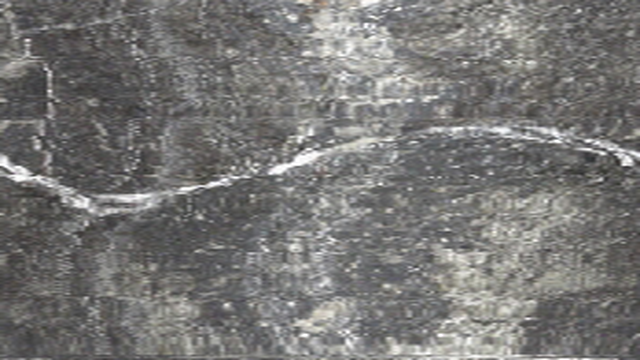

Supplement: S1 File — (ZIP) [file pone.0299471.s001.zip › 0169.png]

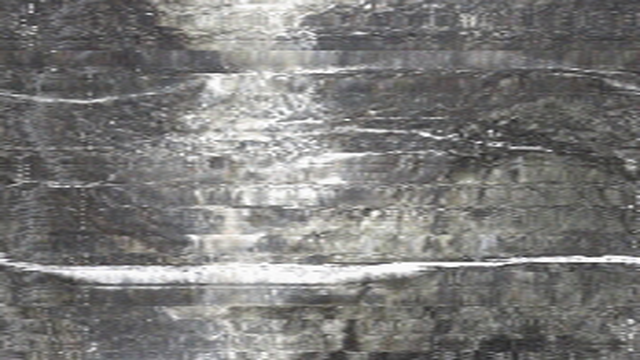

Supplement: S1 File — (ZIP) [file pone.0299471.s001.zip › 0170.png]

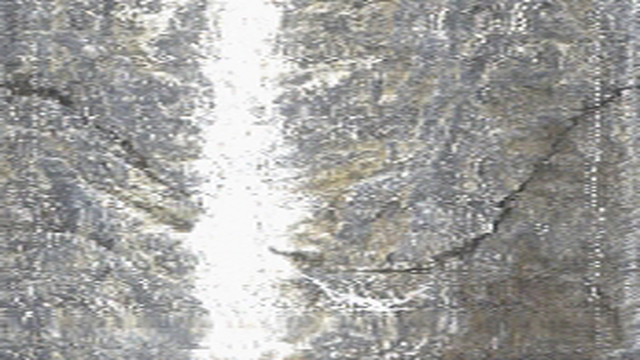

Supplement: S1 File — (ZIP) [file pone.0299471.s001.zip › 0171.png]

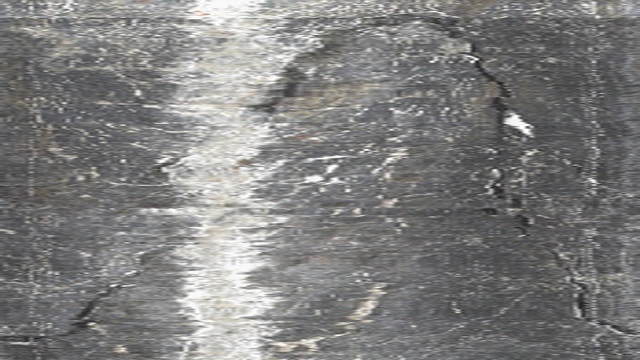

Supplement: S1 File — (ZIP) [file pone.0299471.s001.zip › 0172.png]

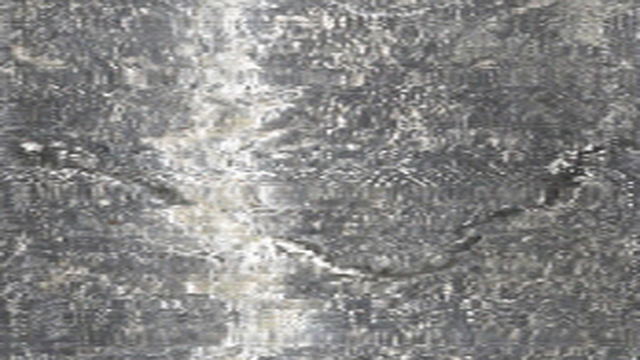

Supplement: S1 File — (ZIP) [file pone.0299471.s001.zip › 0173.png]

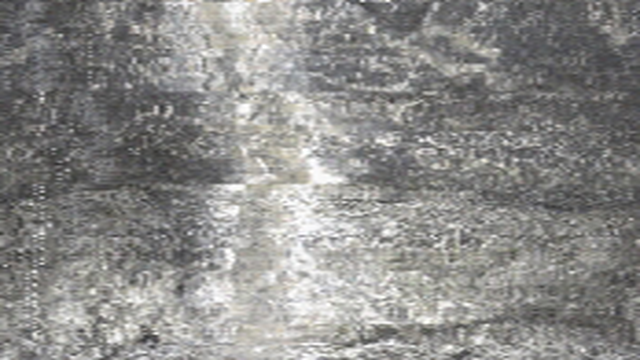

Supplement: S1 File — (ZIP) [file pone.0299471.s001.zip › 0174.png]

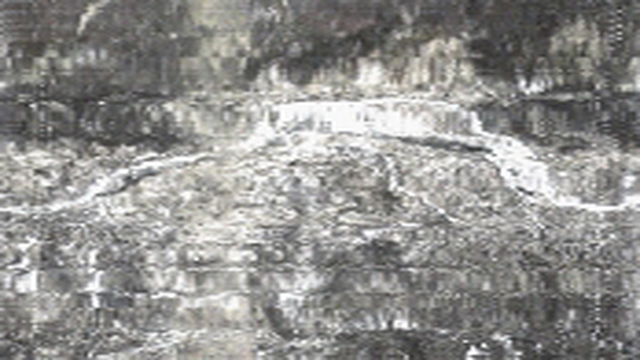

Supplement: S1 File — (ZIP) [file pone.0299471.s001.zip › 0175.png]

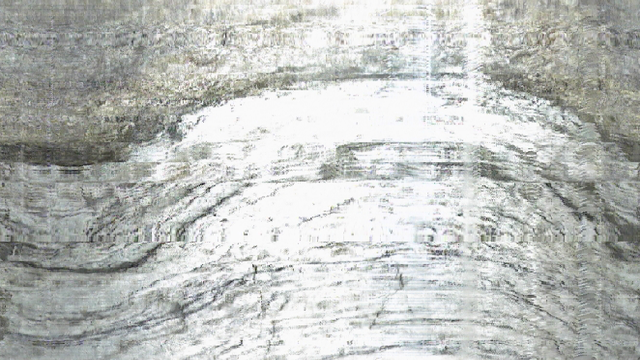

Supplement: S1 File — (ZIP) [file pone.0299471.s001.zip › 0176.png]

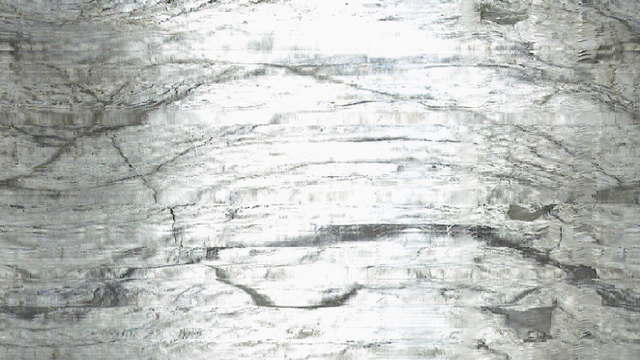

Supplement: S1 File — (ZIP) [file pone.0299471.s001.zip › 0177.png]

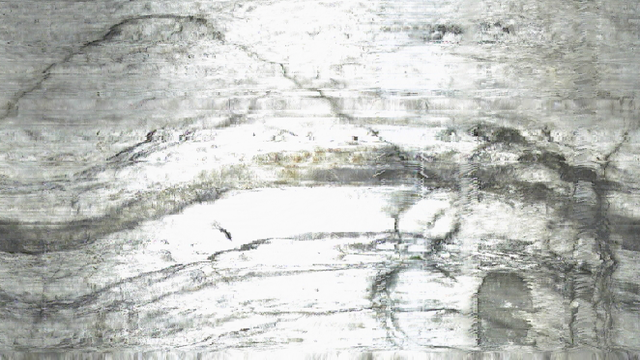

Supplement: S1 File — (ZIP) [file pone.0299471.s001.zip › 0178.png]

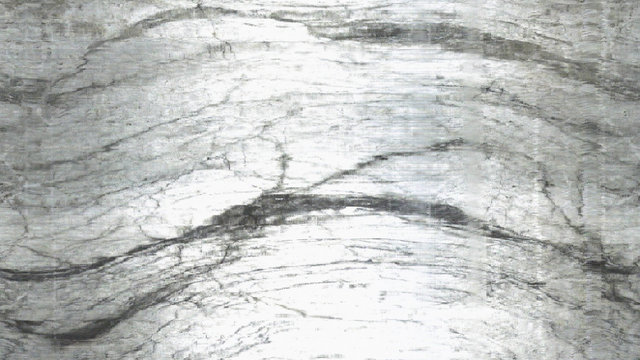

Supplement: S1 File — (ZIP) [file pone.0299471.s001.zip › 0179.png]

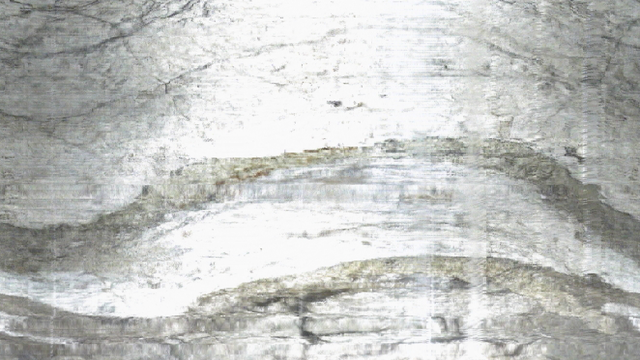

Supplement: S1 File — (ZIP) [file pone.0299471.s001.zip › 0180.png]

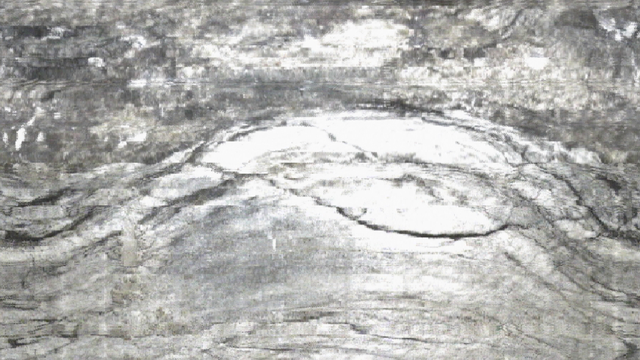

Supplement: S1 File — (ZIP) [file pone.0299471.s001.zip › 0181.png]

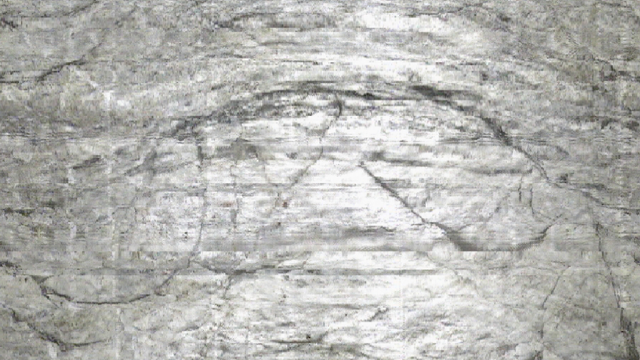

Supplement: S1 File — (ZIP) [file pone.0299471.s001.zip › 0182.png]

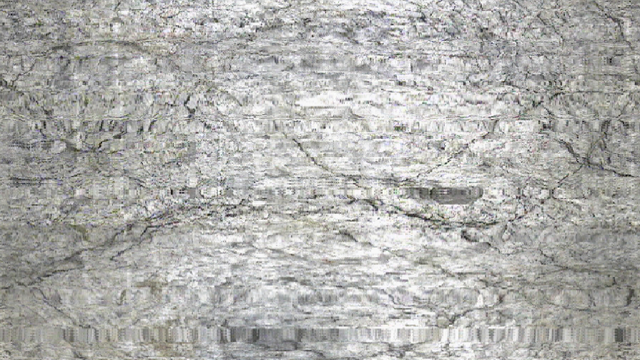

Supplement: S1 File — (ZIP) [file pone.0299471.s001.zip › 0183.png]

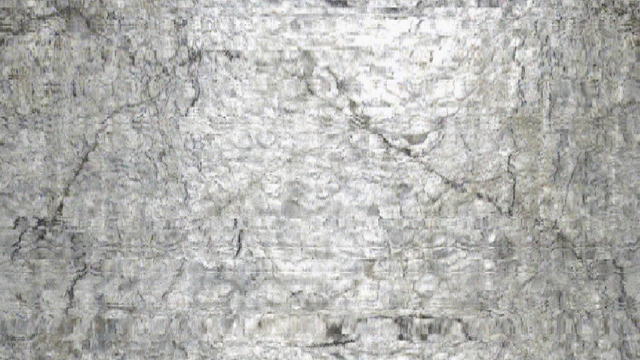

Supplement: S1 File — (ZIP) [file pone.0299471.s001.zip › 0184.png]

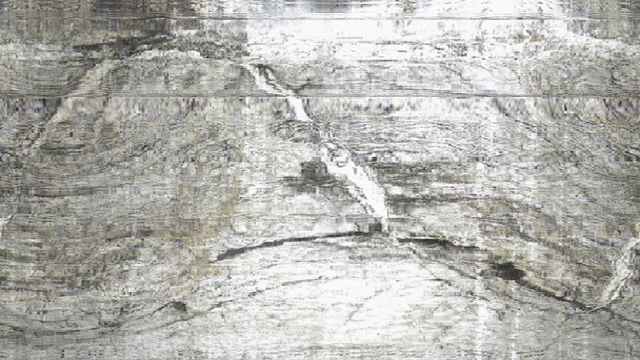

Supplement: S1 File — (ZIP) [file pone.0299471.s001.zip › 0185.png]

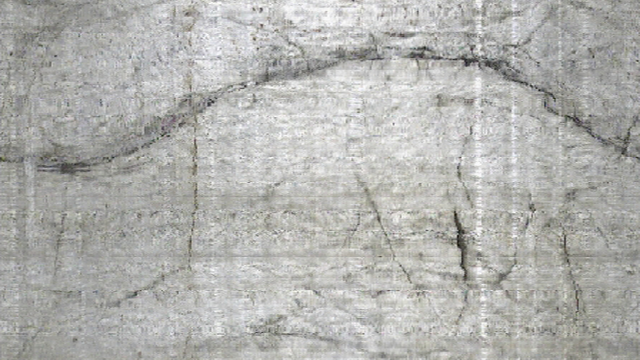

Supplement: S1 File — (ZIP) [file pone.0299471.s001.zip › 0186.png]

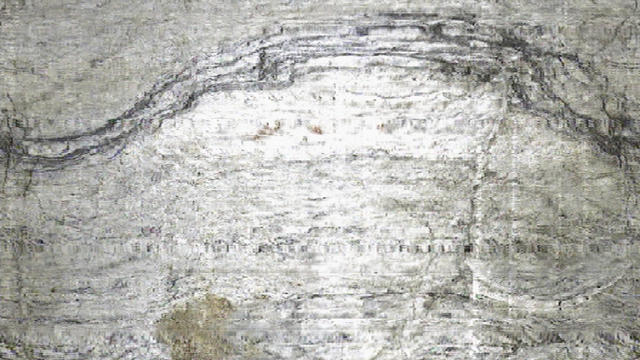

Supplement: S1 File — (ZIP) [file pone.0299471.s001.zip › 0187.png]

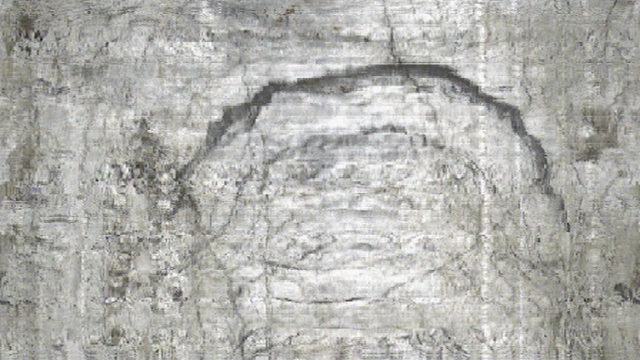

Supplement: S1 File — (ZIP) [file pone.0299471.s001.zip › 0188.png]

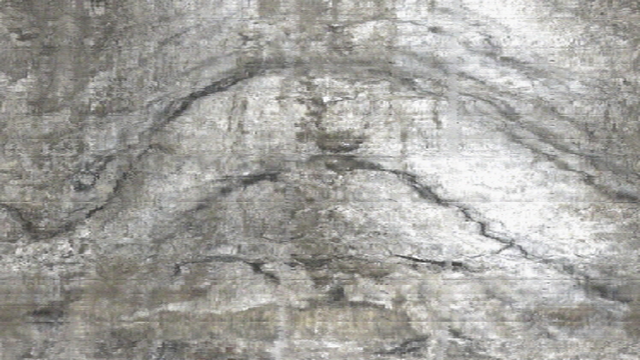

Supplement: S1 File — (ZIP) [file pone.0299471.s001.zip › 0189.png]

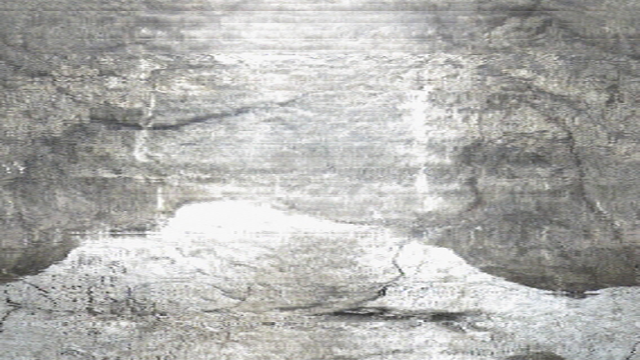

Supplement: S1 File — (ZIP) [file pone.0299471.s001.zip › 0190.png]

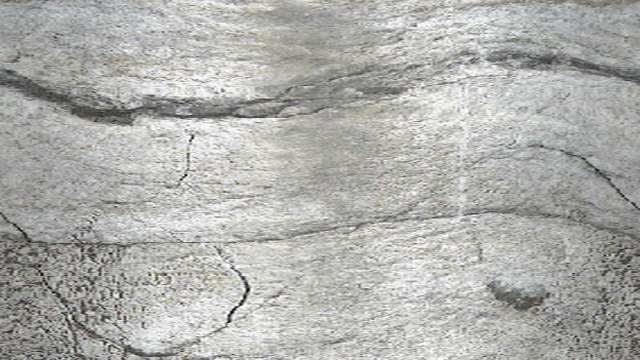

Supplement: S1 File — (ZIP) [file pone.0299471.s001.zip › 0191.png]

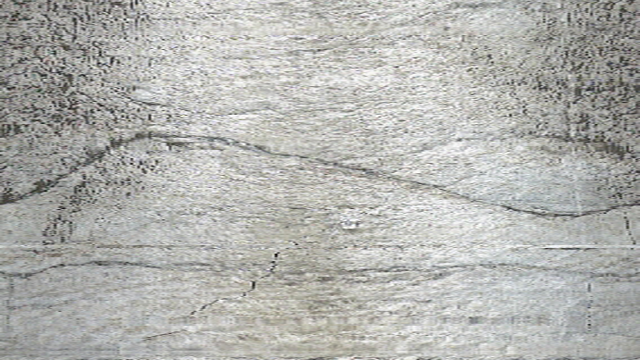

Supplement: S1 File — (ZIP) [file pone.0299471.s001.zip › 0192.png]

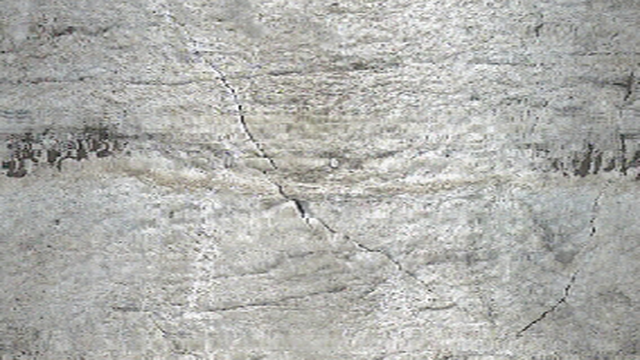

Supplement: S1 File — (ZIP) [file pone.0299471.s001.zip › 0193.png]

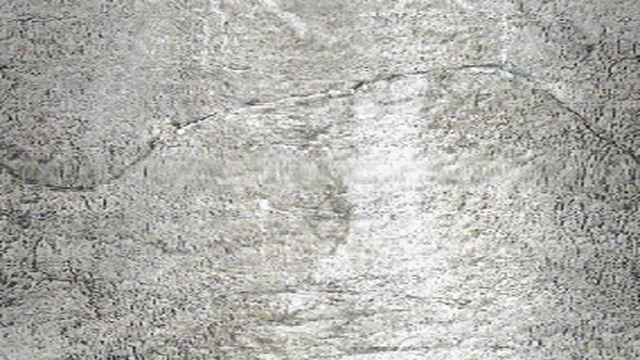

Supplement: S1 File — (ZIP) [file pone.0299471.s001.zip › 0194.png]

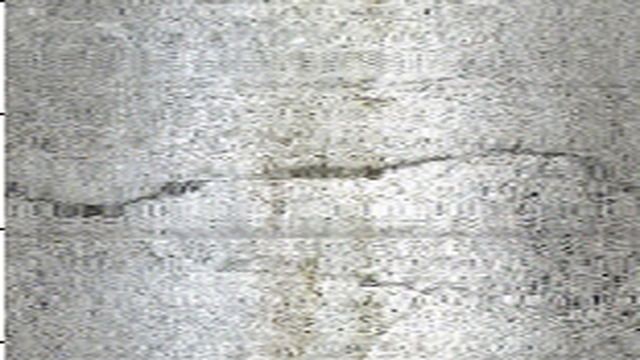

Supplement: S1 File — (ZIP) [file pone.0299471.s001.zip › 0195.png]

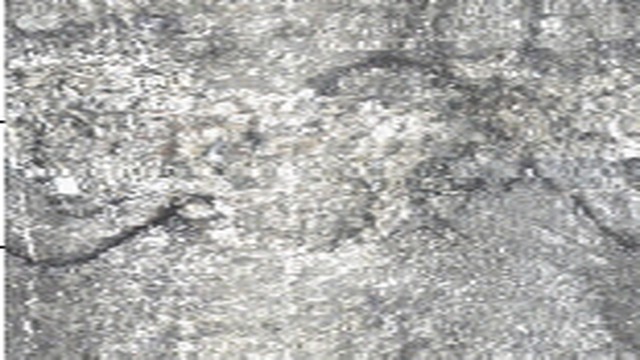

Supplement: S1 File — (ZIP) [file pone.0299471.s001.zip › 0196.png]

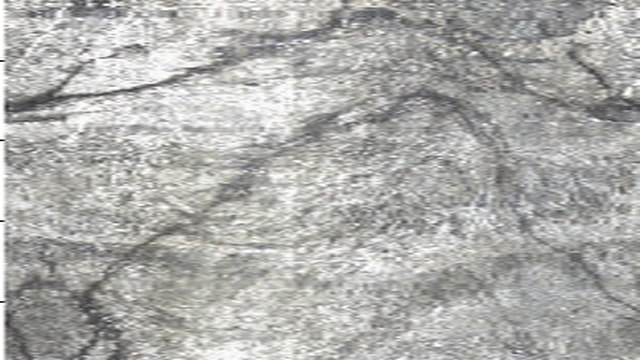

Supplement: S1 File — (ZIP) [file pone.0299471.s001.zip › 0197.png]

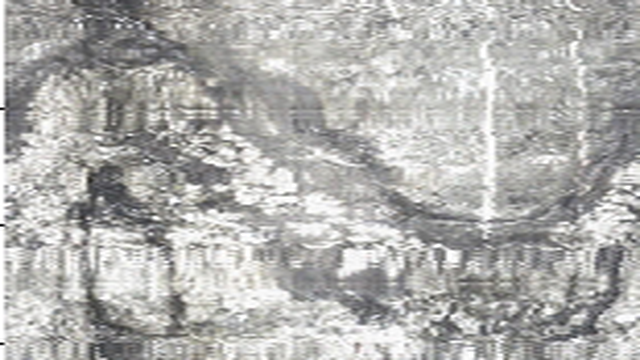

Supplement: S2 File — (ZIP) [file pone.0299471.s002.zip › 0198.png]

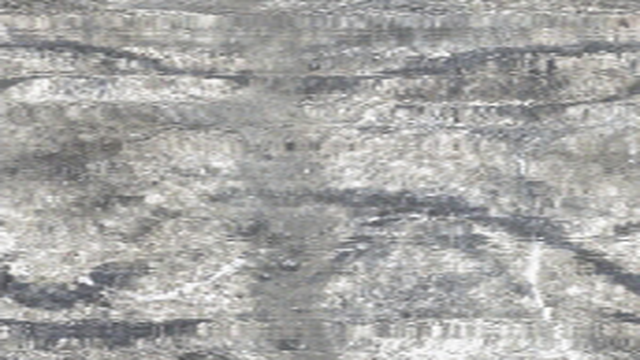

Supplement: S2 File — (ZIP) [file pone.0299471.s002.zip › 0199.png]
