# Supplementary figures and images for: The segmentation and intelligent recognition of structural surfaces in borehole images based on the U2-Net network (part 1 of 4)
Source: PLoS One. 2024 Mar 7;19(3):e0299471. doi: 10.1371/journal.pone.0299471 (PMC10919631; doi:10.1371/journal.pone.0299471)

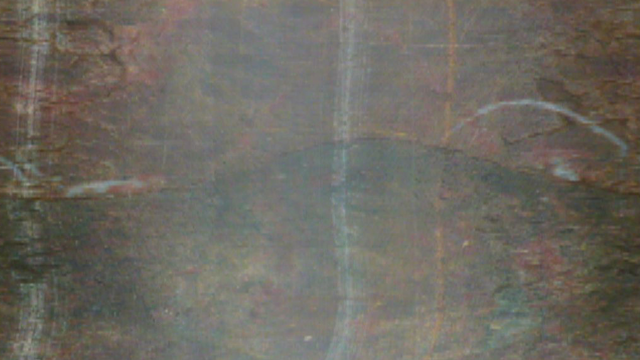

Supplement: S1 File — (ZIP) [file pone.0299471.s001.zip › 0000.png]

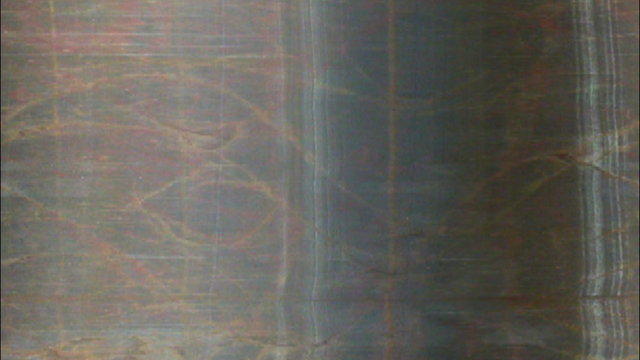

Supplement: S1 File — (ZIP) [file pone.0299471.s001.zip › 0001.png]

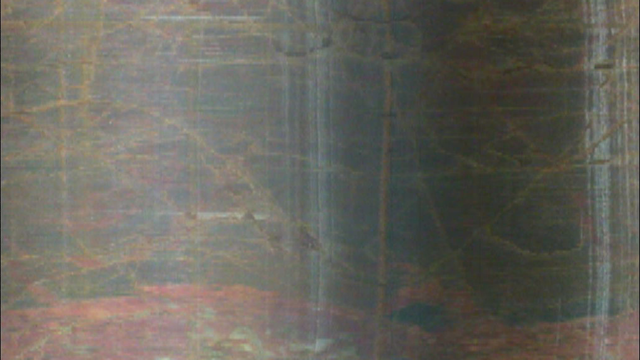

Supplement: S1 File — (ZIP) [file pone.0299471.s001.zip › 0002.png]

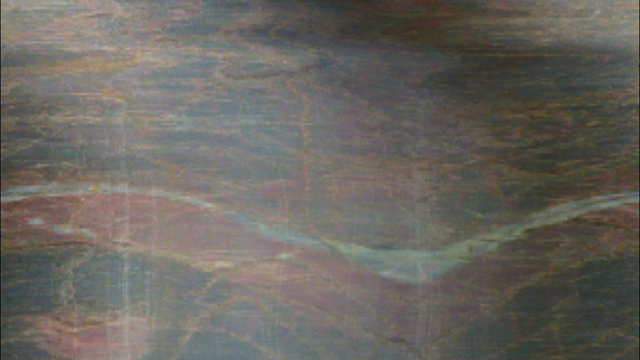

Supplement: S1 File — (ZIP) [file pone.0299471.s001.zip › 0003.png]

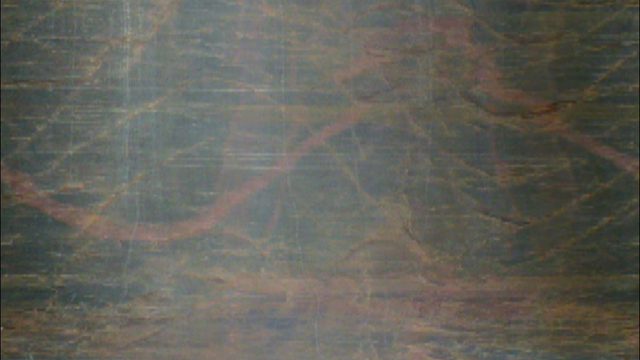

Supplement: S1 File — (ZIP) [file pone.0299471.s001.zip › 0004.png]

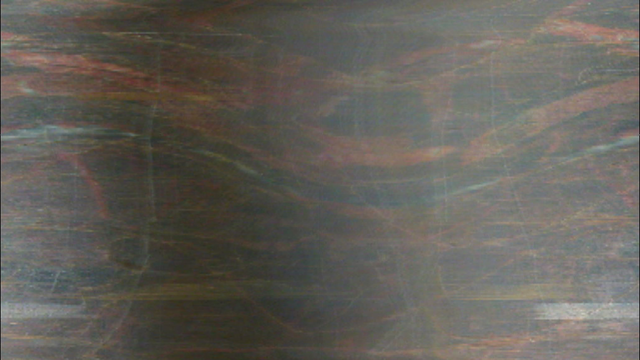

Supplement: S1 File — (ZIP) [file pone.0299471.s001.zip › 0005.png]

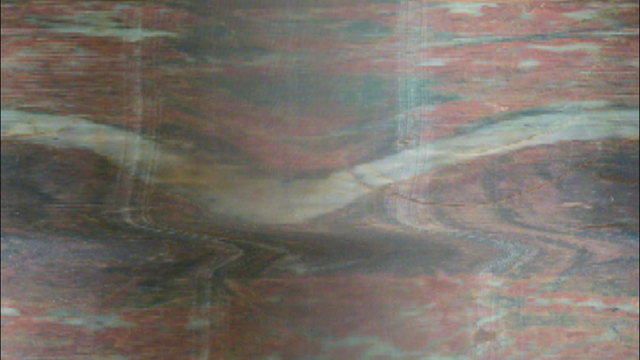

Supplement: S1 File — (ZIP) [file pone.0299471.s001.zip › 0006.png]

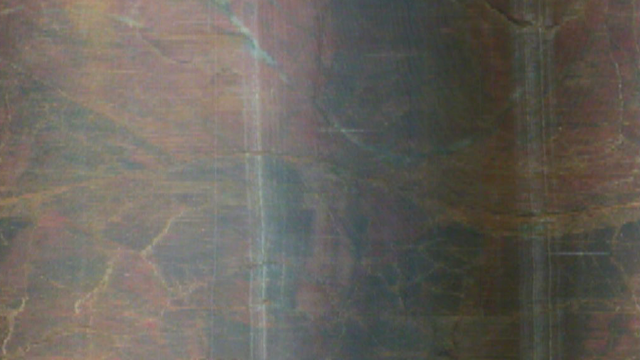

Supplement: S1 File — (ZIP) [file pone.0299471.s001.zip › 0007.png]

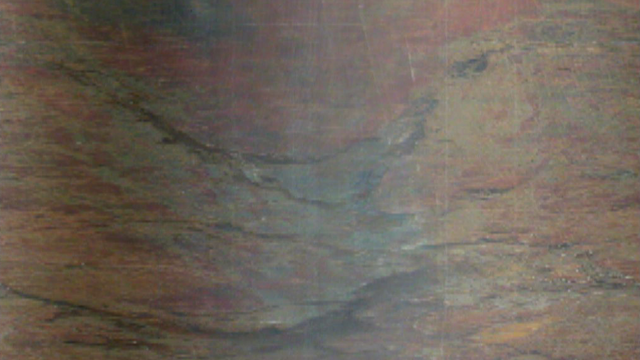

Supplement: S1 File — (ZIP) [file pone.0299471.s001.zip › 0008.png]

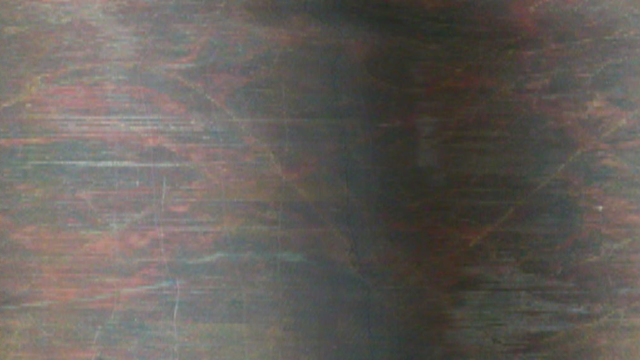

Supplement: S1 File — (ZIP) [file pone.0299471.s001.zip › 0009.png]

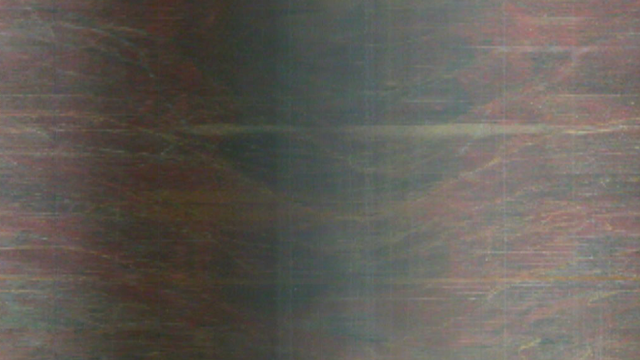

Supplement: S1 File — (ZIP) [file pone.0299471.s001.zip › 0010.png]

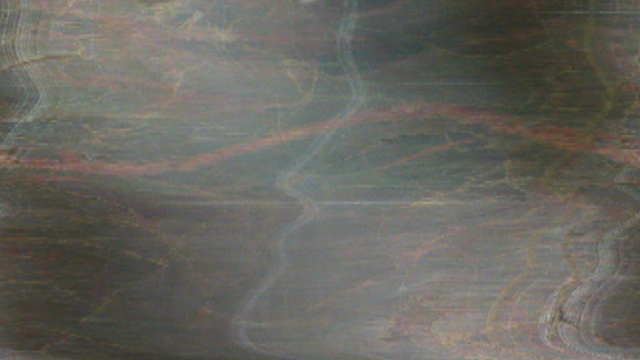

Supplement: S1 File — (ZIP) [file pone.0299471.s001.zip › 0011.png]

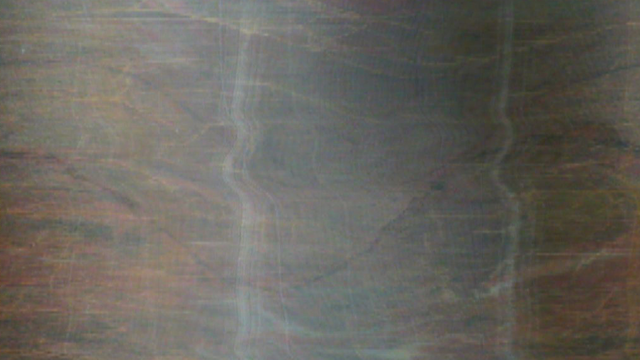

Supplement: S1 File — (ZIP) [file pone.0299471.s001.zip › 0012.png]

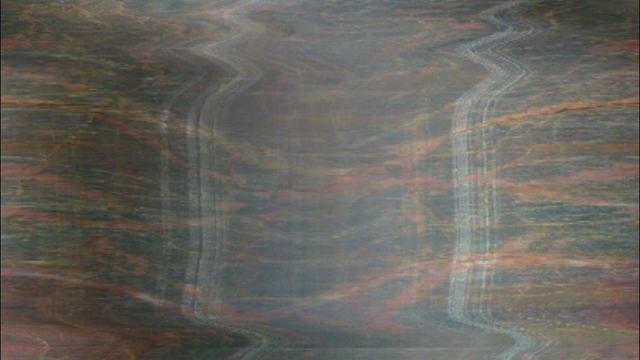

Supplement: S1 File — (ZIP) [file pone.0299471.s001.zip › 0013.png]

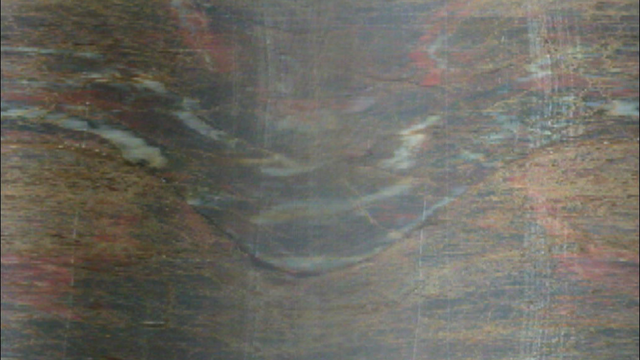

Supplement: S1 File — (ZIP) [file pone.0299471.s001.zip › 0014.png]

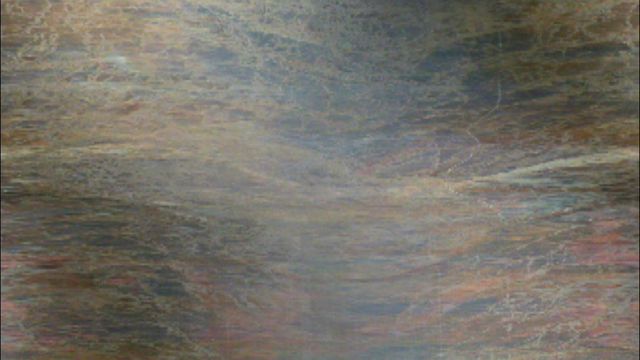

Supplement: S1 File — (ZIP) [file pone.0299471.s001.zip › 0015.png]

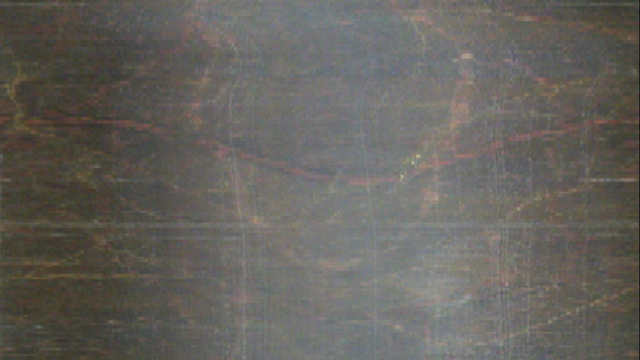

Supplement: S1 File — (ZIP) [file pone.0299471.s001.zip › 0016.png]

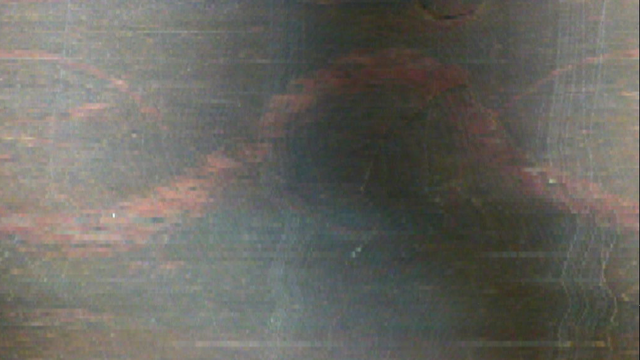

Supplement: S1 File — (ZIP) [file pone.0299471.s001.zip › 0017.png]

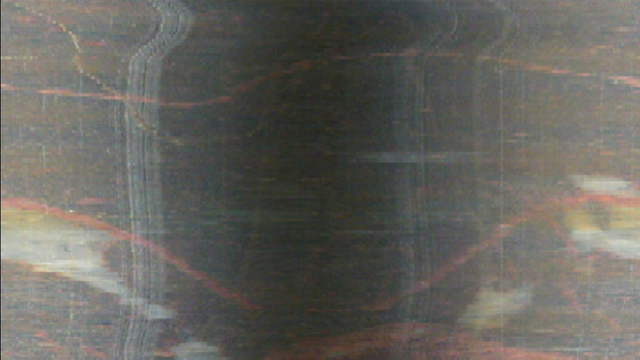

Supplement: S1 File — (ZIP) [file pone.0299471.s001.zip › 0018.png]

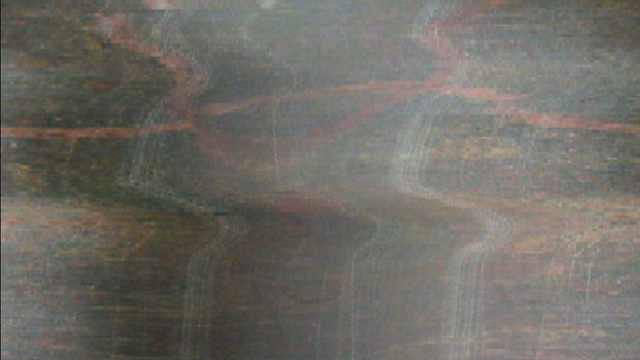

Supplement: S1 File — (ZIP) [file pone.0299471.s001.zip › 0019.png]

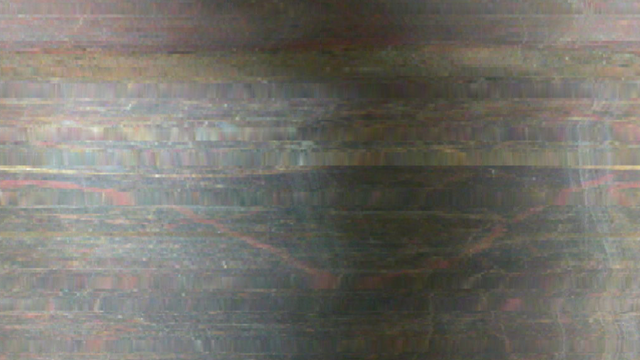

Supplement: S1 File — (ZIP) [file pone.0299471.s001.zip › 0020.png]

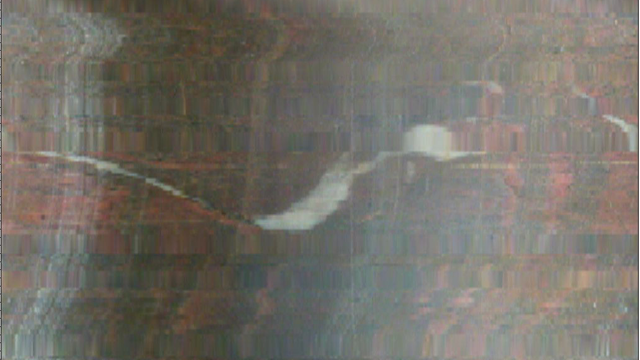

Supplement: S1 File — (ZIP) [file pone.0299471.s001.zip › 0021.png]

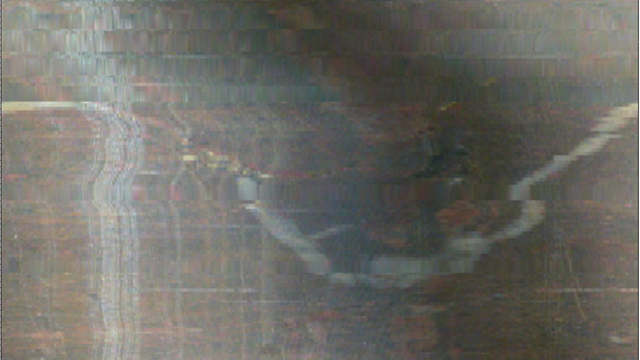

Supplement: S1 File — (ZIP) [file pone.0299471.s001.zip › 0022.png]

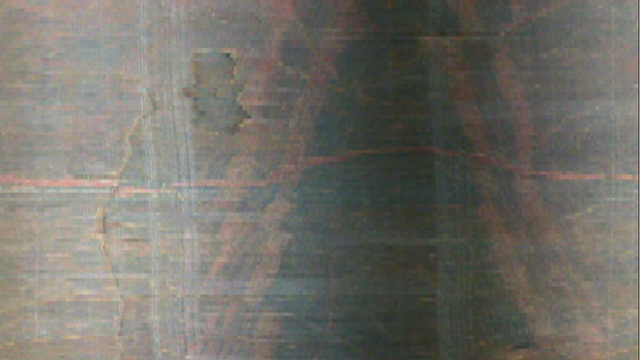

Supplement: S1 File — (ZIP) [file pone.0299471.s001.zip › 0023.png]

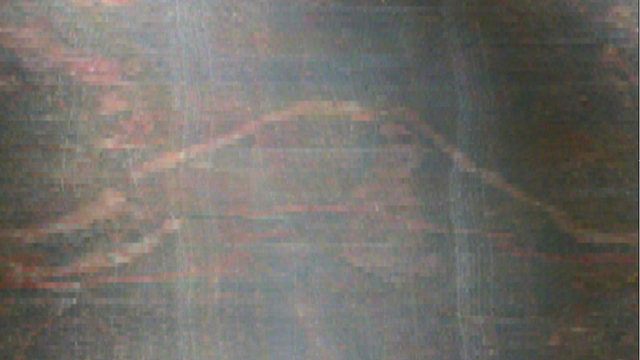

Supplement: S1 File — (ZIP) [file pone.0299471.s001.zip › 0024.png]

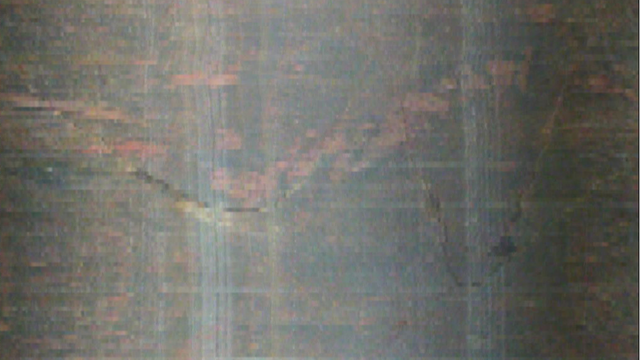

Supplement: S1 File — (ZIP) [file pone.0299471.s001.zip › 0025.png]

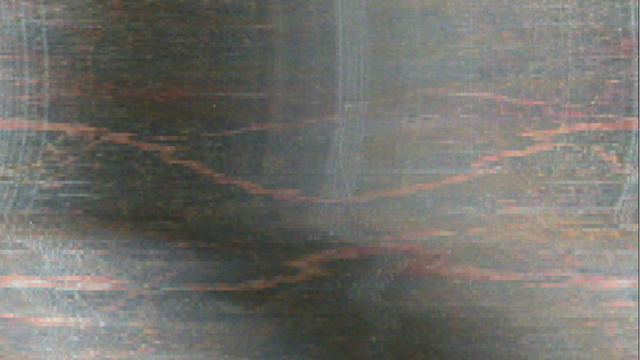

Supplement: S1 File — (ZIP) [file pone.0299471.s001.zip › 0026.png]

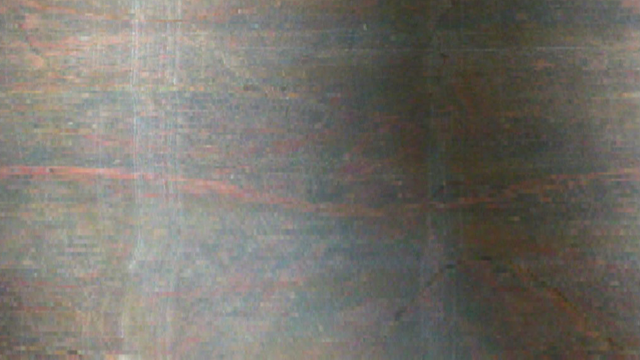

Supplement: S1 File — (ZIP) [file pone.0299471.s001.zip › 0027.png]

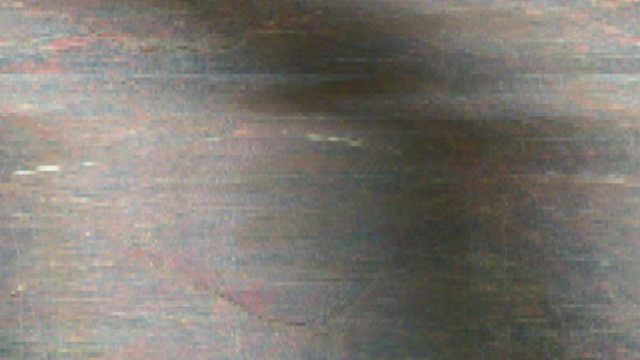

Supplement: S1 File — (ZIP) [file pone.0299471.s001.zip › 0028.png]

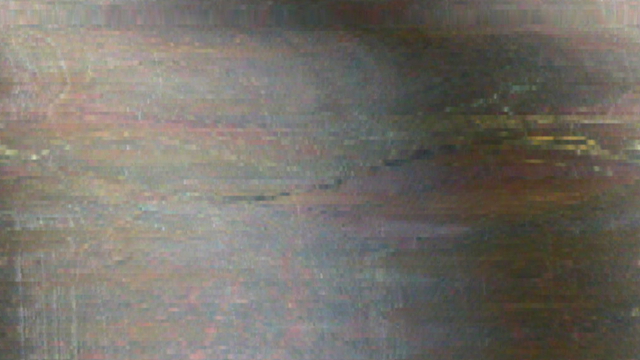

Supplement: S1 File — (ZIP) [file pone.0299471.s001.zip › 0029.png]

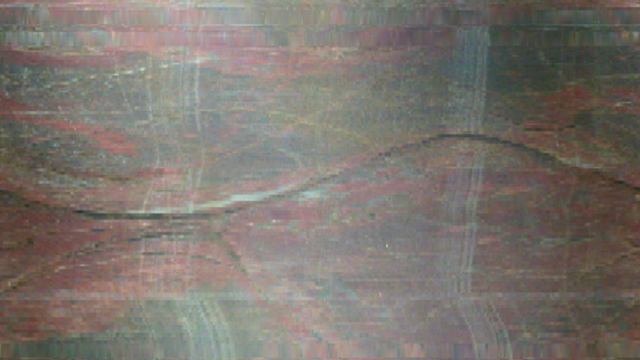

Supplement: S1 File — (ZIP) [file pone.0299471.s001.zip › 0030.png]

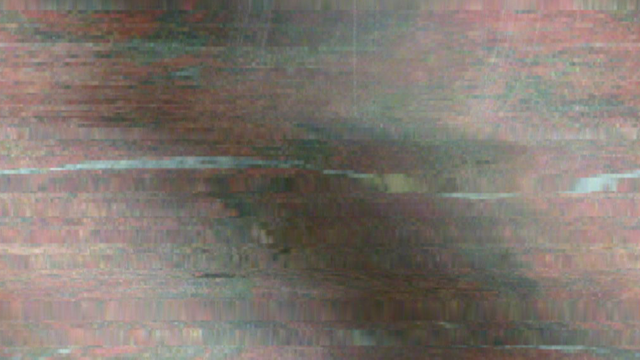

Supplement: S1 File — (ZIP) [file pone.0299471.s001.zip › 0031.png]

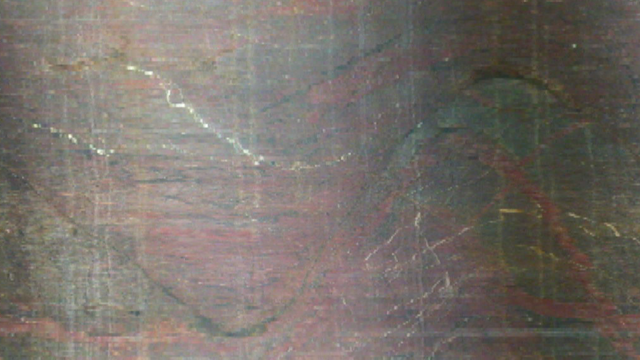

Supplement: S1 File — (ZIP) [file pone.0299471.s001.zip › 0032.png]

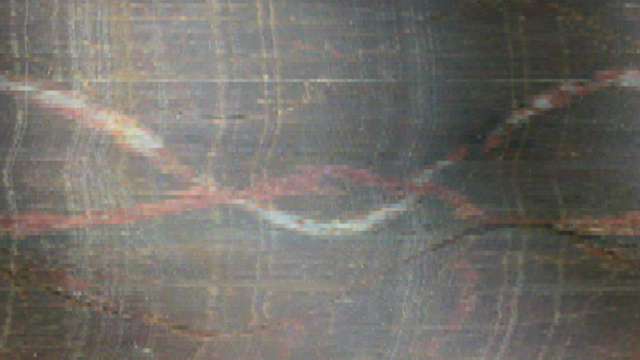

Supplement: S1 File — (ZIP) [file pone.0299471.s001.zip › 0033.png]

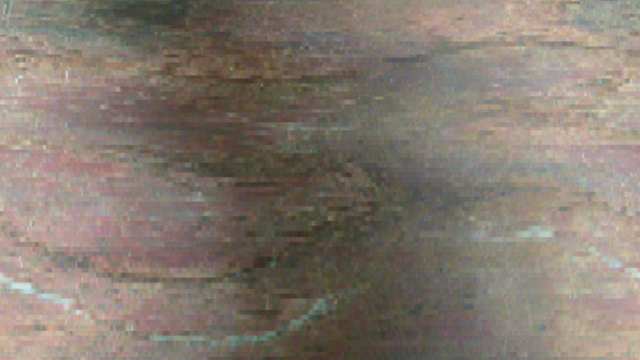

Supplement: S1 File — (ZIP) [file pone.0299471.s001.zip › 0034.png]

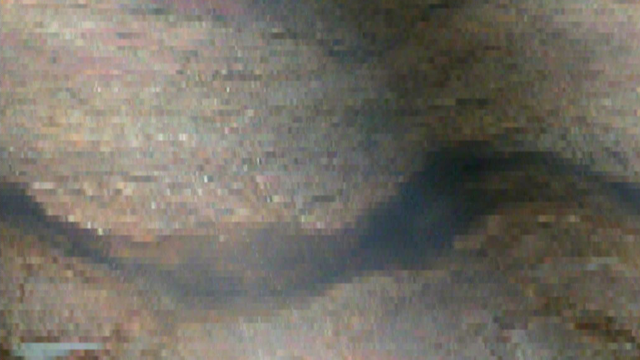

Supplement: S1 File — (ZIP) [file pone.0299471.s001.zip › 0035.png]

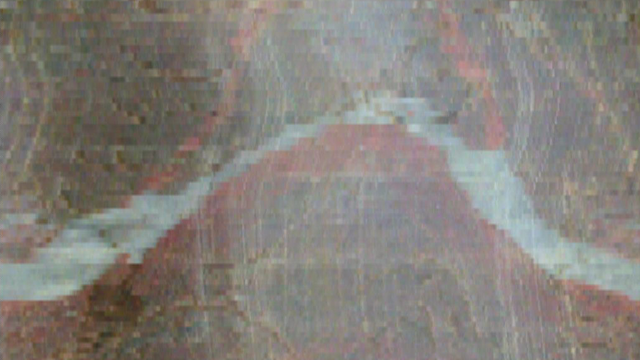

Supplement: S1 File — (ZIP) [file pone.0299471.s001.zip › 0036.png]

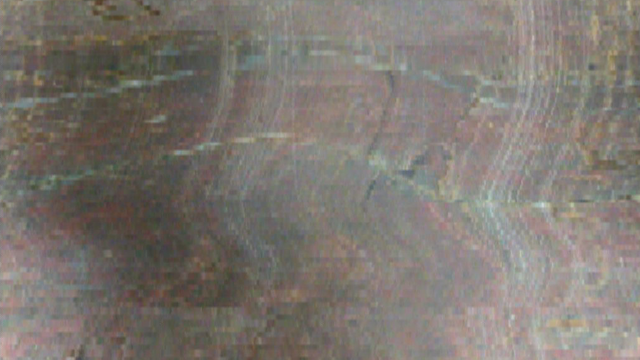

Supplement: S1 File — (ZIP) [file pone.0299471.s001.zip › 0037.png]

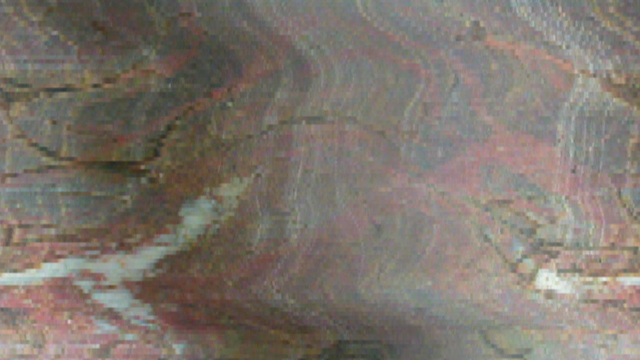

Supplement: S1 File — (ZIP) [file pone.0299471.s001.zip › 0038.png]

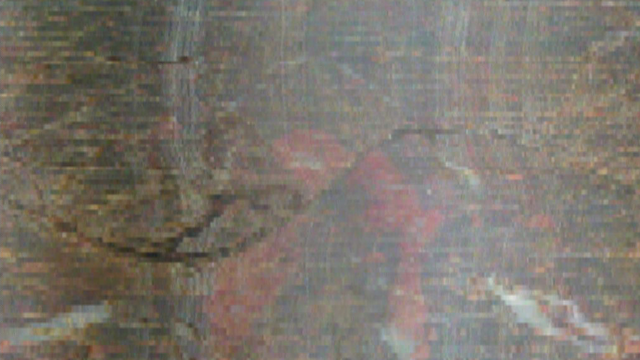

Supplement: S1 File — (ZIP) [file pone.0299471.s001.zip › 0039.png]

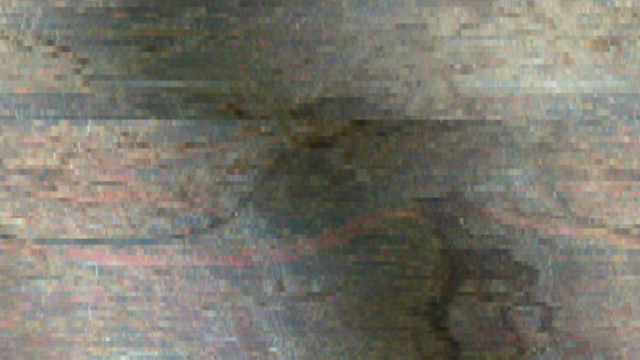

Supplement: S1 File — (ZIP) [file pone.0299471.s001.zip › 0040.png]

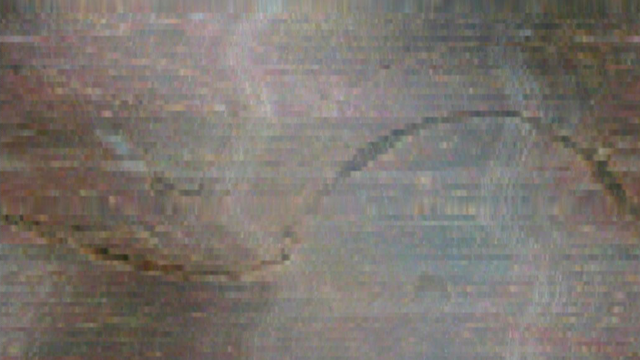

Supplement: S1 File — (ZIP) [file pone.0299471.s001.zip › 0041.png]

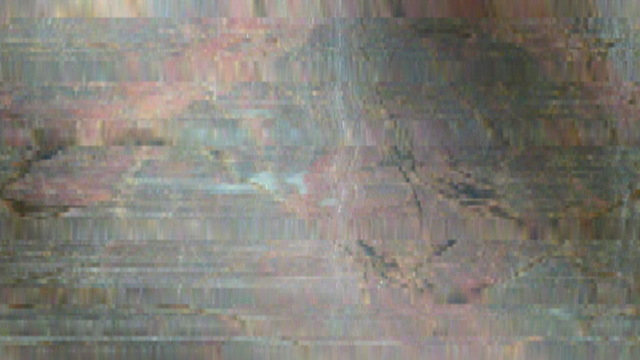

Supplement: S1 File — (ZIP) [file pone.0299471.s001.zip › 0042.png]

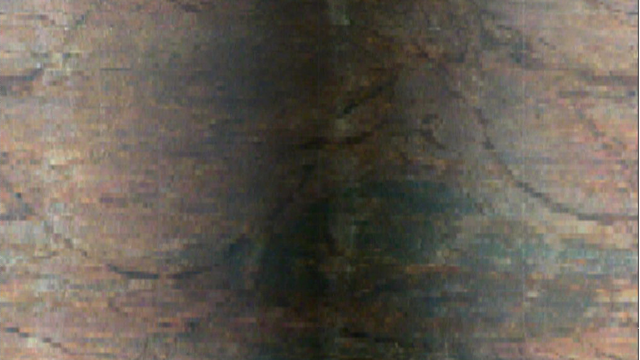

Supplement: S1 File — (ZIP) [file pone.0299471.s001.zip › 0043.png]

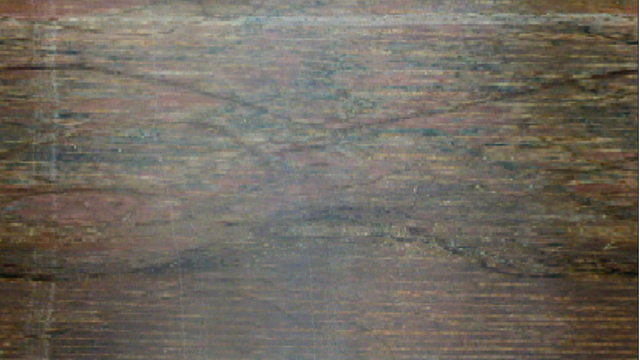

Supplement: S1 File — (ZIP) [file pone.0299471.s001.zip › 0044.png]

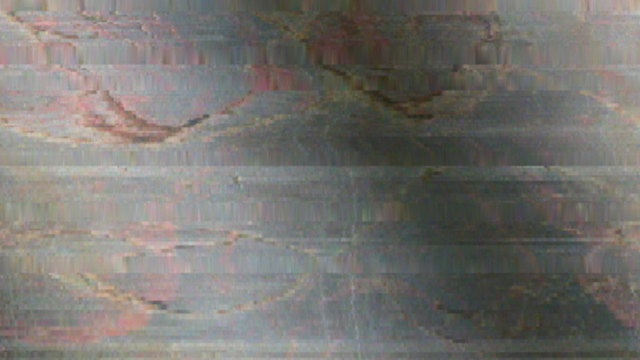

Supplement: S1 File — (ZIP) [file pone.0299471.s001.zip › 0045.png]

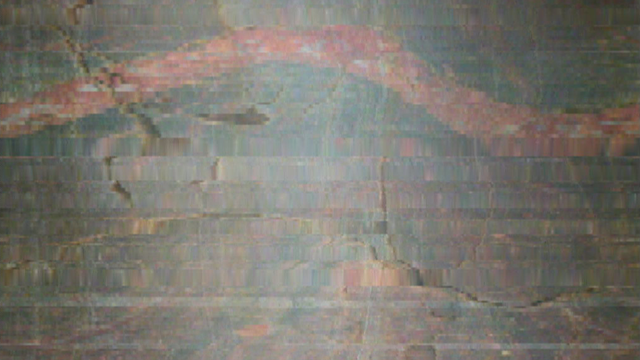

Supplement: S1 File — (ZIP) [file pone.0299471.s001.zip › 0046.png]

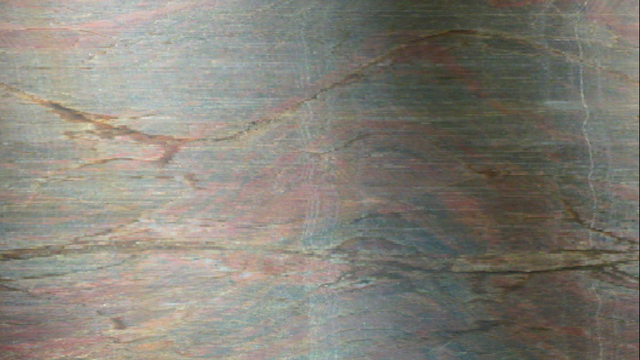

Supplement: S1 File — (ZIP) [file pone.0299471.s001.zip › 0047.png]

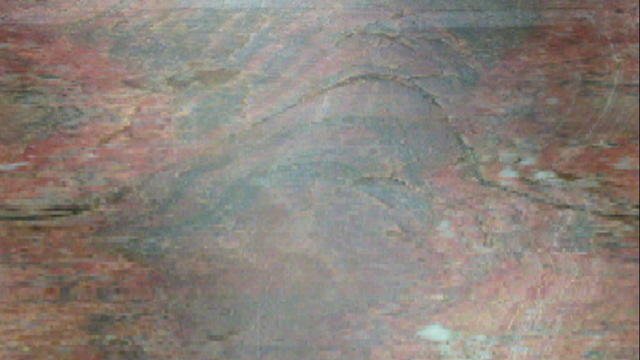

Supplement: S1 File — (ZIP) [file pone.0299471.s001.zip › 0048.png]

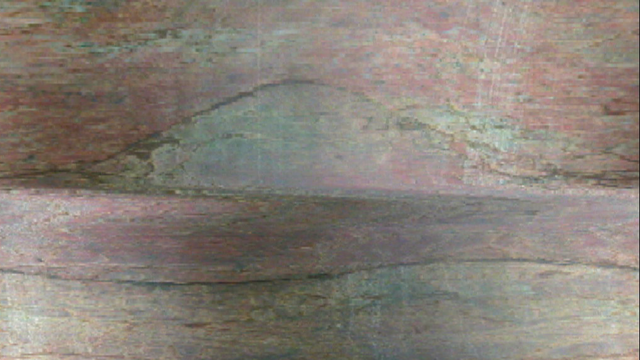

Supplement: S1 File — (ZIP) [file pone.0299471.s001.zip › 0049.png]

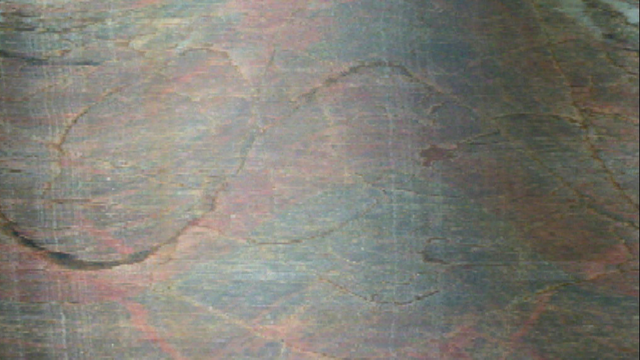

Supplement: S1 File — (ZIP) [file pone.0299471.s001.zip › 0050.png]

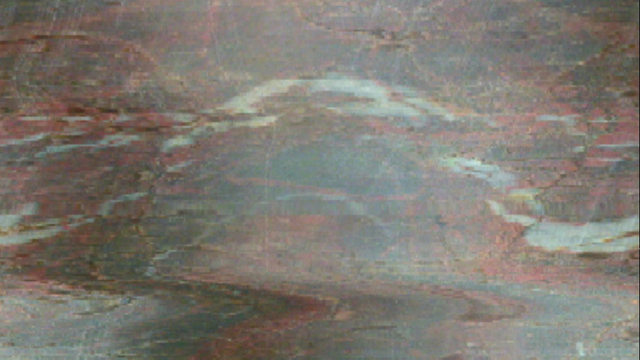

Supplement: S1 File — (ZIP) [file pone.0299471.s001.zip › 0051.png]

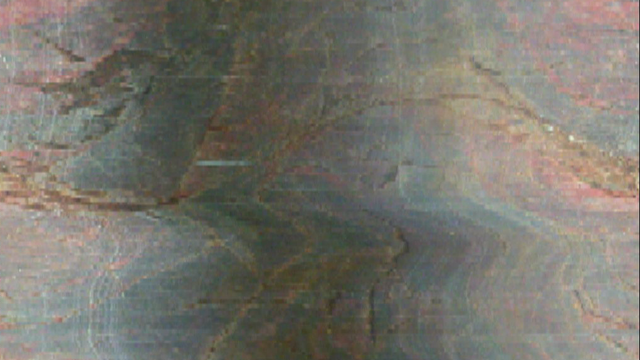

Supplement: S1 File — (ZIP) [file pone.0299471.s001.zip › 0052.png]

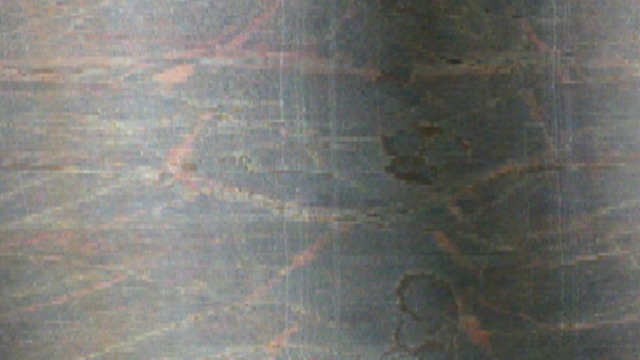

Supplement: S1 File — (ZIP) [file pone.0299471.s001.zip › 0053.png]

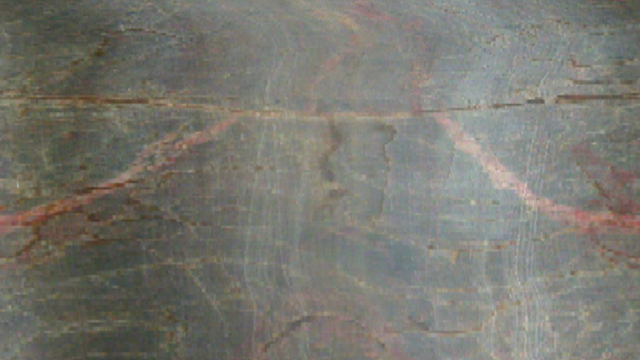

Supplement: S1 File — (ZIP) [file pone.0299471.s001.zip › 0054.png]

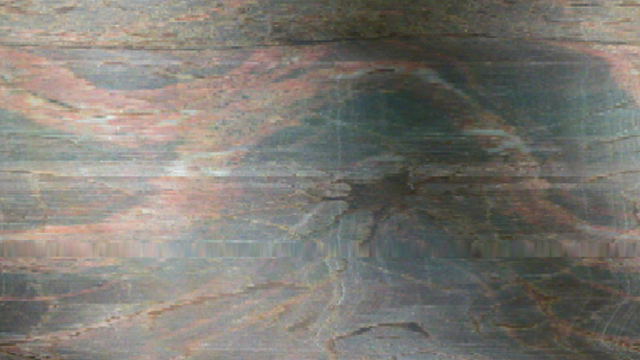

Supplement: S1 File — (ZIP) [file pone.0299471.s001.zip › 0055.png]

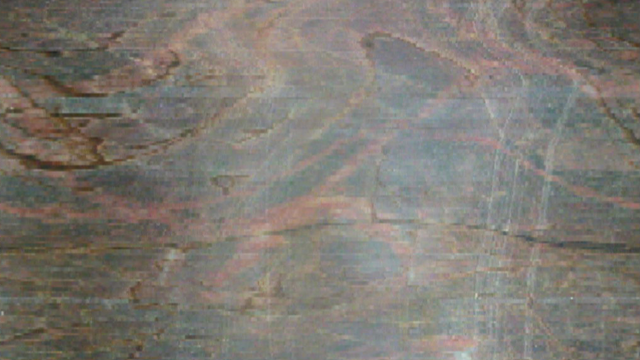

Supplement: S1 File — (ZIP) [file pone.0299471.s001.zip › 0056.png]

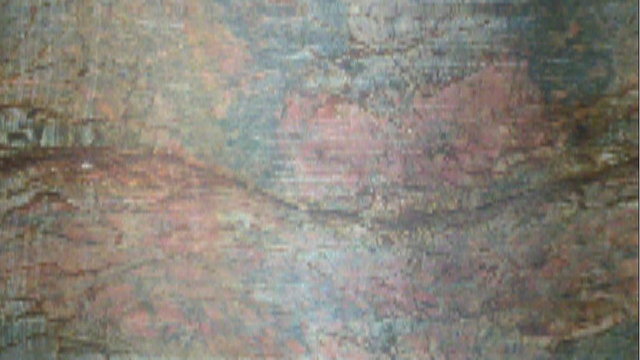

Supplement: S1 File — (ZIP) [file pone.0299471.s001.zip › 0057.png]

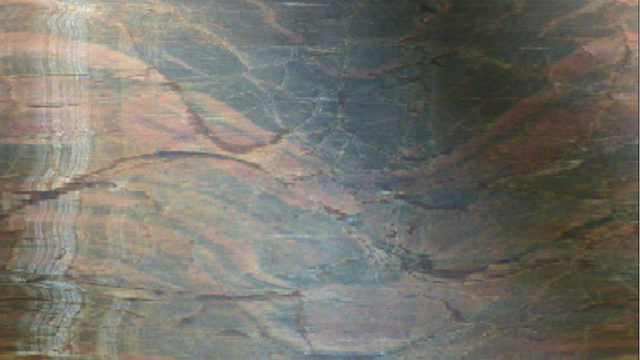

Supplement: S1 File — (ZIP) [file pone.0299471.s001.zip › 0058.png]

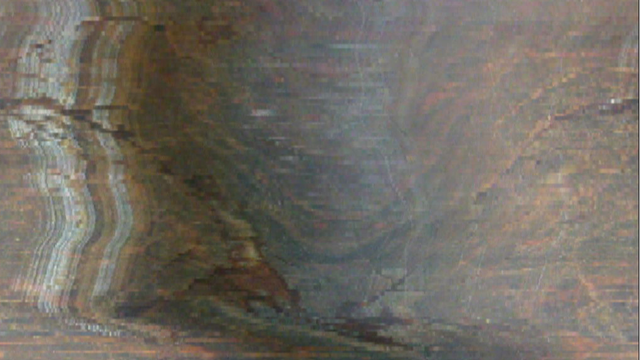

Supplement: S1 File — (ZIP) [file pone.0299471.s001.zip › 0059.png]

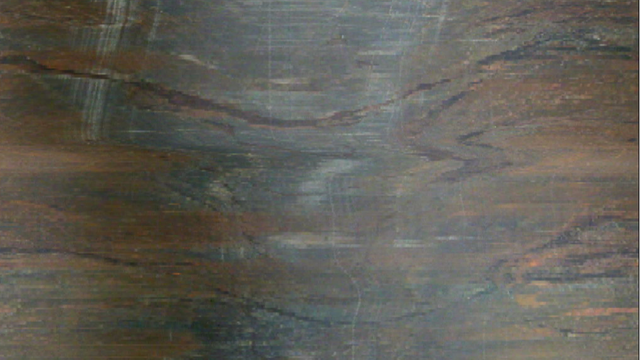

Supplement: S1 File — (ZIP) [file pone.0299471.s001.zip › 0060.png]

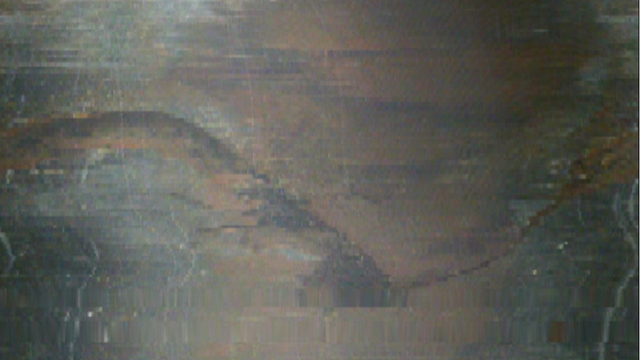

Supplement: S1 File — (ZIP) [file pone.0299471.s001.zip › 0061.png]

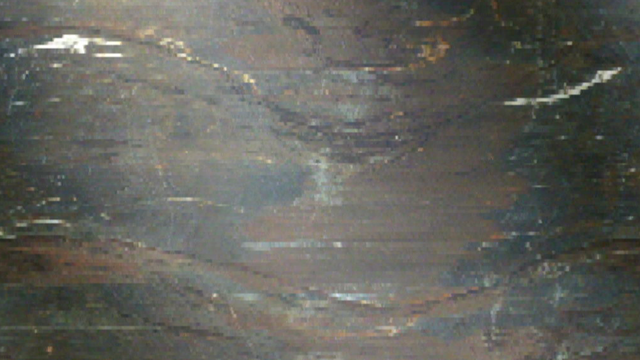

Supplement: S1 File — (ZIP) [file pone.0299471.s001.zip › 0062.png]

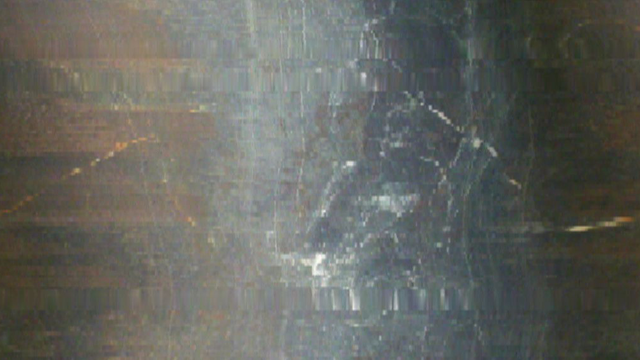

Supplement: S1 File — (ZIP) [file pone.0299471.s001.zip › 0063.png]

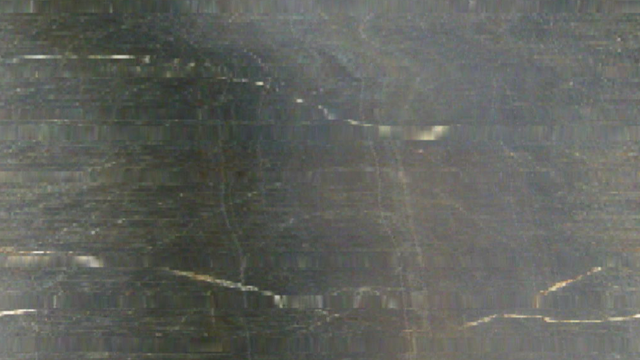

Supplement: S1 File — (ZIP) [file pone.0299471.s001.zip › 0064.png]

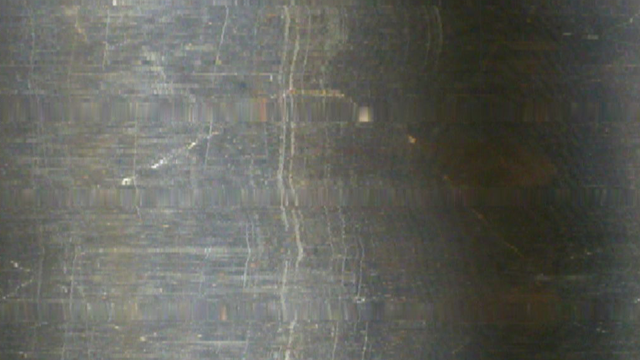

Supplement: S1 File — (ZIP) [file pone.0299471.s001.zip › 0065.png]

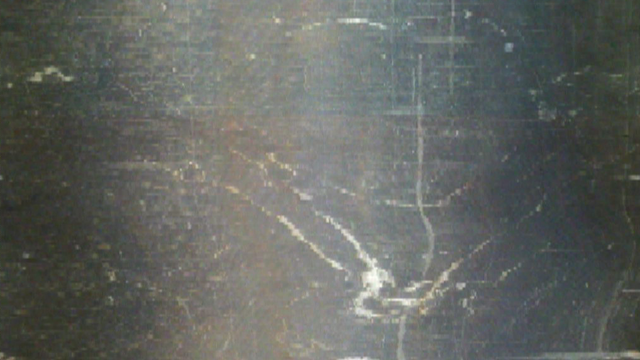

Supplement: S1 File — (ZIP) [file pone.0299471.s001.zip › 0066.png]

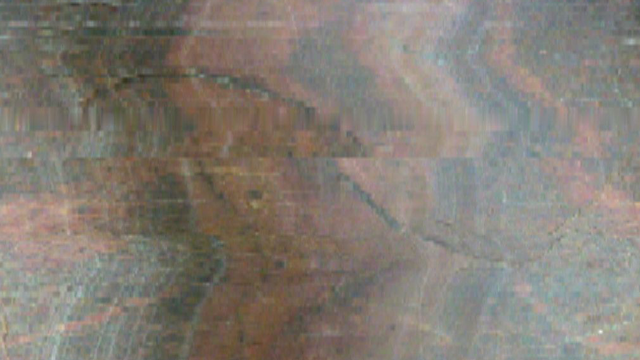

Supplement: S1 File — (ZIP) [file pone.0299471.s001.zip › 0067.png]

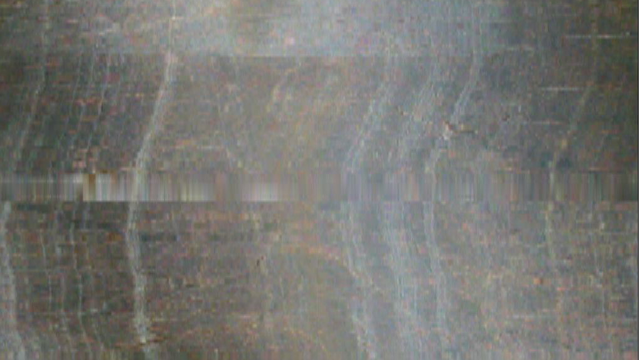

Supplement: S1 File — (ZIP) [file pone.0299471.s001.zip › 0068.png]

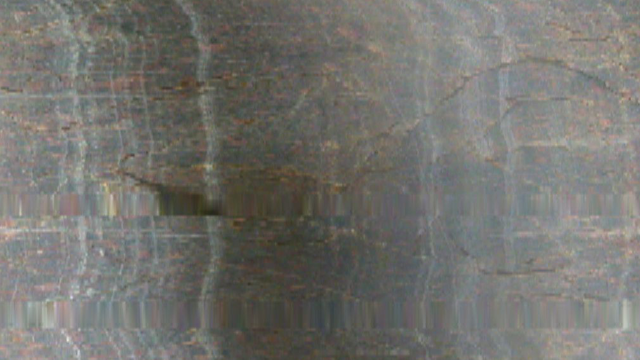

Supplement: S1 File — (ZIP) [file pone.0299471.s001.zip › 0069.png]

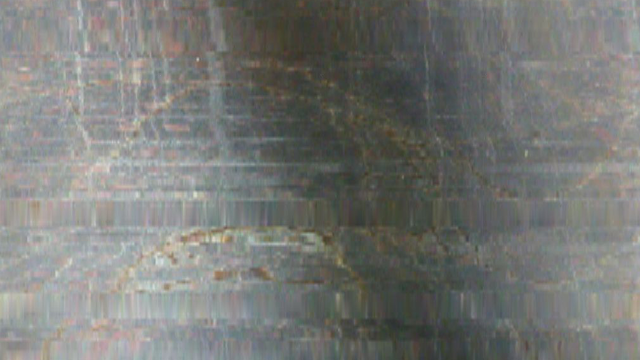

Supplement: S1 File — (ZIP) [file pone.0299471.s001.zip › 0070.png]

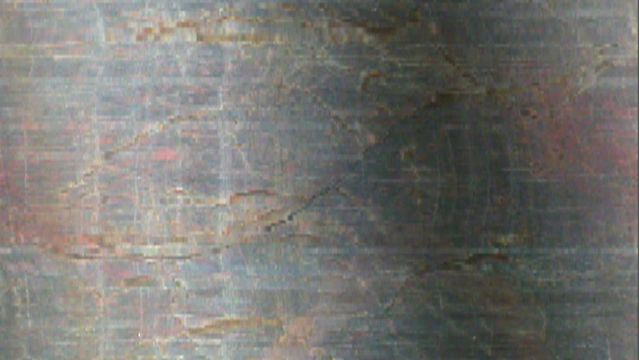

Supplement: S1 File — (ZIP) [file pone.0299471.s001.zip › 0071.png]

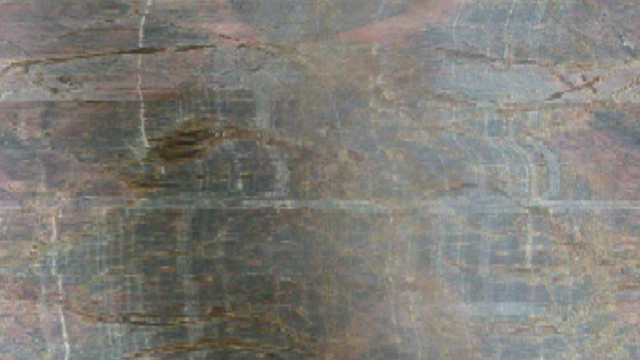

Supplement: S1 File — (ZIP) [file pone.0299471.s001.zip › 0072.png]

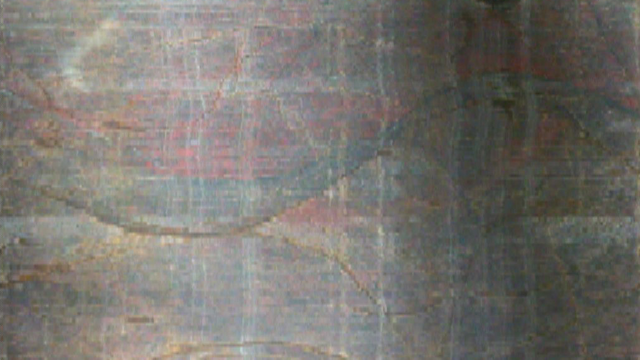

Supplement: S1 File — (ZIP) [file pone.0299471.s001.zip › 0073.png]

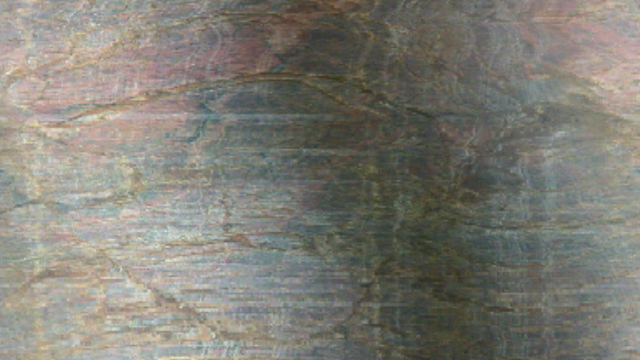

Supplement: S1 File — (ZIP) [file pone.0299471.s001.zip › 0074.png]

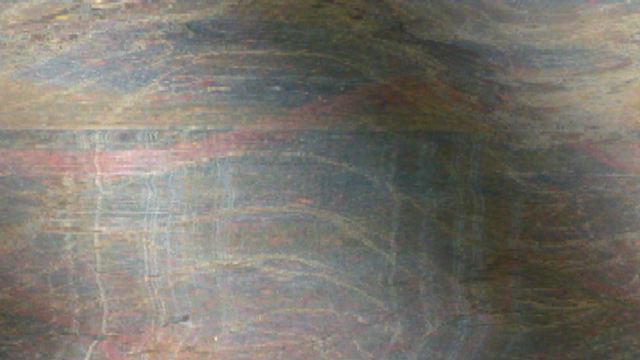

Supplement: S1 File — (ZIP) [file pone.0299471.s001.zip › 0075.png]

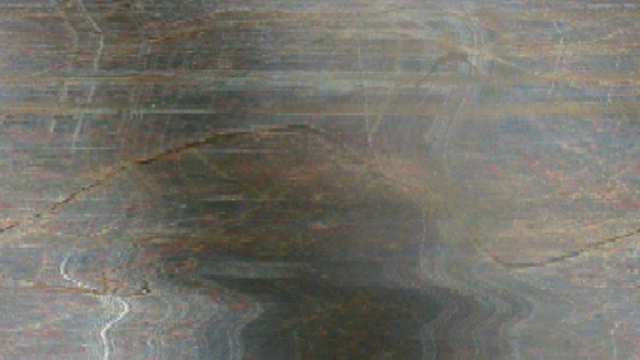

Supplement: S1 File — (ZIP) [file pone.0299471.s001.zip › 0076.png]

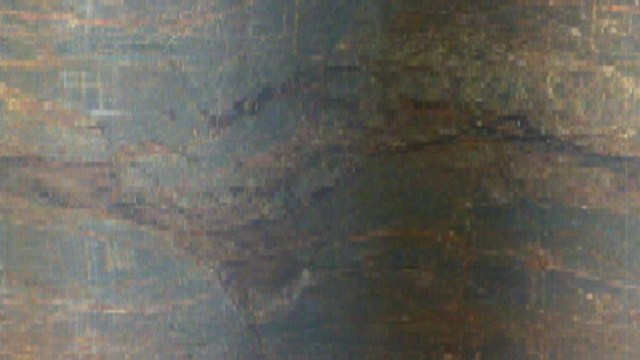

Supplement: S1 File — (ZIP) [file pone.0299471.s001.zip › 0077.png]

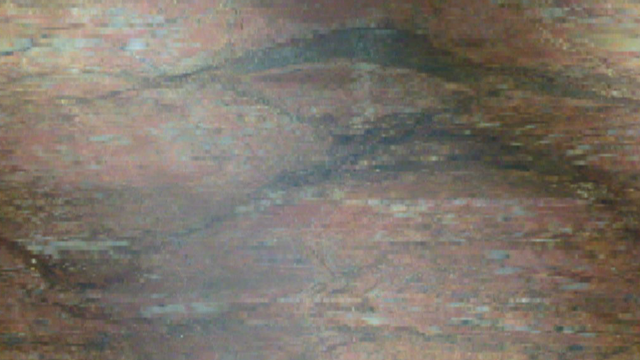

Supplement: S1 File — (ZIP) [file pone.0299471.s001.zip › 0078.png]

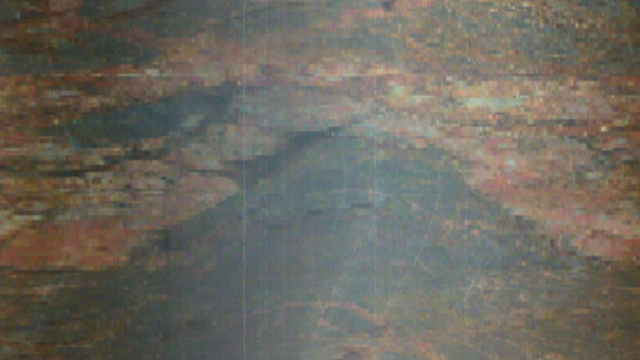

Supplement: S1 File — (ZIP) [file pone.0299471.s001.zip › 0079.png]

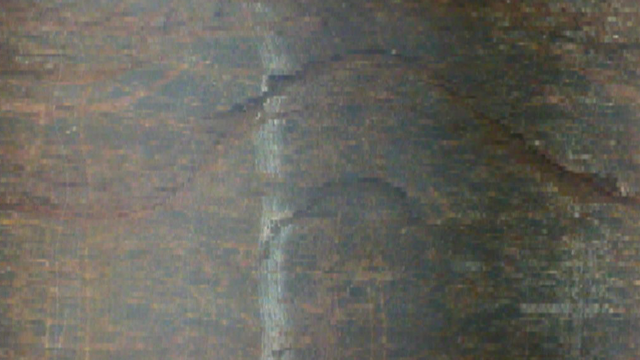

Supplement: S1 File — (ZIP) [file pone.0299471.s001.zip › 0080.png]

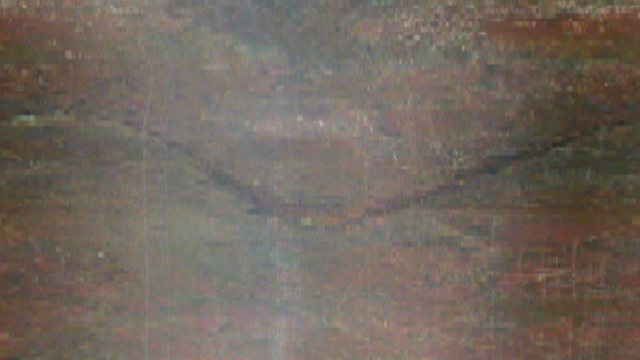

Supplement: S1 File — (ZIP) [file pone.0299471.s001.zip › 0081.png]

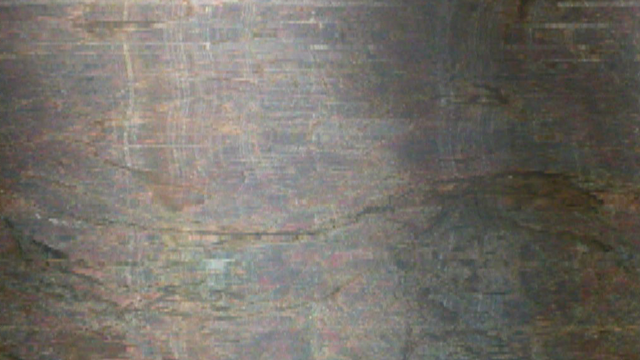

Supplement: S1 File — (ZIP) [file pone.0299471.s001.zip › 0082.png]

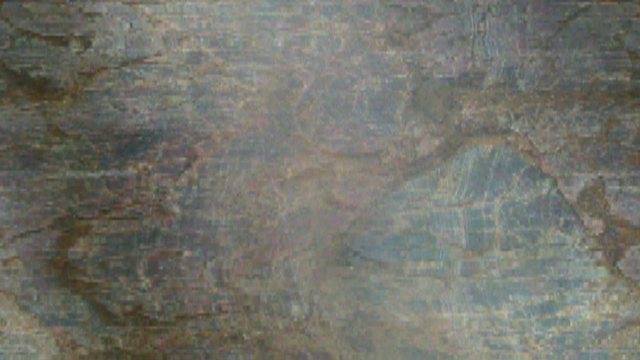

Supplement: S1 File — (ZIP) [file pone.0299471.s001.zip › 0083.png]

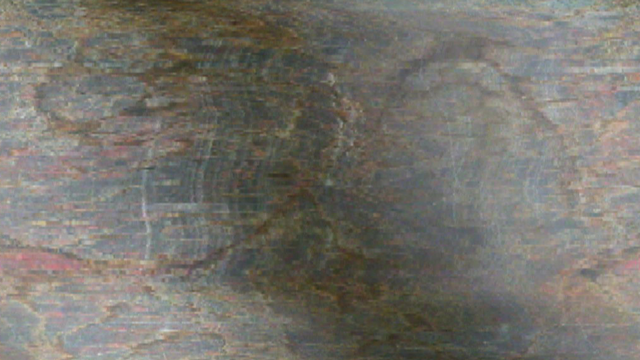

Supplement: S1 File — (ZIP) [file pone.0299471.s001.zip › 0084.png]

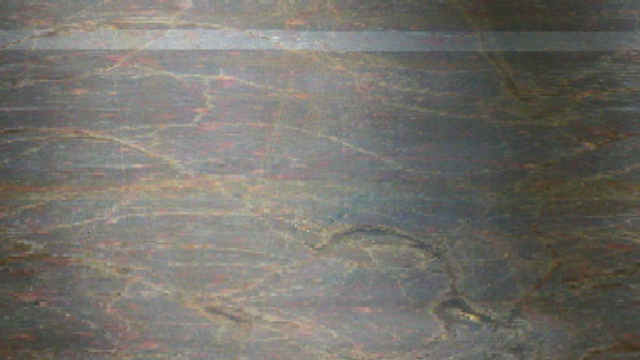

Supplement: S1 File — (ZIP) [file pone.0299471.s001.zip › 0085.png]

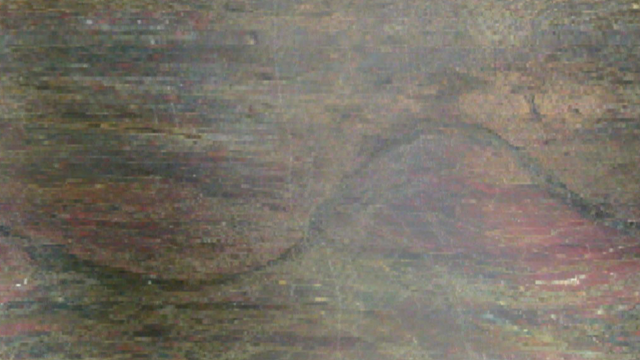

Supplement: S1 File — (ZIP) [file pone.0299471.s001.zip › 0086.png]

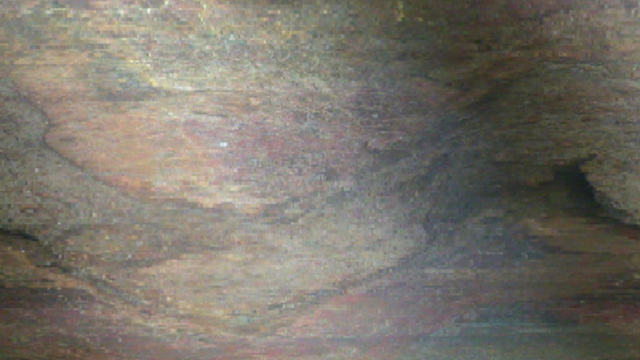

Supplement: S1 File — (ZIP) [file pone.0299471.s001.zip › 0087.png]

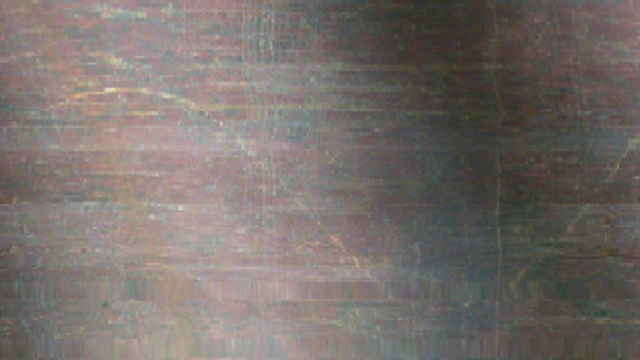

Supplement: S1 File — (ZIP) [file pone.0299471.s001.zip › 0088.png]

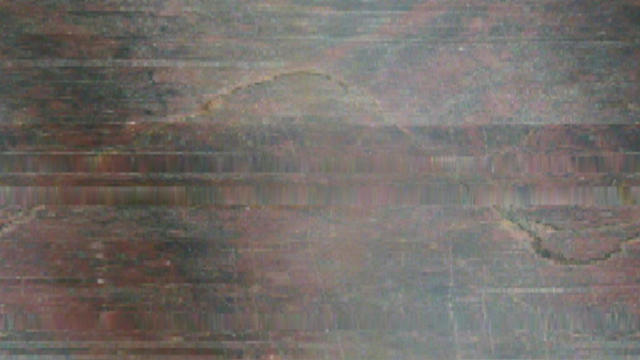

Supplement: S1 File — (ZIP) [file pone.0299471.s001.zip › 0089.png]

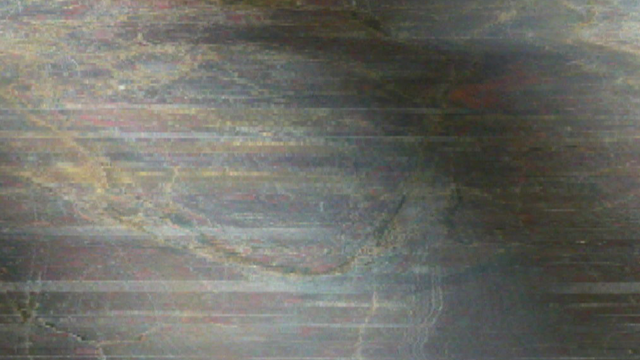

Supplement: S1 File — (ZIP) [file pone.0299471.s001.zip › 0090.png]

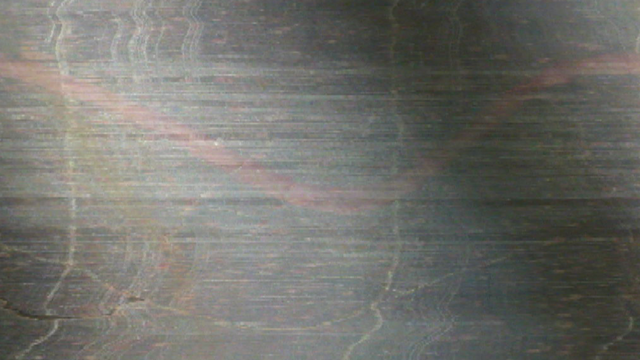

Supplement: S1 File — (ZIP) [file pone.0299471.s001.zip › 0091.png]

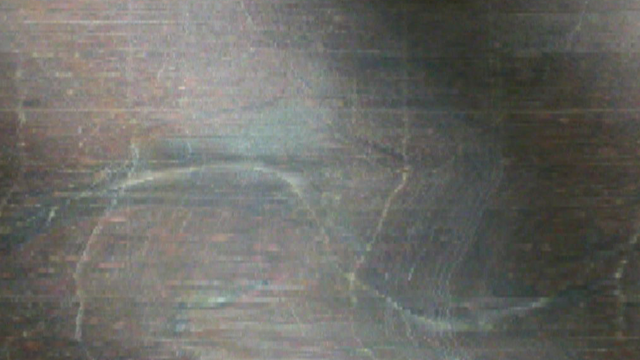

Supplement: S1 File — (ZIP) [file pone.0299471.s001.zip › 0092.png]

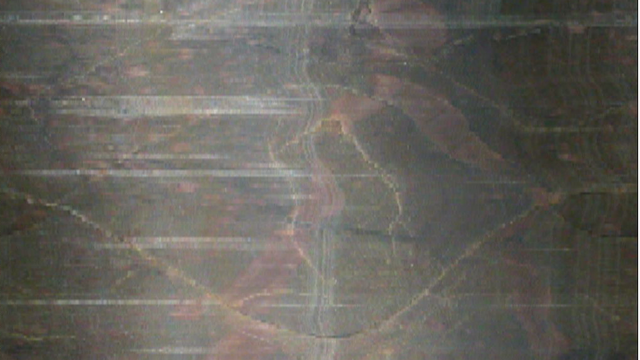

Supplement: S1 File — (ZIP) [file pone.0299471.s001.zip › 0093.png]

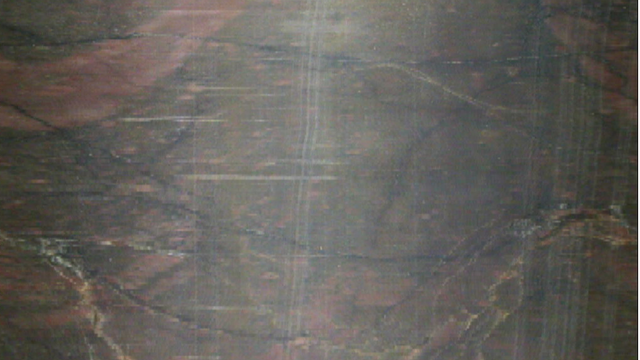

Supplement: S1 File — (ZIP) [file pone.0299471.s001.zip › 0094.png]

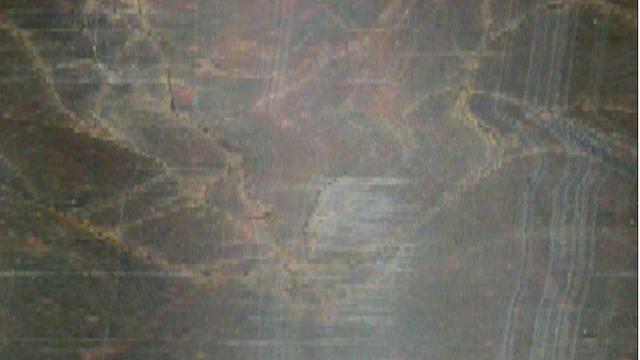

Supplement: S1 File — (ZIP) [file pone.0299471.s001.zip › 0095.png]

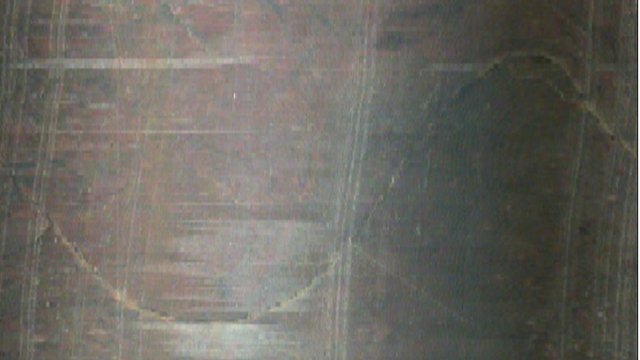

Supplement: S1 File — (ZIP) [file pone.0299471.s001.zip › 0096.png]

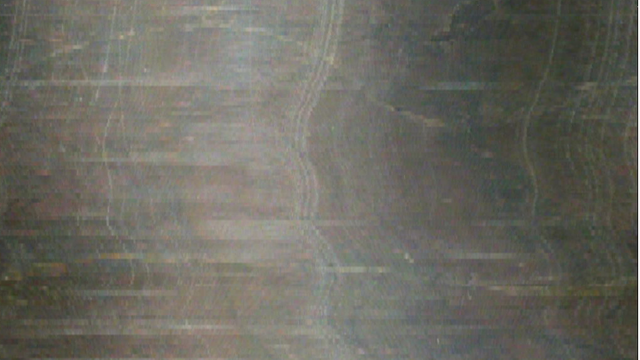

Supplement: S1 File — (ZIP) [file pone.0299471.s001.zip › 0097.png]

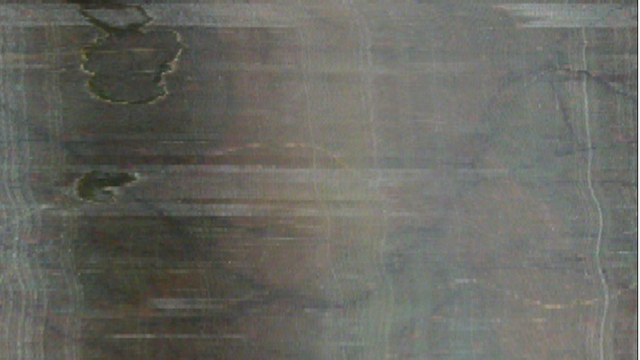

Supplement: S1 File — (ZIP) [file pone.0299471.s001.zip › 0098.png]

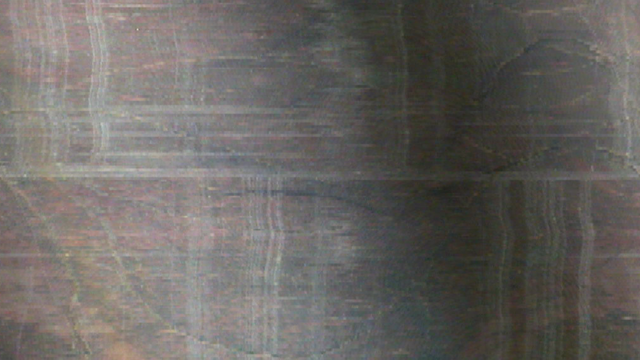

Supplement: S1 File — (ZIP) [file pone.0299471.s001.zip › 0099.png]
