# Supplementary figures and images for: The segmentation and intelligent recognition of structural surfaces in borehole images based on the U2-Net network (part 4 of 4)
Source: PLoS One. 2024 Mar 7;19(3):e0299471. doi: 10.1371/journal.pone.0299471 (PMC10919631; doi:10.1371/journal.pone.0299471)

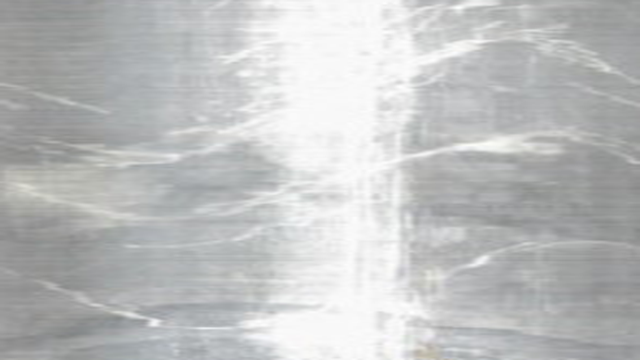

Supplement: S2 File — (ZIP) [file pone.0299471.s002.zip › 0300.png]

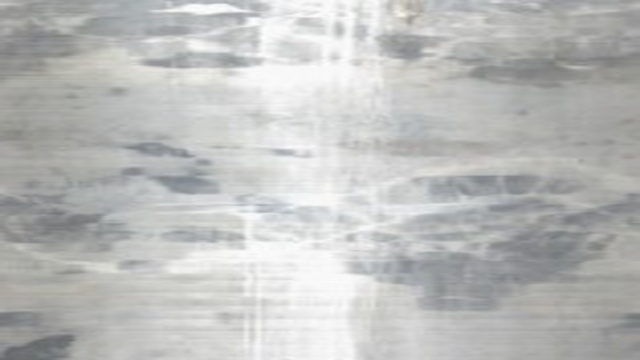

Supplement: S2 File — (ZIP) [file pone.0299471.s002.zip › 0301.png]

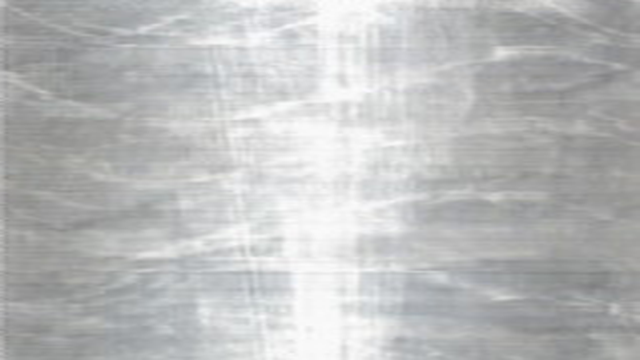

Supplement: S2 File — (ZIP) [file pone.0299471.s002.zip › 0302.png]

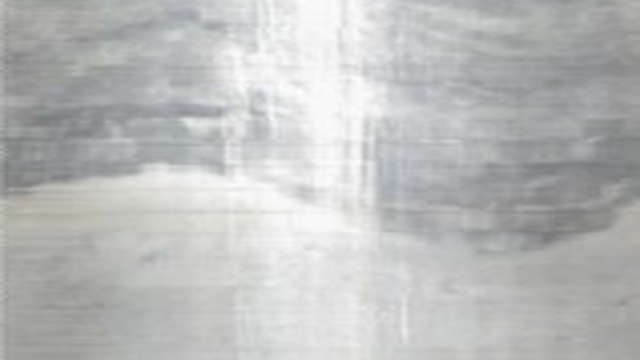

Supplement: S2 File — (ZIP) [file pone.0299471.s002.zip › 0303.png]

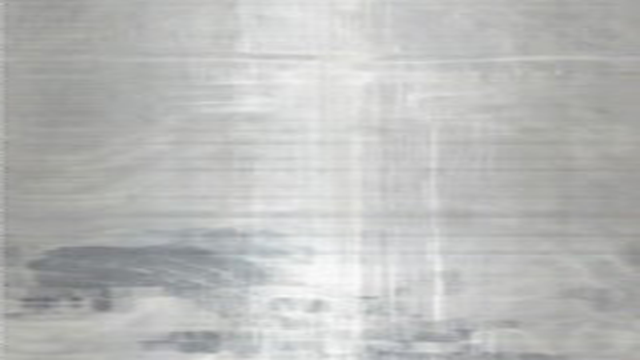

Supplement: S2 File — (ZIP) [file pone.0299471.s002.zip › 0304.png]

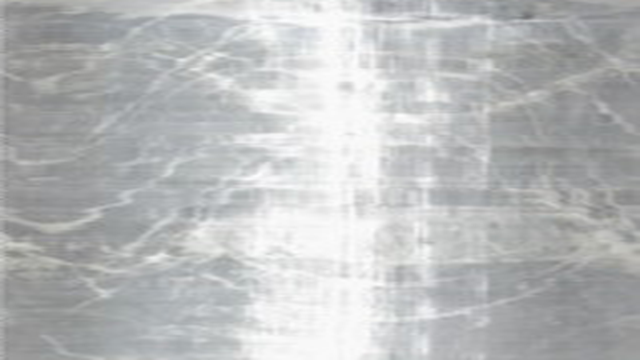

Supplement: S2 File — (ZIP) [file pone.0299471.s002.zip › 0305.png]

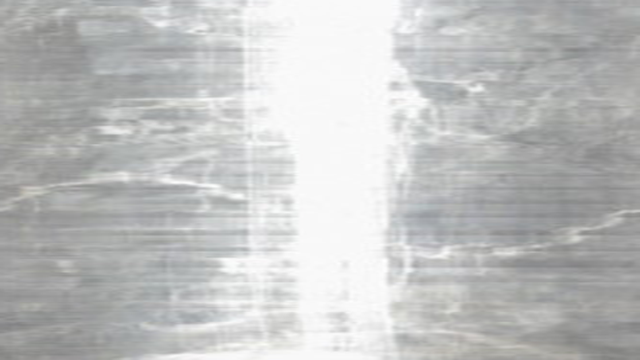

Supplement: S2 File — (ZIP) [file pone.0299471.s002.zip › 0306.png]

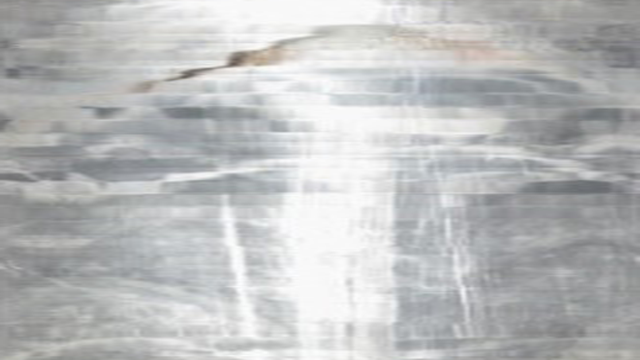

Supplement: S2 File — (ZIP) [file pone.0299471.s002.zip › 0307.png]

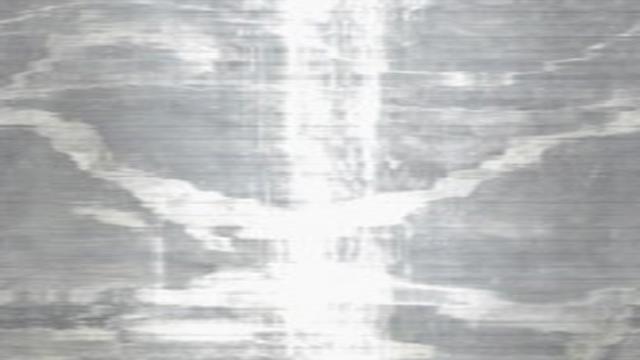

Supplement: S2 File — (ZIP) [file pone.0299471.s002.zip › 0308.jpg]

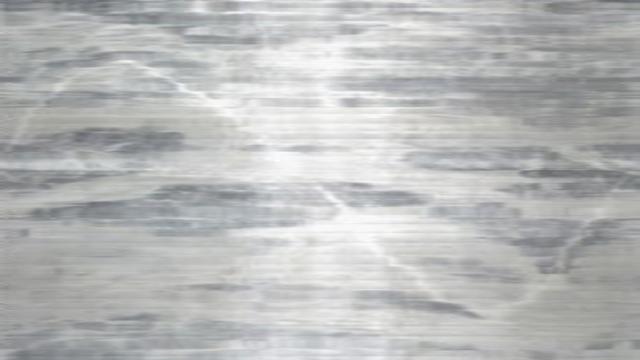

Supplement: S2 File — (ZIP) [file pone.0299471.s002.zip › 0309.jpg]

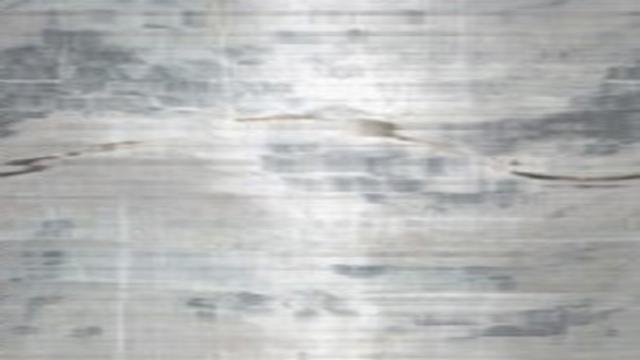

Supplement: S2 File — (ZIP) [file pone.0299471.s002.zip › 0310.jpg]

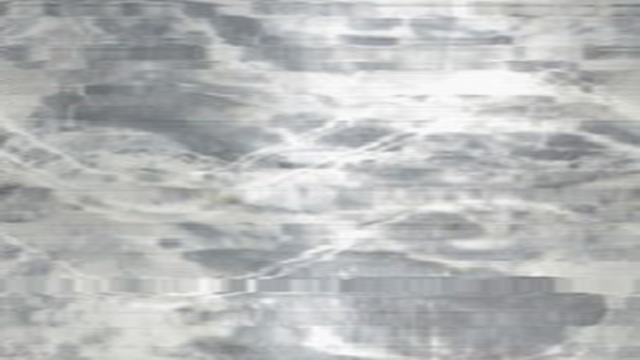

Supplement: S2 File — (ZIP) [file pone.0299471.s002.zip › 0311.png]

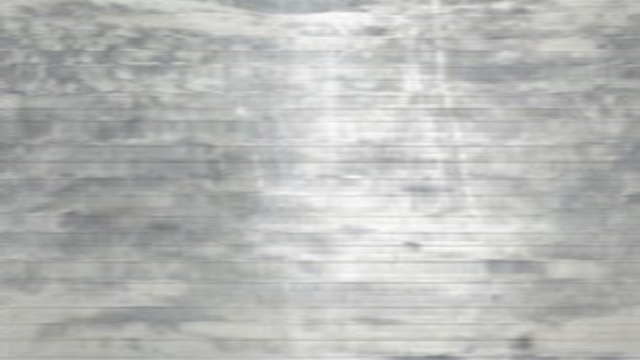

Supplement: S2 File — (ZIP) [file pone.0299471.s002.zip › 0312.png]

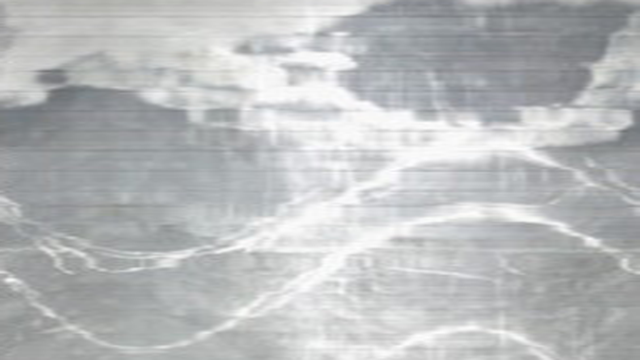

Supplement: S2 File — (ZIP) [file pone.0299471.s002.zip › 0313.png]

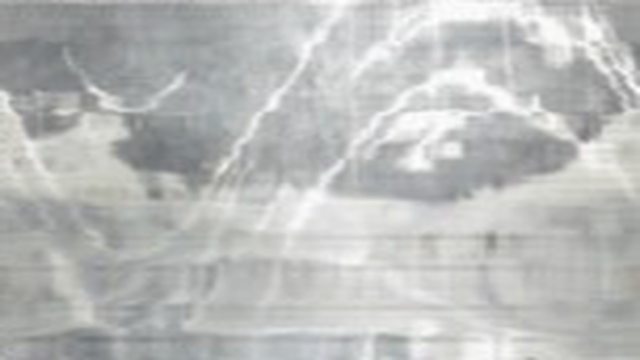

Supplement: S2 File — (ZIP) [file pone.0299471.s002.zip › 0314.png]

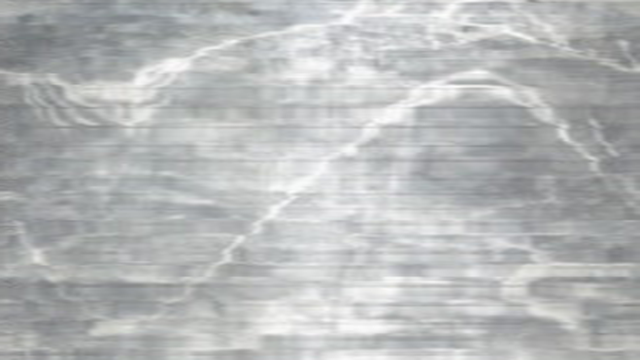

Supplement: S2 File — (ZIP) [file pone.0299471.s002.zip › 0315.png]

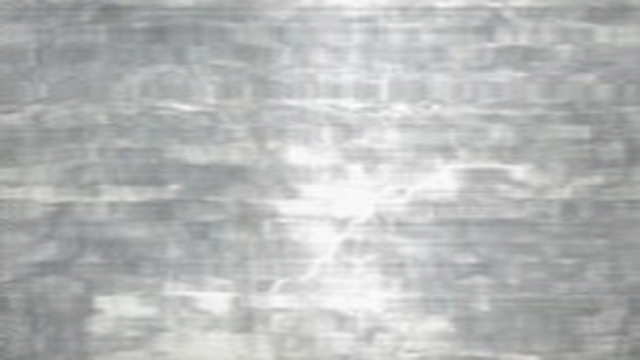

Supplement: S2 File — (ZIP) [file pone.0299471.s002.zip › 0316.png]

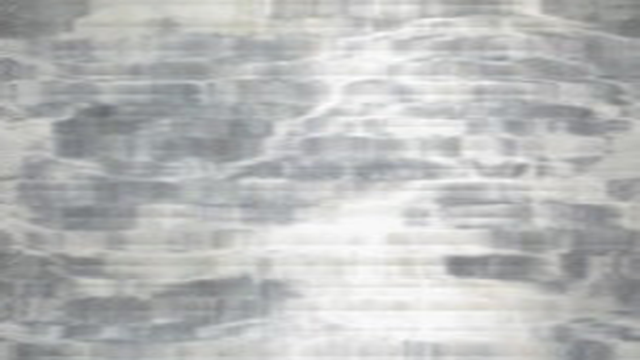

Supplement: S2 File — (ZIP) [file pone.0299471.s002.zip › 0317.png]

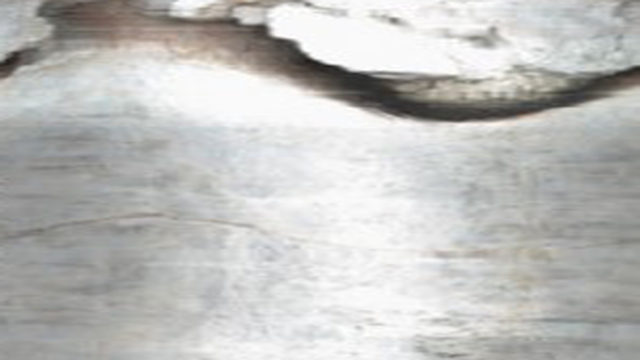

Supplement: S2 File — (ZIP) [file pone.0299471.s002.zip › 0318.png]

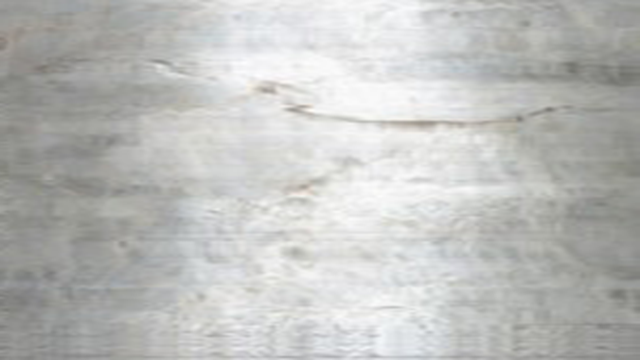

Supplement: S2 File — (ZIP) [file pone.0299471.s002.zip › 0319.png]

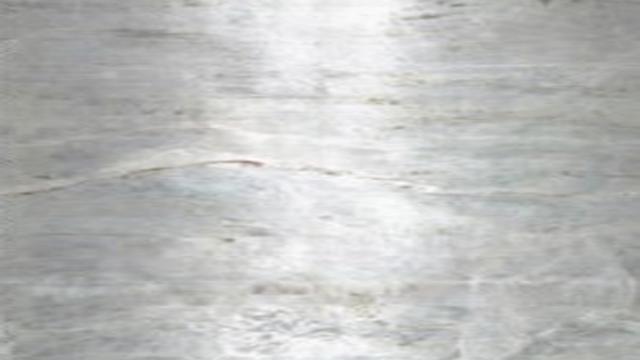

Supplement: S2 File — (ZIP) [file pone.0299471.s002.zip › 0320.jpg]

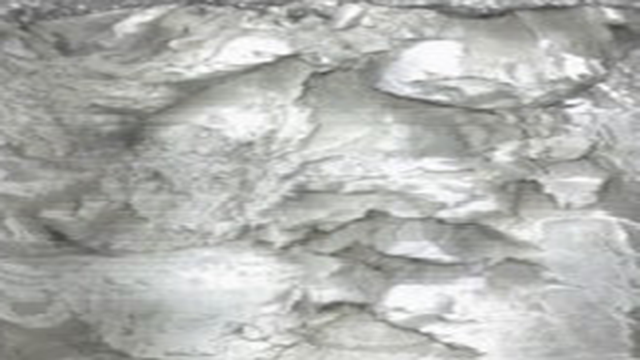

Supplement: S2 File — (ZIP) [file pone.0299471.s002.zip › 0321.png]

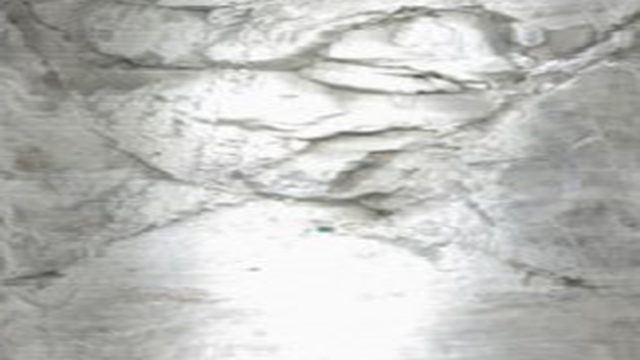

Supplement: S2 File — (ZIP) [file pone.0299471.s002.zip › 0322.png]

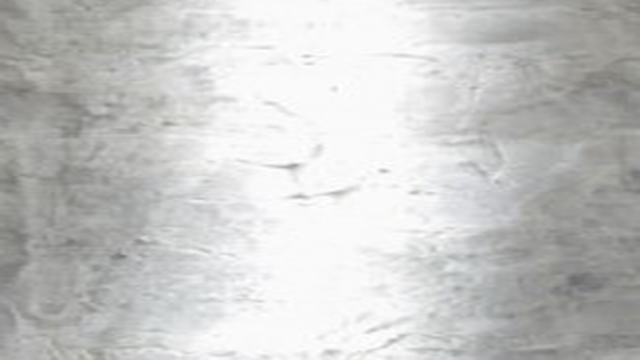

Supplement: S2 File — (ZIP) [file pone.0299471.s002.zip › 0323.png]

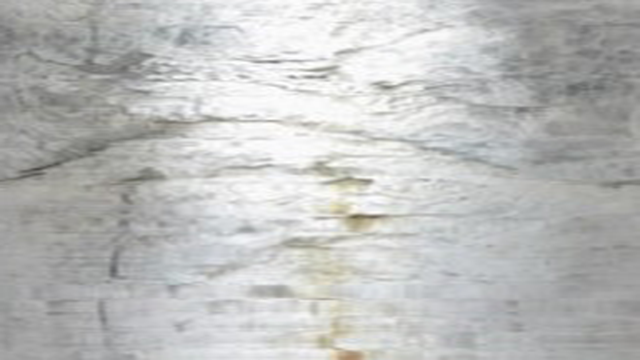

Supplement: S2 File — (ZIP) [file pone.0299471.s002.zip › 0324.png]

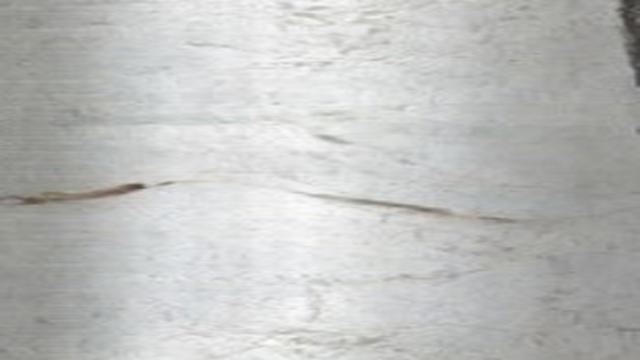

Supplement: S2 File — (ZIP) [file pone.0299471.s002.zip › 0325.jpg]

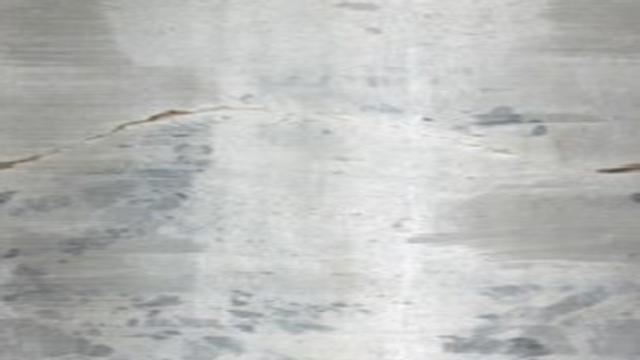

Supplement: S2 File — (ZIP) [file pone.0299471.s002.zip › 0326.jpg]

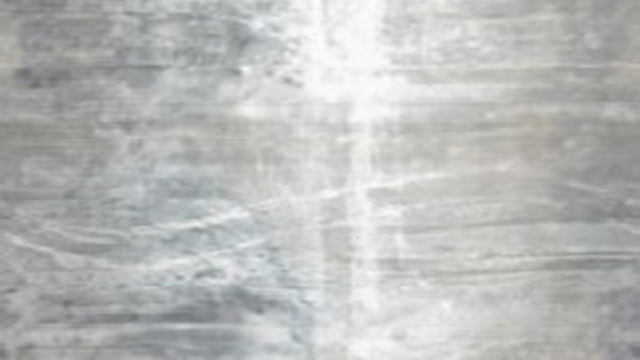

Supplement: S2 File — (ZIP) [file pone.0299471.s002.zip › 0327.png]

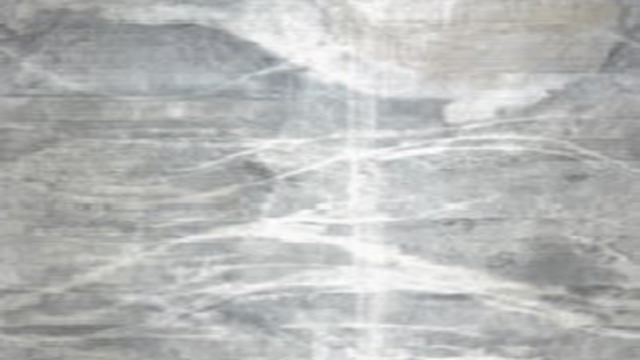

Supplement: S2 File — (ZIP) [file pone.0299471.s002.zip › 0328.png]

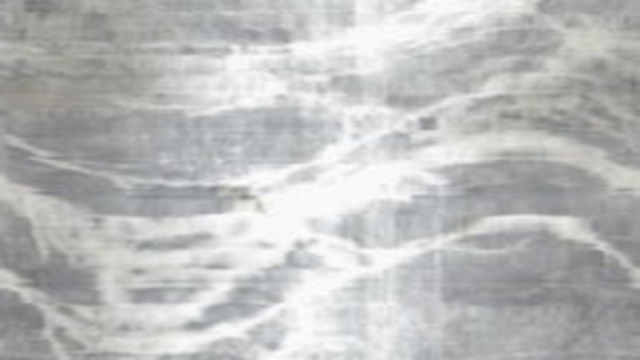

Supplement: S2 File — (ZIP) [file pone.0299471.s002.zip › 0329.png]

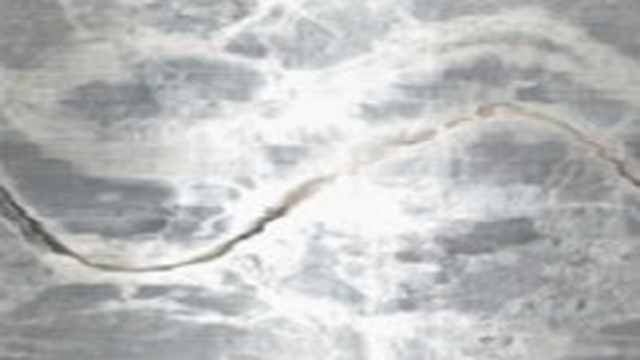

Supplement: S2 File — (ZIP) [file pone.0299471.s002.zip › 0330.png]

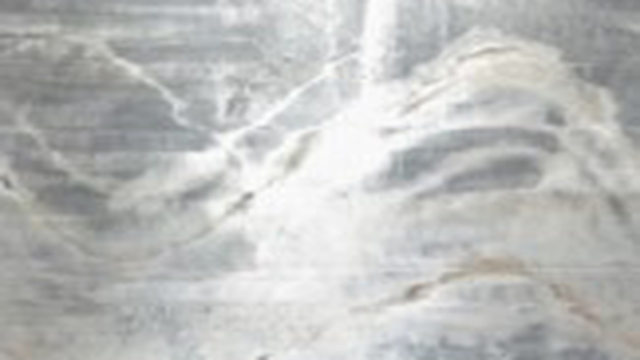

Supplement: S2 File — (ZIP) [file pone.0299471.s002.zip › 0331.png]

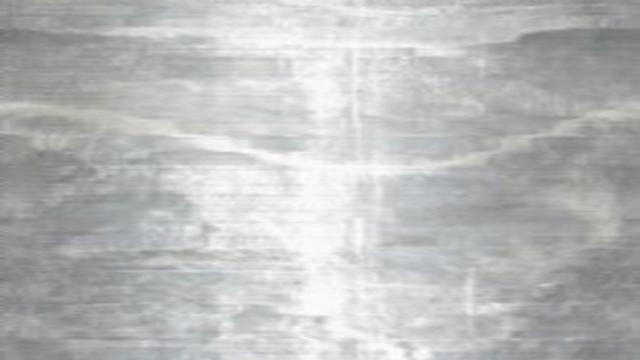

Supplement: S2 File — (ZIP) [file pone.0299471.s002.zip › 0332.jpg]

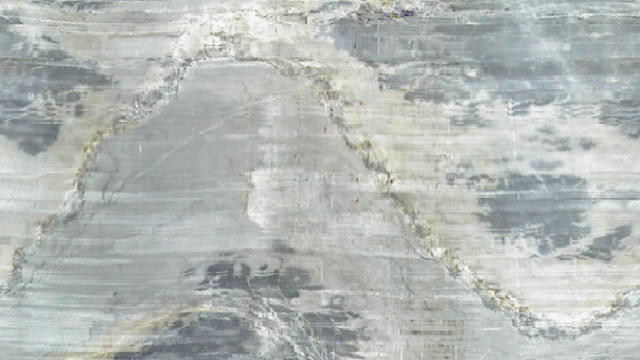

Supplement: S2 File — (ZIP) [file pone.0299471.s002.zip › 0333.png]

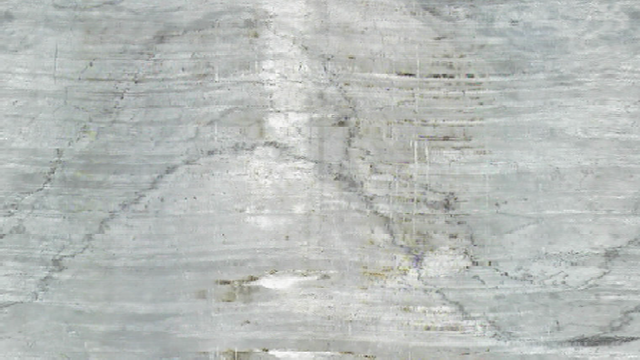

Supplement: S2 File — (ZIP) [file pone.0299471.s002.zip › 0334.png]

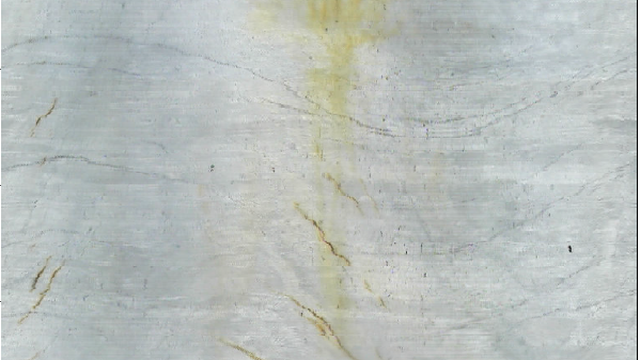

Supplement: S2 File — (ZIP) [file pone.0299471.s002.zip › 0335.png]

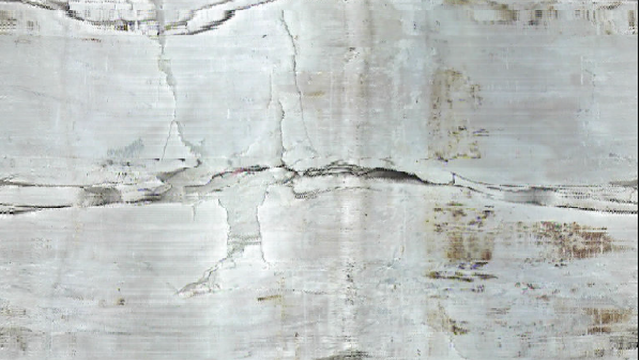

Supplement: S2 File — (ZIP) [file pone.0299471.s002.zip › 0336.png]

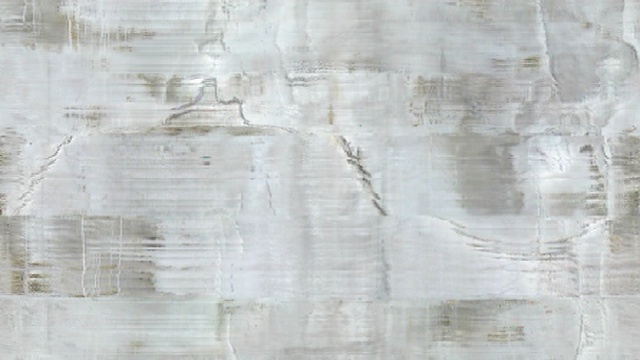

Supplement: S2 File — (ZIP) [file pone.0299471.s002.zip › 0337.png]

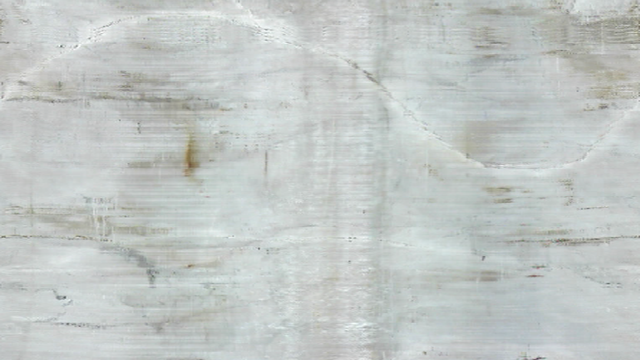

Supplement: S2 File — (ZIP) [file pone.0299471.s002.zip › 0338.png]

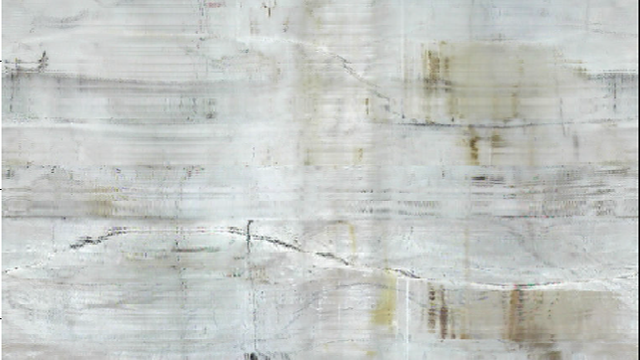

Supplement: S2 File — (ZIP) [file pone.0299471.s002.zip › 0339.png]

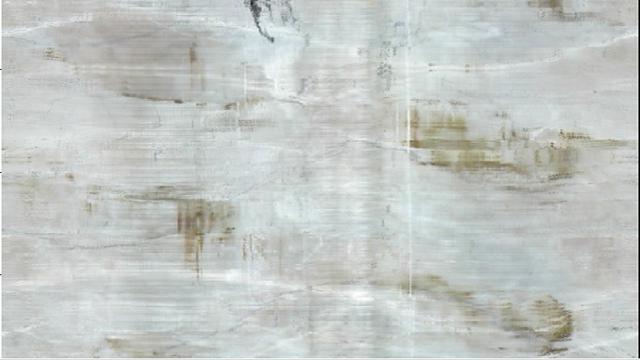

Supplement: S2 File — (ZIP) [file pone.0299471.s002.zip › 0340.png]

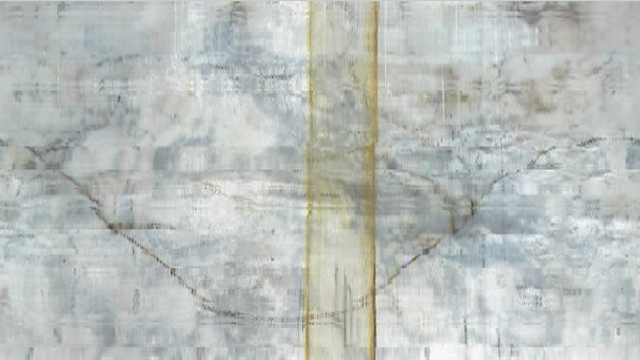

Supplement: S2 File — (ZIP) [file pone.0299471.s002.zip › 0341.png]

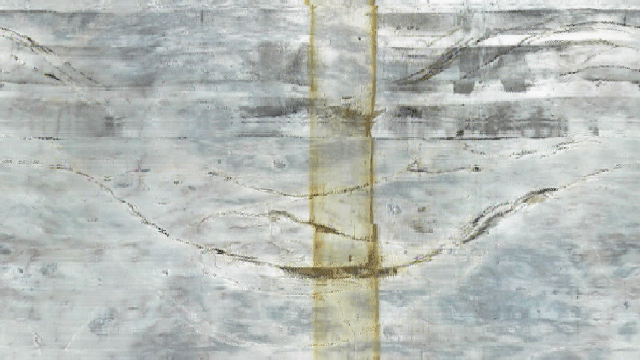

Supplement: S2 File — (ZIP) [file pone.0299471.s002.zip › 0342.png]

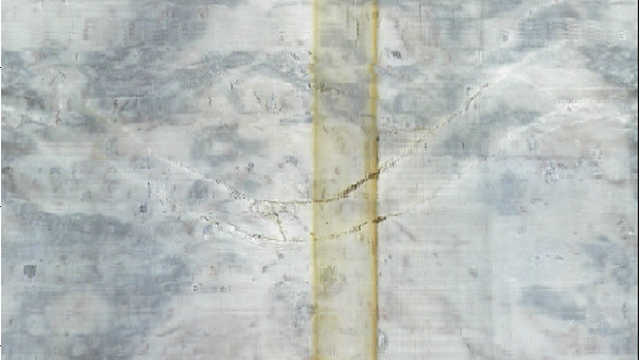

Supplement: S2 File — (ZIP) [file pone.0299471.s002.zip › 0343.png]

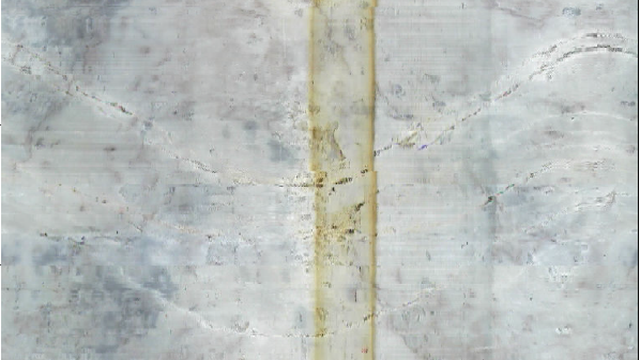

Supplement: S2 File — (ZIP) [file pone.0299471.s002.zip › 0344.png]

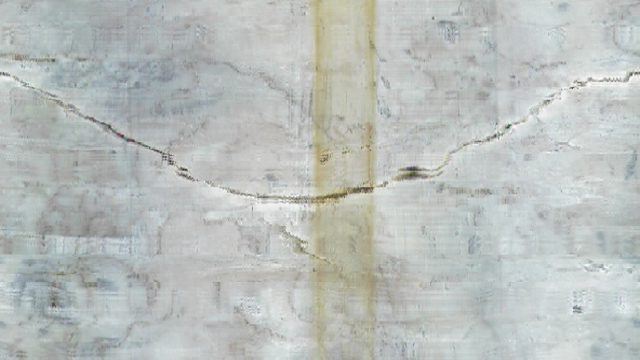

Supplement: S2 File — (ZIP) [file pone.0299471.s002.zip › 0345.png]

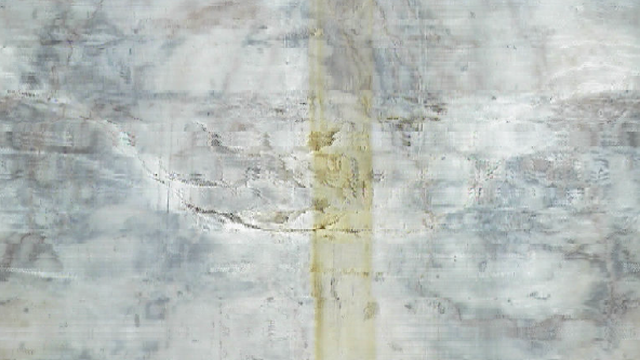

Supplement: S2 File — (ZIP) [file pone.0299471.s002.zip › 0346.png]

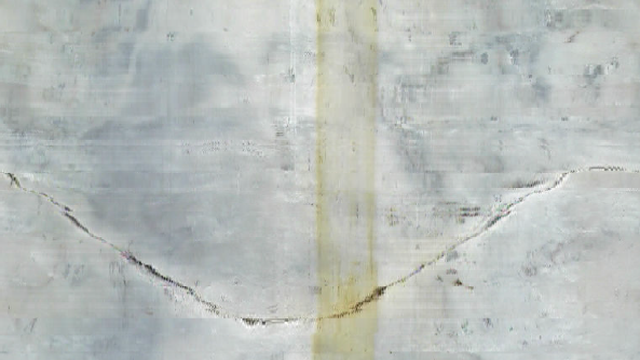

Supplement: S2 File — (ZIP) [file pone.0299471.s002.zip › 0347.png]

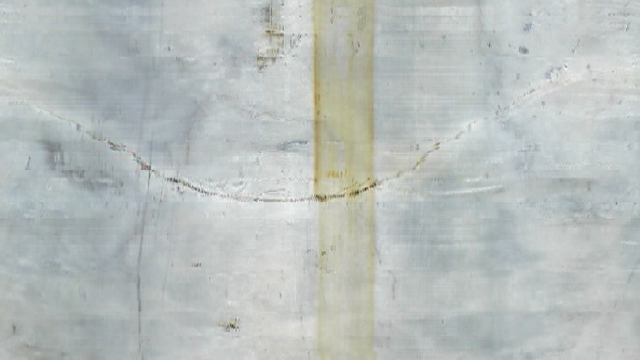

Supplement: S2 File — (ZIP) [file pone.0299471.s002.zip › 0348.png]

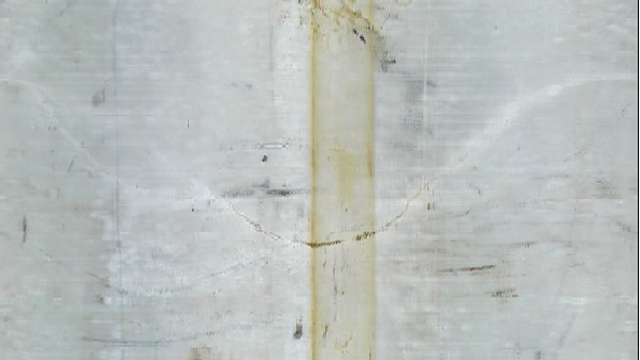

Supplement: S2 File — (ZIP) [file pone.0299471.s002.zip › 0349.png]

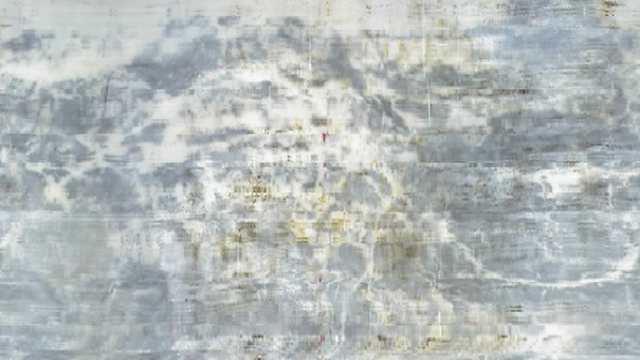

Supplement: S2 File — (ZIP) [file pone.0299471.s002.zip › 0350.png]

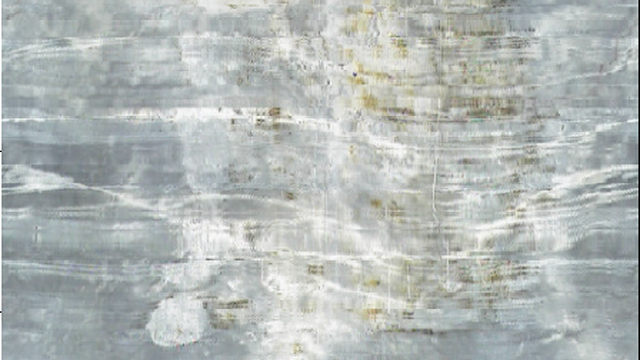

Supplement: S2 File — (ZIP) [file pone.0299471.s002.zip › 0351.png]

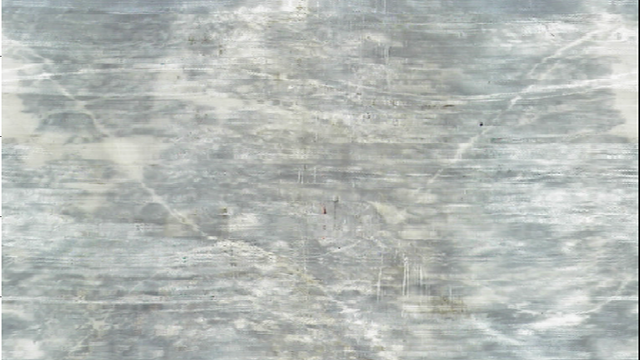

Supplement: S2 File — (ZIP) [file pone.0299471.s002.zip › 0352.png]

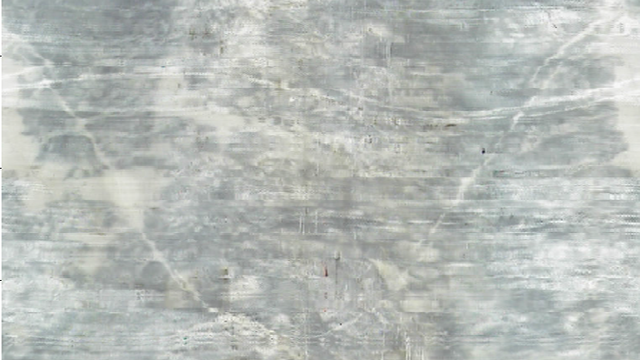

Supplement: S2 File — (ZIP) [file pone.0299471.s002.zip › 0353.png]

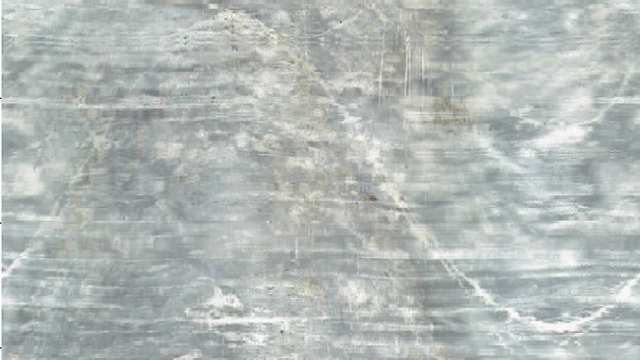

Supplement: S2 File — (ZIP) [file pone.0299471.s002.zip › 0354.png]

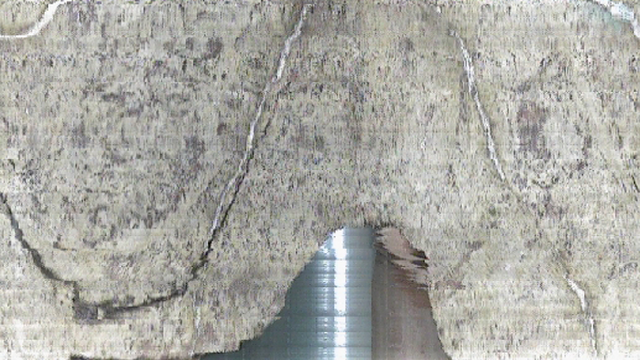

Supplement: S2 File — (ZIP) [file pone.0299471.s002.zip › 0355.png]

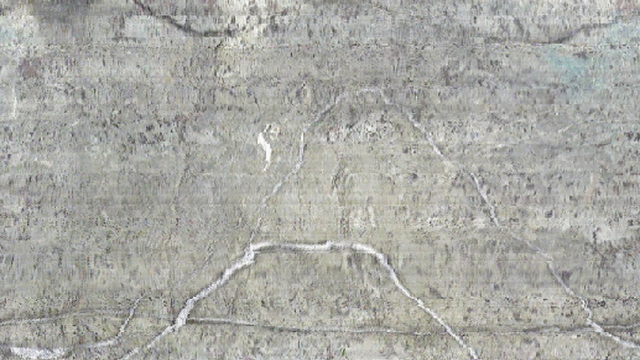

Supplement: S2 File — (ZIP) [file pone.0299471.s002.zip › 0356.png]

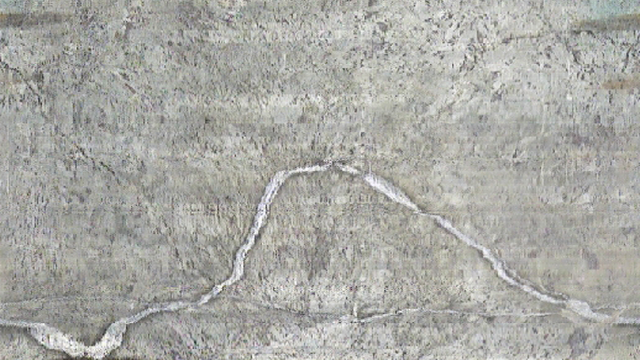

Supplement: S2 File — (ZIP) [file pone.0299471.s002.zip › 0357.png]

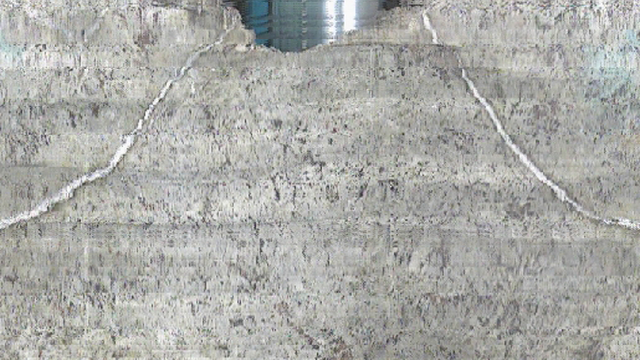

Supplement: S2 File — (ZIP) [file pone.0299471.s002.zip › 0358.png]

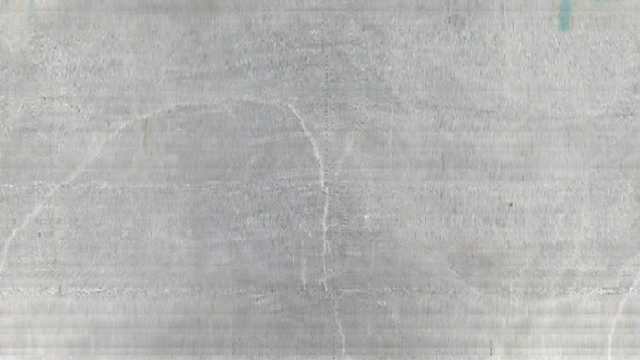

Supplement: S2 File — (ZIP) [file pone.0299471.s002.zip › 0359.png]

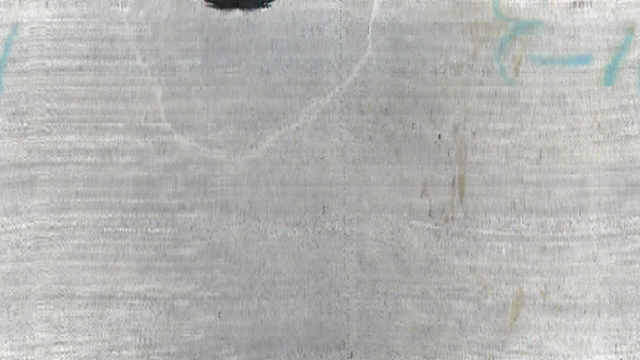

Supplement: S2 File — (ZIP) [file pone.0299471.s002.zip › 0360.png]

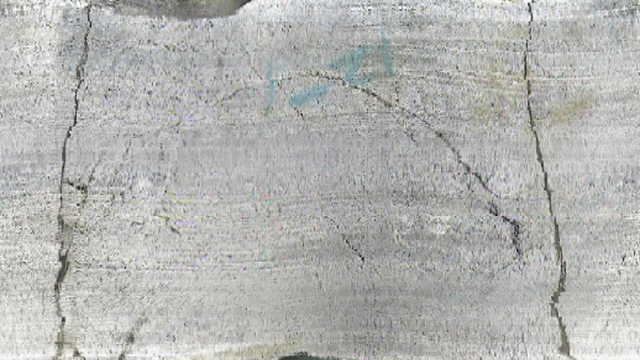

Supplement: S2 File — (ZIP) [file pone.0299471.s002.zip › 0361.png]

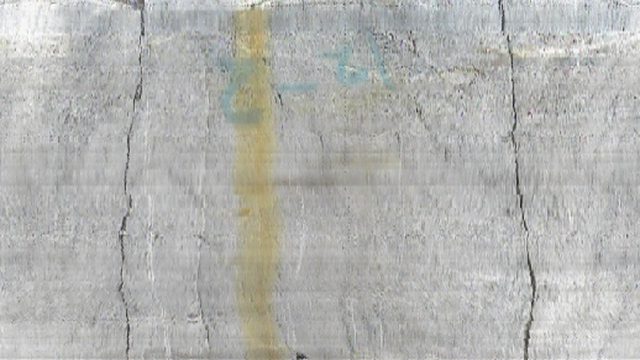

Supplement: S2 File — (ZIP) [file pone.0299471.s002.zip › 0362.png]

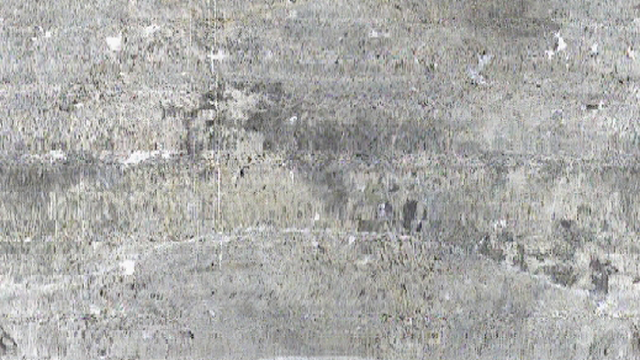

Supplement: S2 File — (ZIP) [file pone.0299471.s002.zip › 0363.png]

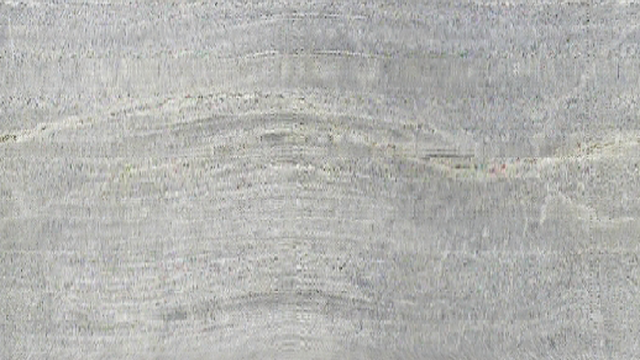

Supplement: S2 File — (ZIP) [file pone.0299471.s002.zip › 0364.png]

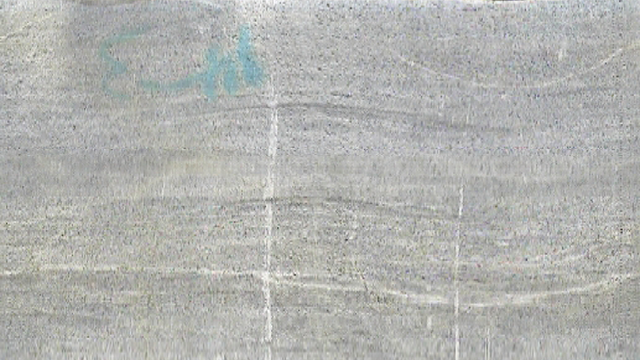

Supplement: S2 File — (ZIP) [file pone.0299471.s002.zip › 0365.png]

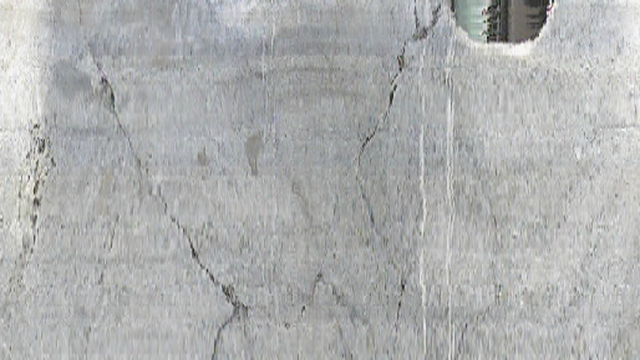

Supplement: S2 File — (ZIP) [file pone.0299471.s002.zip › 0366.png]

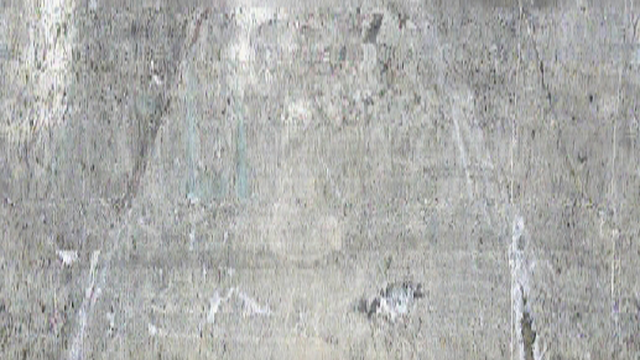

Supplement: S2 File — (ZIP) [file pone.0299471.s002.zip › 0367.png]

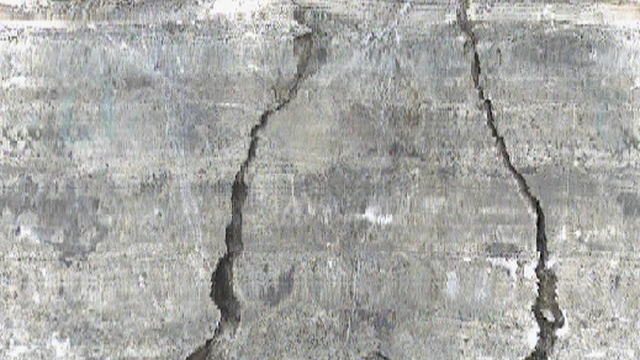

Supplement: S2 File — (ZIP) [file pone.0299471.s002.zip › 0368.png]

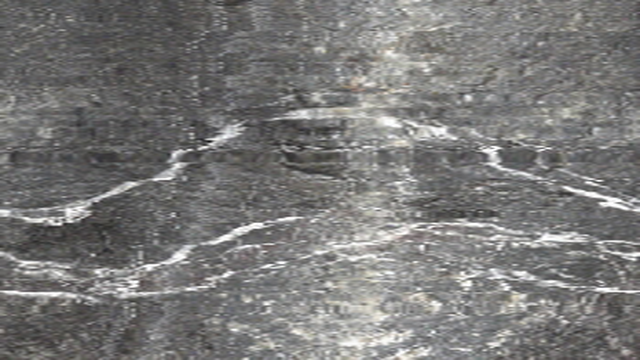

Supplement: S2 File — (ZIP) [file pone.0299471.s002.zip › 0369.png]

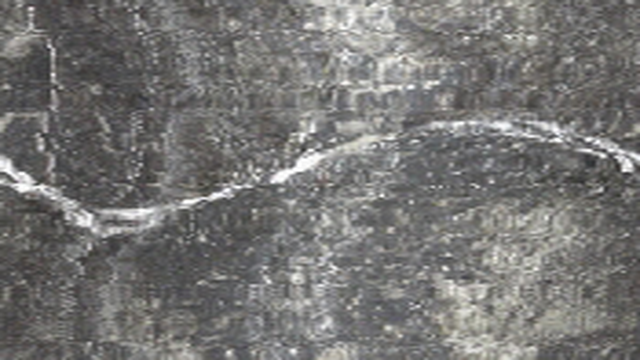

Supplement: S2 File — (ZIP) [file pone.0299471.s002.zip › 0370.png]

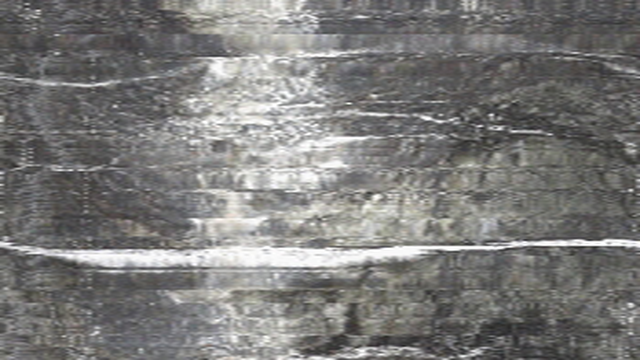

Supplement: S2 File — (ZIP) [file pone.0299471.s002.zip › 0371.png]

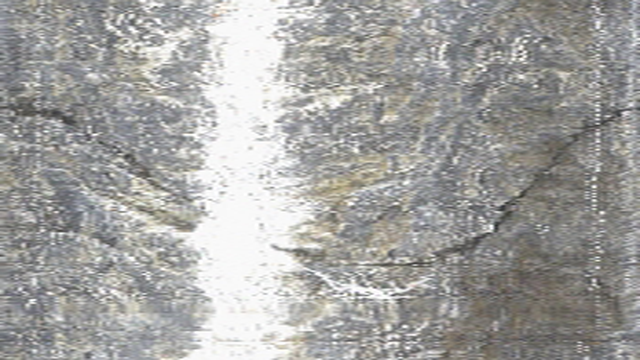

Supplement: S2 File — (ZIP) [file pone.0299471.s002.zip › 0372.png]

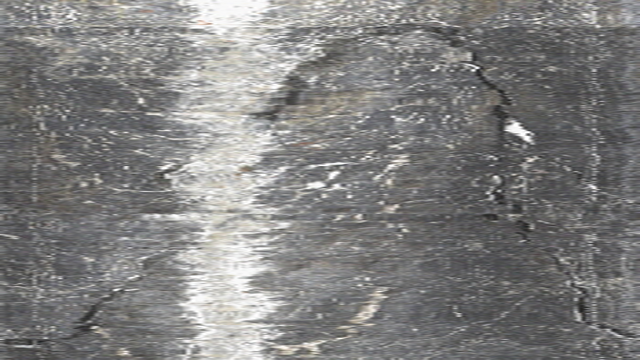

Supplement: S2 File — (ZIP) [file pone.0299471.s002.zip › 0373.png]

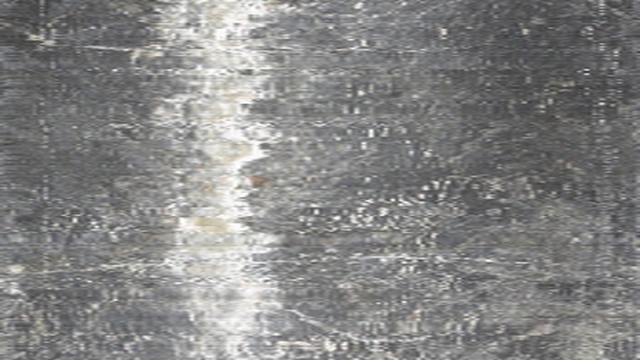

Supplement: S2 File — (ZIP) [file pone.0299471.s002.zip › 0374.png]

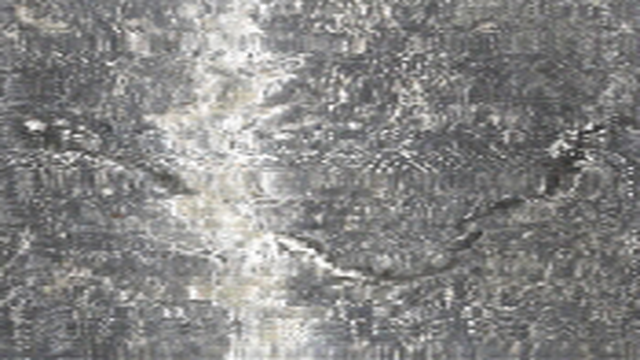

Supplement: S2 File — (ZIP) [file pone.0299471.s002.zip › 0375.png]

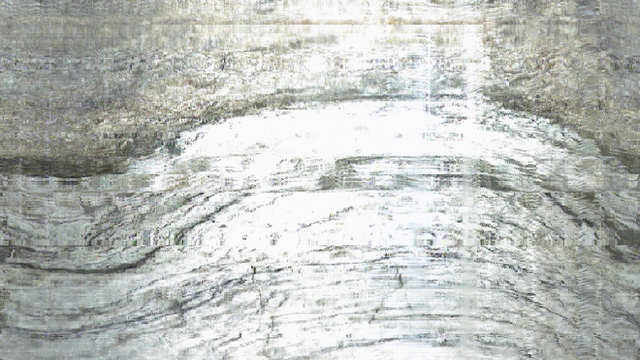

Supplement: S2 File — (ZIP) [file pone.0299471.s002.zip › 0376.png]

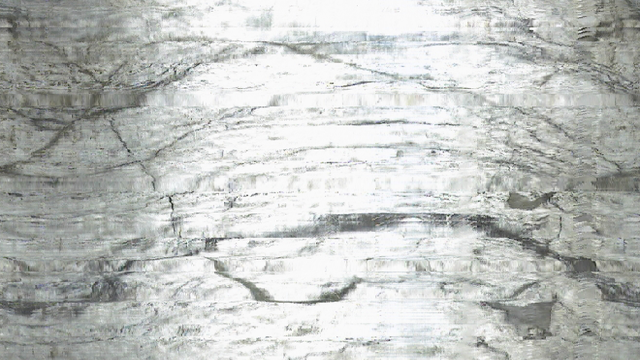

Supplement: S2 File — (ZIP) [file pone.0299471.s002.zip › 0377.png]

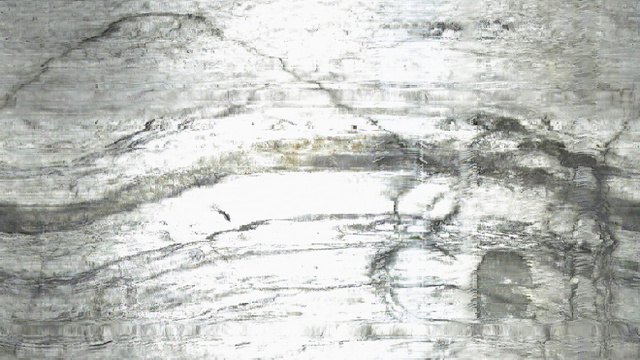

Supplement: S2 File — (ZIP) [file pone.0299471.s002.zip › 0378.png]

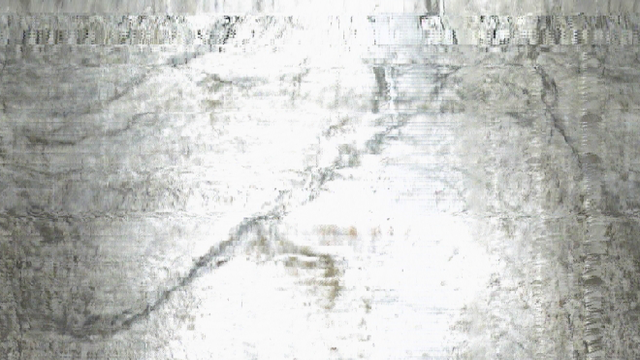

Supplement: S2 File — (ZIP) [file pone.0299471.s002.zip › 0379.png]

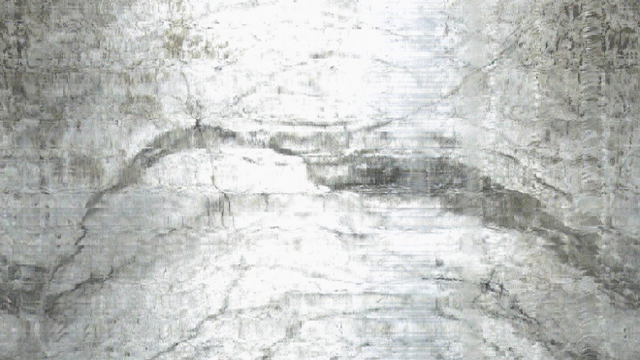

Supplement: S2 File — (ZIP) [file pone.0299471.s002.zip › 0380.png]

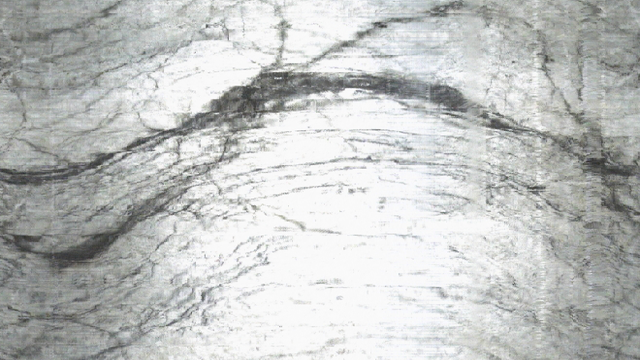

Supplement: S2 File — (ZIP) [file pone.0299471.s002.zip › 0381.png]

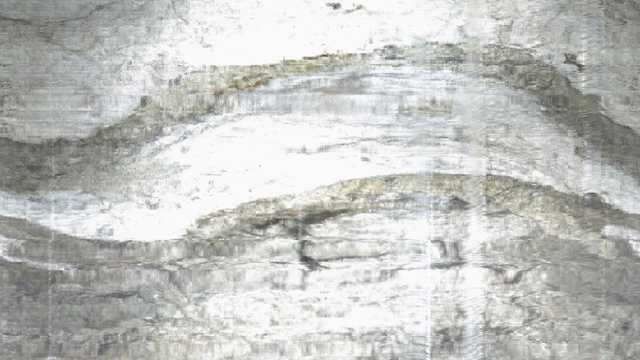

Supplement: S2 File — (ZIP) [file pone.0299471.s002.zip › 0382.png]

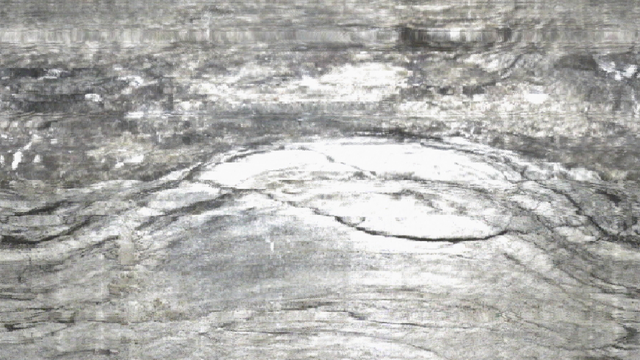

Supplement: S2 File — (ZIP) [file pone.0299471.s002.zip › 0383.png]

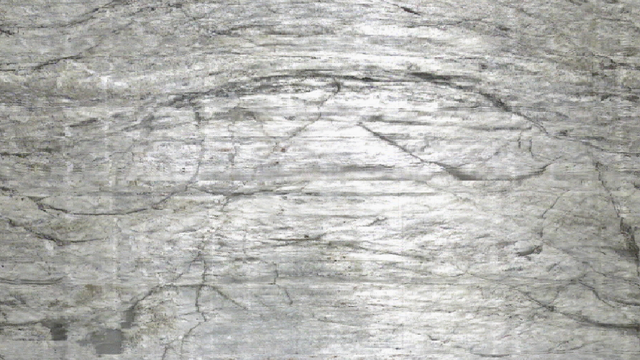

Supplement: S2 File — (ZIP) [file pone.0299471.s002.zip › 0384.png]

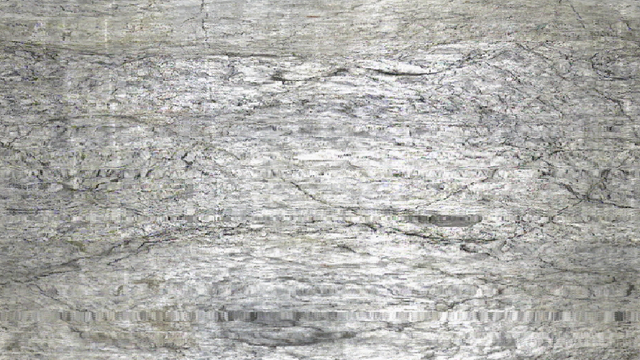

Supplement: S2 File — (ZIP) [file pone.0299471.s002.zip › 0385.png]

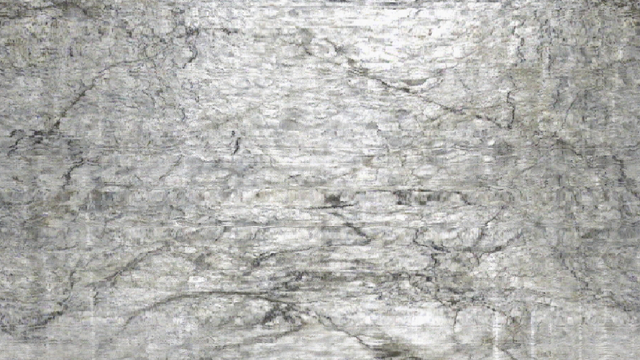

Supplement: S2 File — (ZIP) [file pone.0299471.s002.zip › 0386.png]

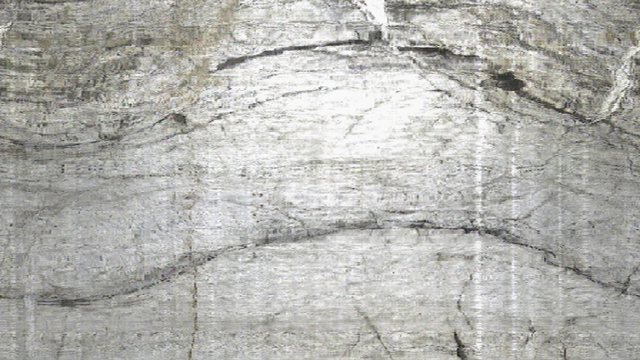

Supplement: S2 File — (ZIP) [file pone.0299471.s002.zip › 0387.png]

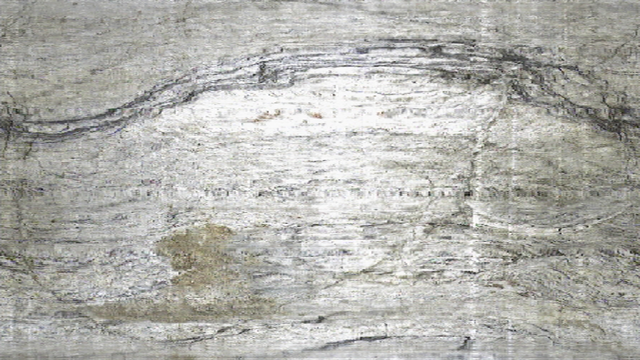

Supplement: S2 File — (ZIP) [file pone.0299471.s002.zip › 0388.png]

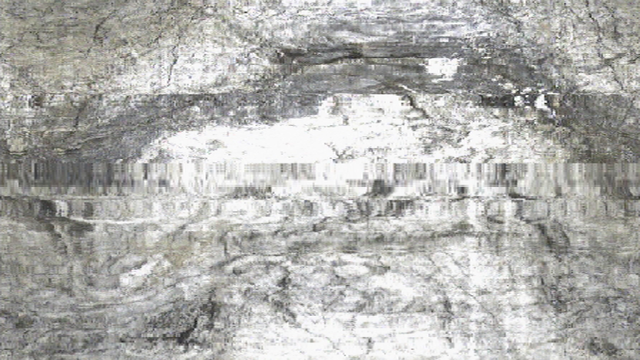

Supplement: S2 File — (ZIP) [file pone.0299471.s002.zip › 0389.png]

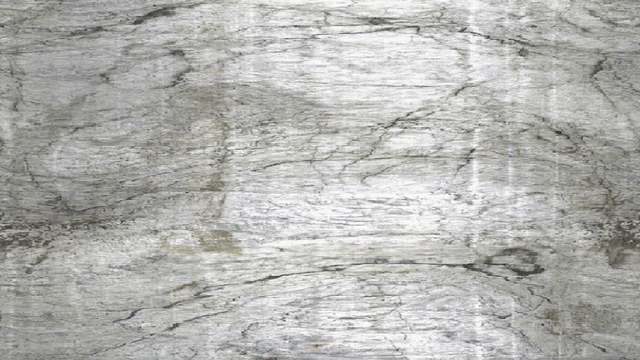

Supplement: S2 File — (ZIP) [file pone.0299471.s002.zip › 0390.png]

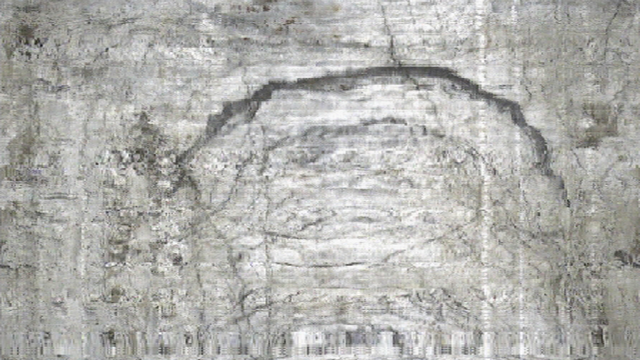

Supplement: S2 File — (ZIP) [file pone.0299471.s002.zip › 0391.png]

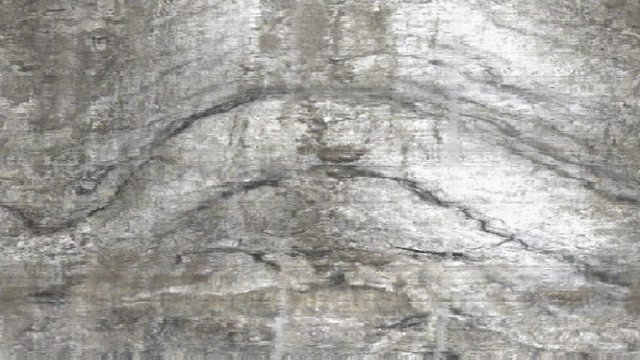

Supplement: S2 File — (ZIP) [file pone.0299471.s002.zip › 0392.png]

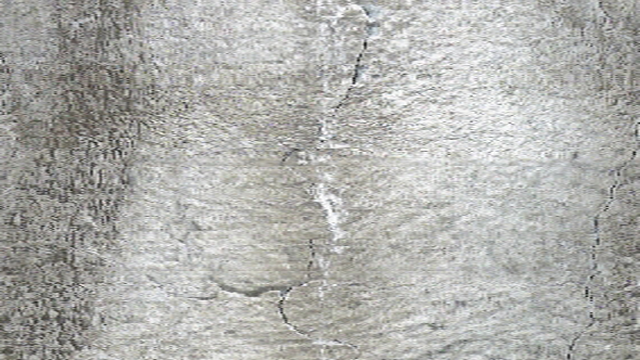

Supplement: S2 File — (ZIP) [file pone.0299471.s002.zip › 0393.png]

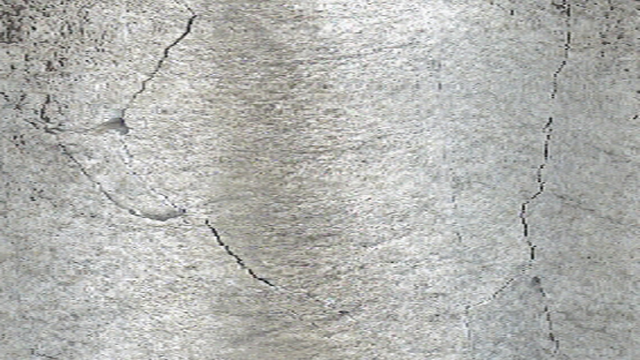

Supplement: S2 File — (ZIP) [file pone.0299471.s002.zip › 0394.png]

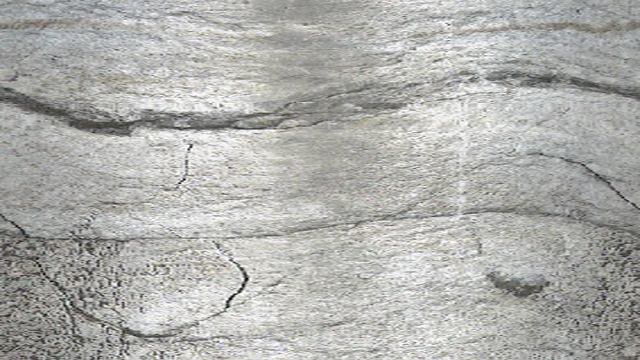

Supplement: S2 File — (ZIP) [file pone.0299471.s002.zip › 0395.png]

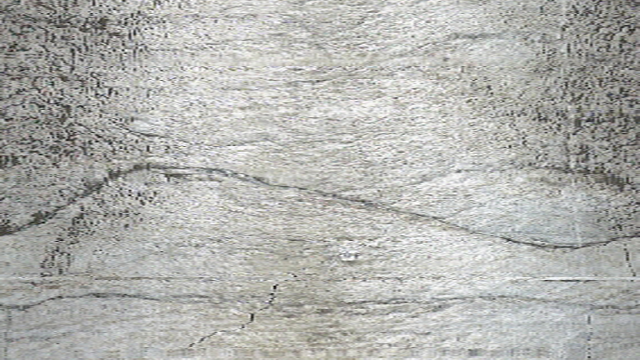

Supplement: S2 File — (ZIP) [file pone.0299471.s002.zip › 0396.png]

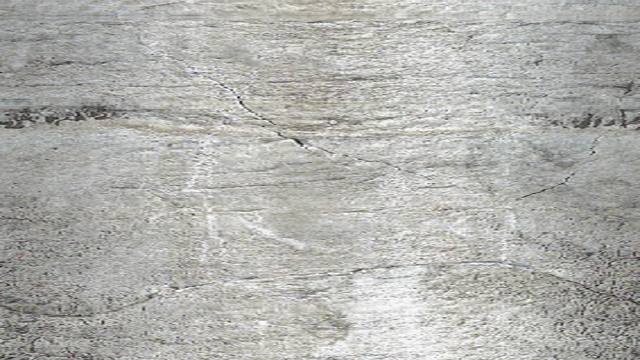

Supplement: S2 File — (ZIP) [file pone.0299471.s002.zip › 0397.png]
