# Supplementary figures and images for: The segmentation and intelligent recognition of structural surfaces in borehole images based on the U2-Net network (part 3 of 4)
Source: PLoS One. 2024 Mar 7;19(3):e0299471. doi: 10.1371/journal.pone.0299471 (PMC10919631; doi:10.1371/journal.pone.0299471)

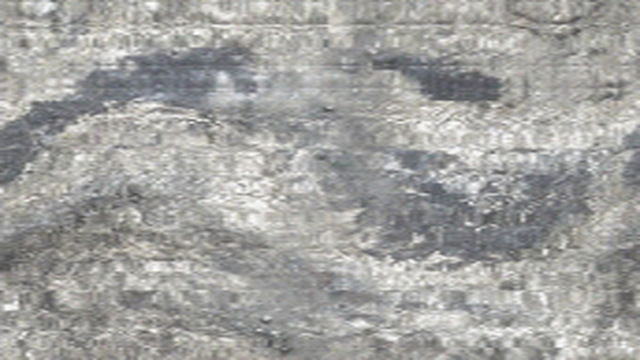

Supplement: S2 File — (ZIP) [file pone.0299471.s002.zip › 0200.png]

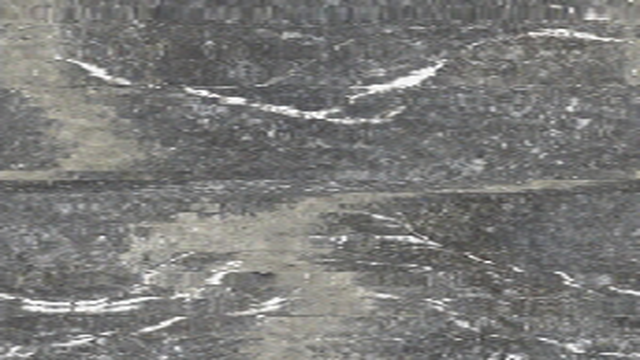

Supplement: S2 File — (ZIP) [file pone.0299471.s002.zip › 0201.png]

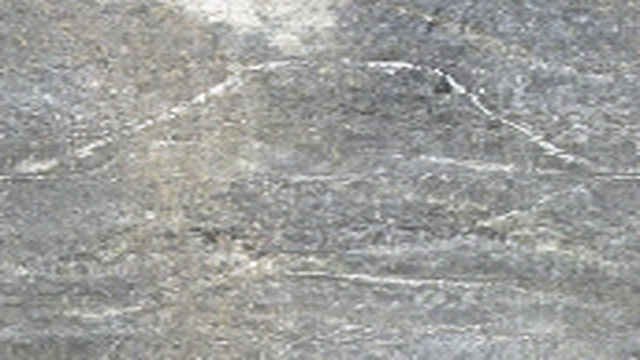

Supplement: S2 File — (ZIP) [file pone.0299471.s002.zip › 0202.png]

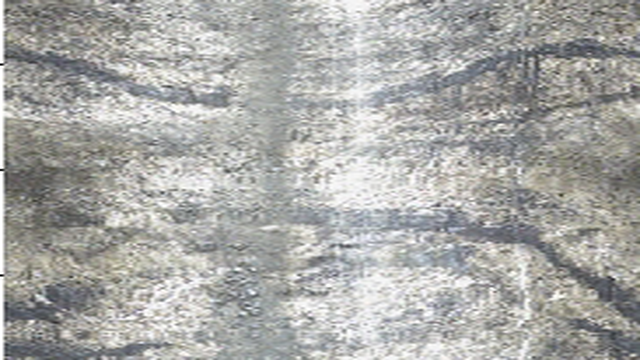

Supplement: S2 File — (ZIP) [file pone.0299471.s002.zip › 0203.png]

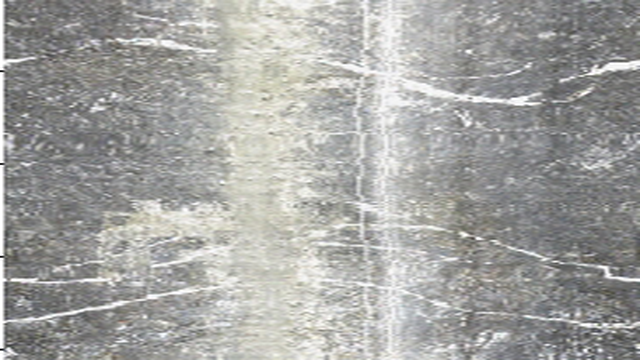

Supplement: S2 File — (ZIP) [file pone.0299471.s002.zip › 0204.png]

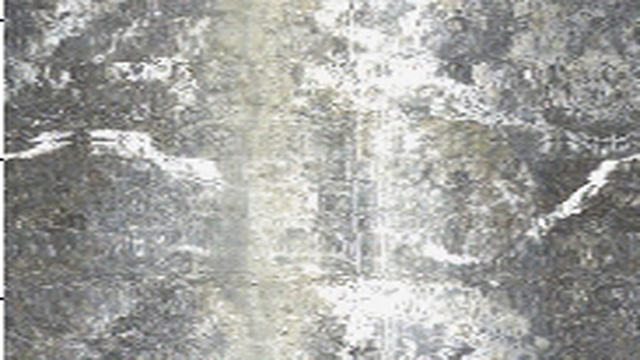

Supplement: S2 File — (ZIP) [file pone.0299471.s002.zip › 0205.png]

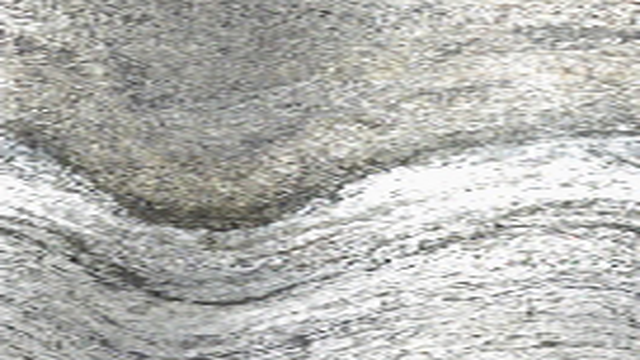

Supplement: S2 File — (ZIP) [file pone.0299471.s002.zip › 0206.png]

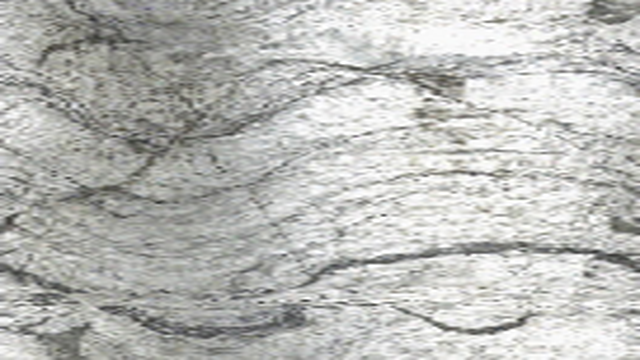

Supplement: S2 File — (ZIP) [file pone.0299471.s002.zip › 0207.png]

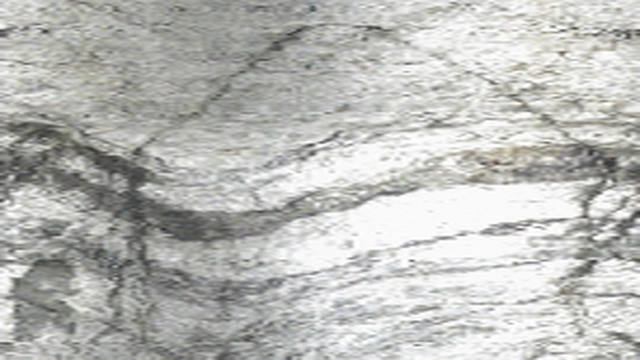

Supplement: S2 File — (ZIP) [file pone.0299471.s002.zip › 0208.png]

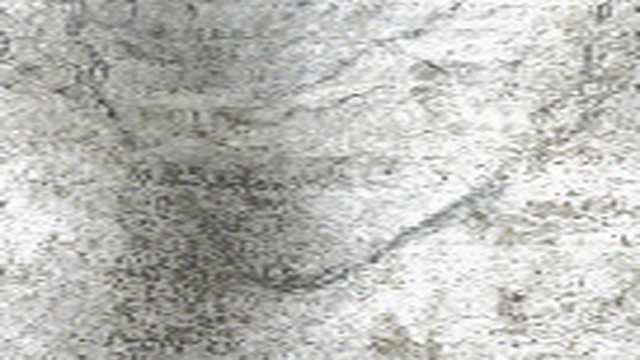

Supplement: S2 File — (ZIP) [file pone.0299471.s002.zip › 0209.png]

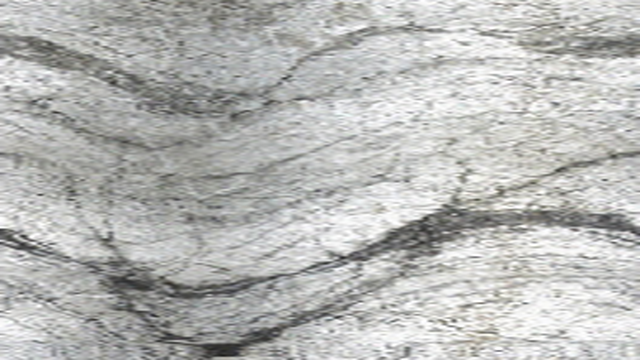

Supplement: S2 File — (ZIP) [file pone.0299471.s002.zip › 0210.png]

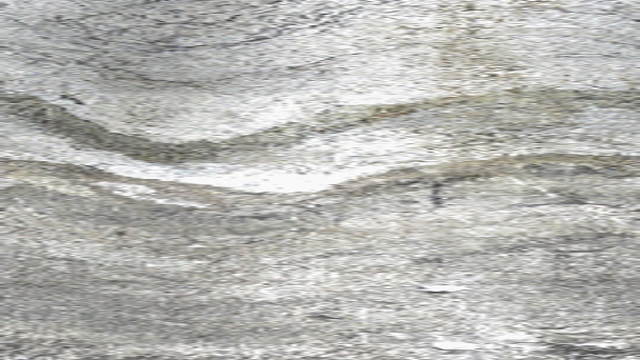

Supplement: S2 File — (ZIP) [file pone.0299471.s002.zip › 0211.png]

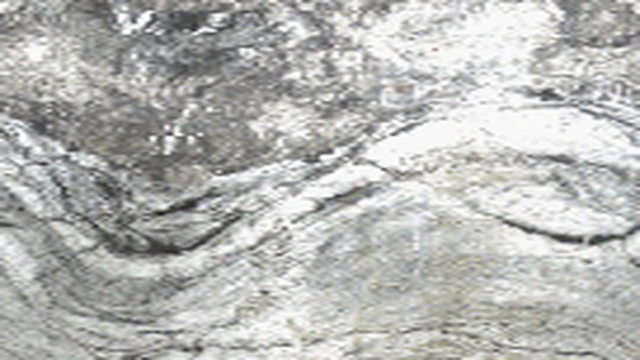

Supplement: S2 File — (ZIP) [file pone.0299471.s002.zip › 0212.png]

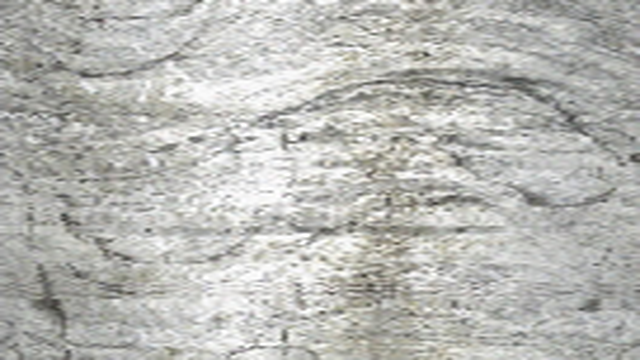

Supplement: S2 File — (ZIP) [file pone.0299471.s002.zip › 0213.png]

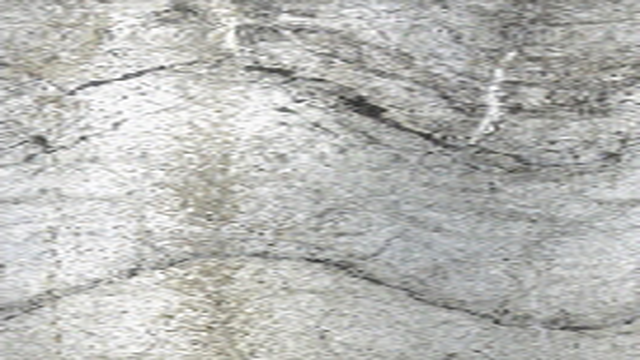

Supplement: S2 File — (ZIP) [file pone.0299471.s002.zip › 0214.png]

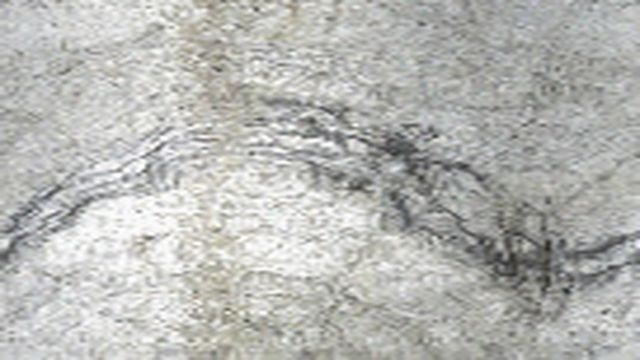

Supplement: S2 File — (ZIP) [file pone.0299471.s002.zip › 0215.png]

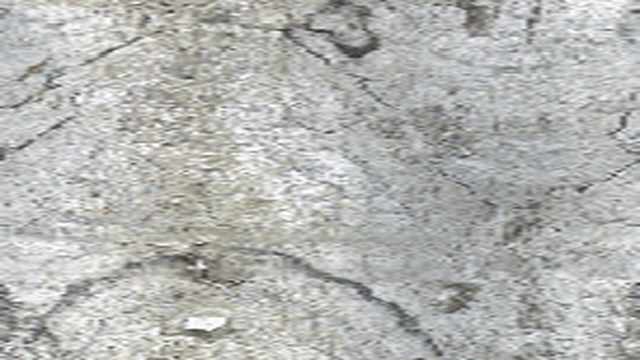

Supplement: S2 File — (ZIP) [file pone.0299471.s002.zip › 0216.png]

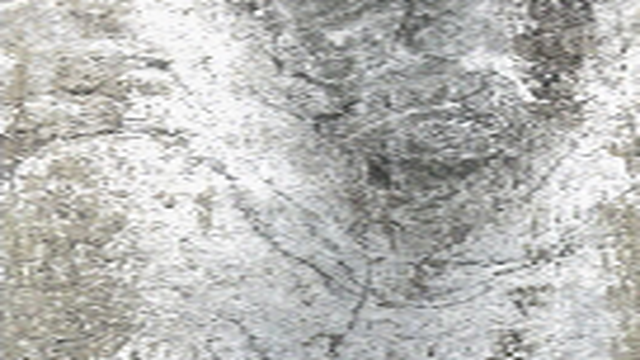

Supplement: S2 File — (ZIP) [file pone.0299471.s002.zip › 0217.png]

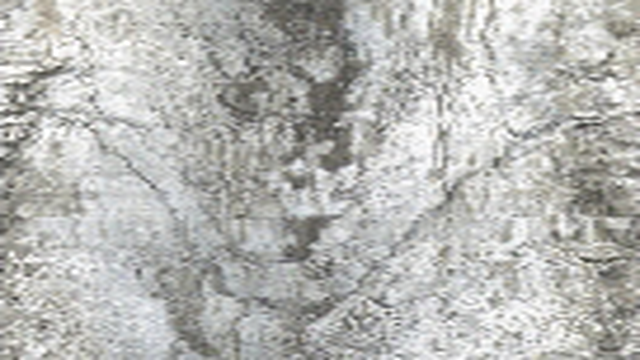

Supplement: S2 File — (ZIP) [file pone.0299471.s002.zip › 0218.png]

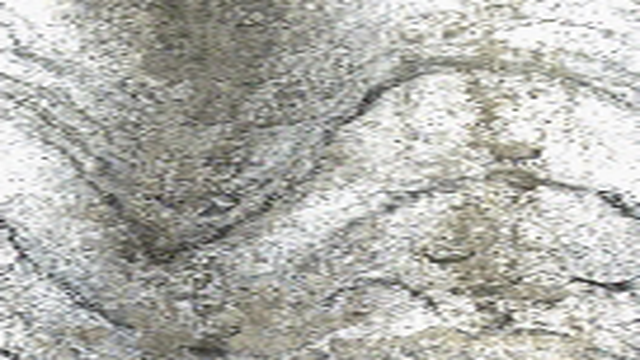

Supplement: S2 File — (ZIP) [file pone.0299471.s002.zip › 0219.png]

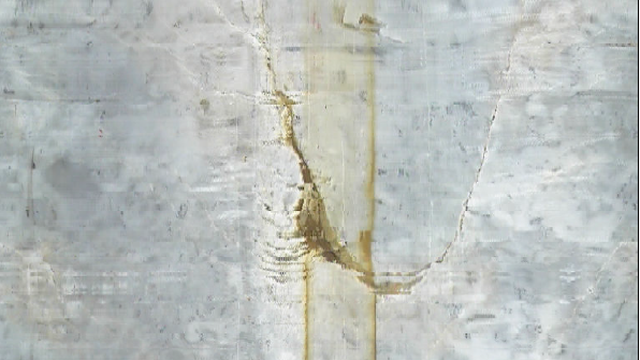

Supplement: S2 File — (ZIP) [file pone.0299471.s002.zip › 0220.png]

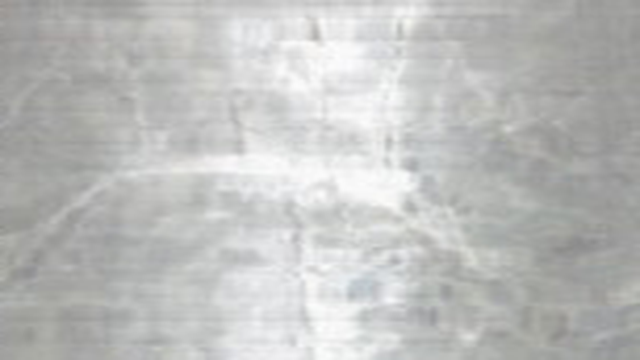

Supplement: S2 File — (ZIP) [file pone.0299471.s002.zip › 0221.png]

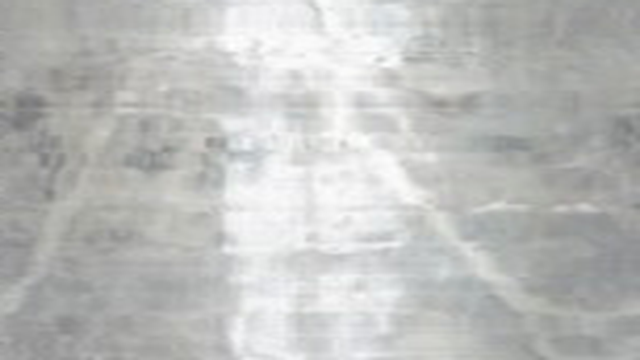

Supplement: S2 File — (ZIP) [file pone.0299471.s002.zip › 0222.png]

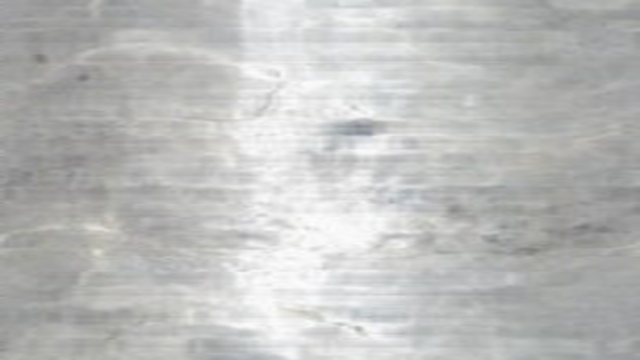

Supplement: S2 File — (ZIP) [file pone.0299471.s002.zip › 0223.png]

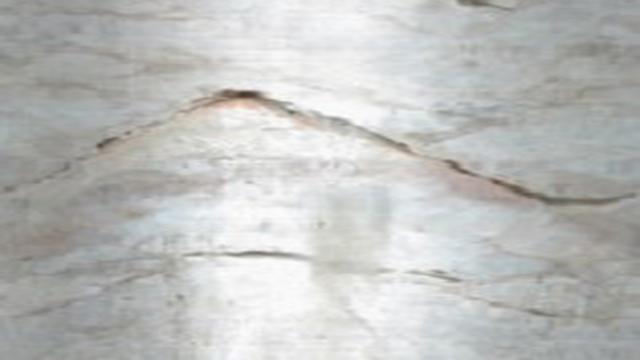

Supplement: S2 File — (ZIP) [file pone.0299471.s002.zip › 0224.jpg]

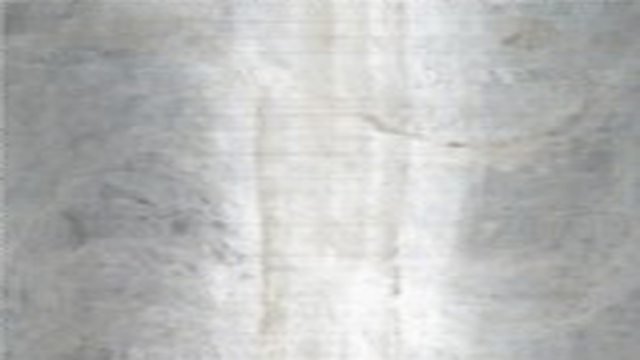

Supplement: S2 File — (ZIP) [file pone.0299471.s002.zip › 0225.png]

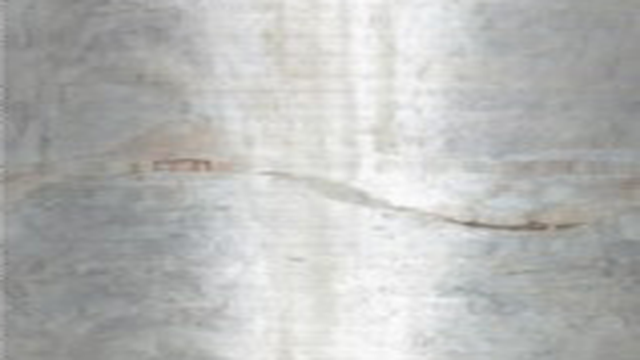

Supplement: S2 File — (ZIP) [file pone.0299471.s002.zip › 0226.png]

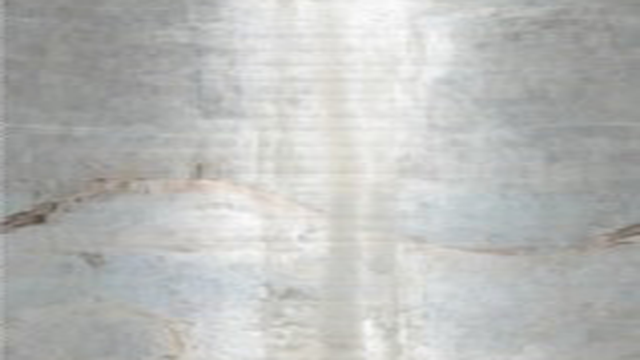

Supplement: S2 File — (ZIP) [file pone.0299471.s002.zip › 0227.png]

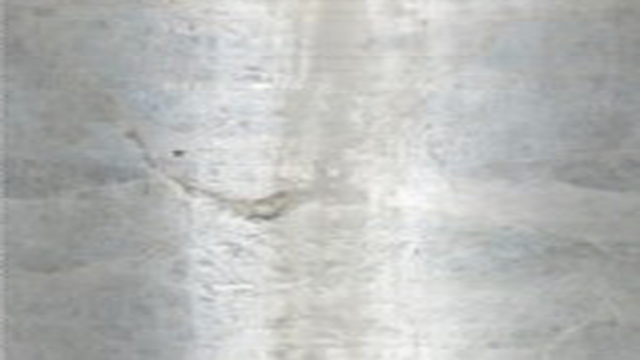

Supplement: S2 File — (ZIP) [file pone.0299471.s002.zip › 0228.png]

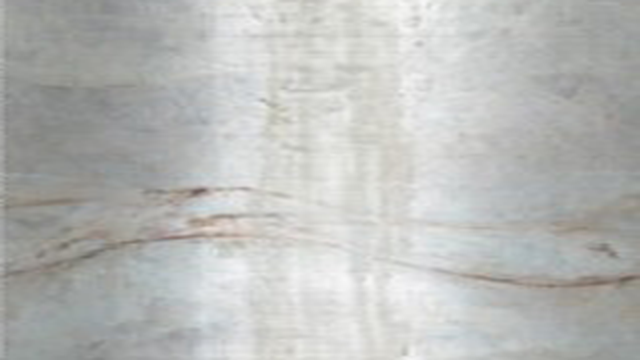

Supplement: S2 File — (ZIP) [file pone.0299471.s002.zip › 0229.png]

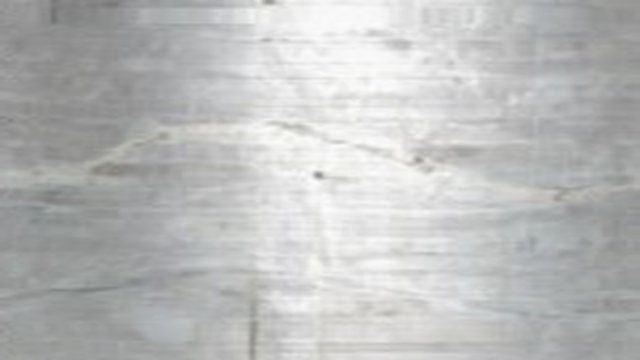

Supplement: S2 File — (ZIP) [file pone.0299471.s002.zip › 0230.png]

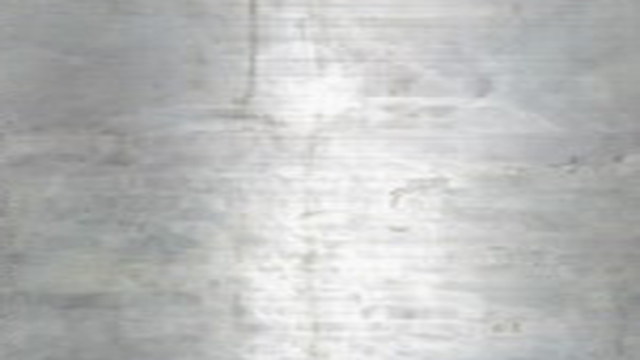

Supplement: S2 File — (ZIP) [file pone.0299471.s002.zip › 0231.png]

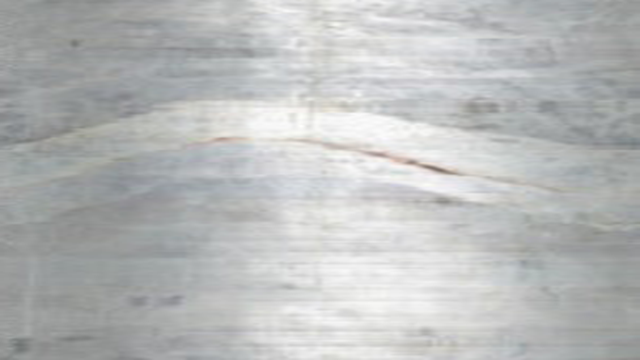

Supplement: S2 File — (ZIP) [file pone.0299471.s002.zip › 0232.png]

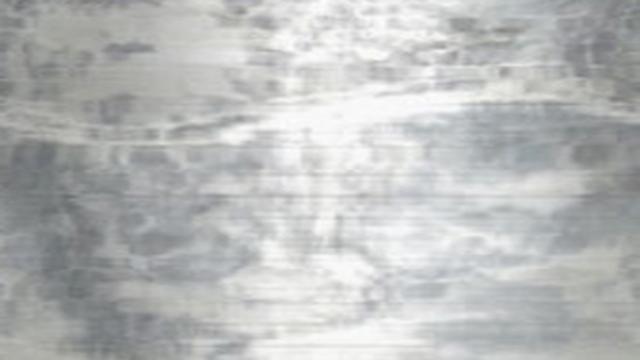

Supplement: S2 File — (ZIP) [file pone.0299471.s002.zip › 0233.jpg]

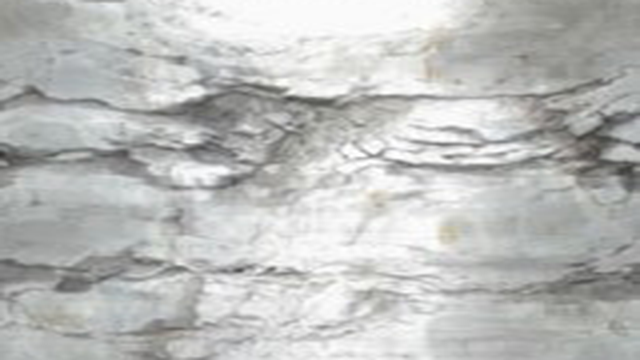

Supplement: S2 File — (ZIP) [file pone.0299471.s002.zip › 0234.png]

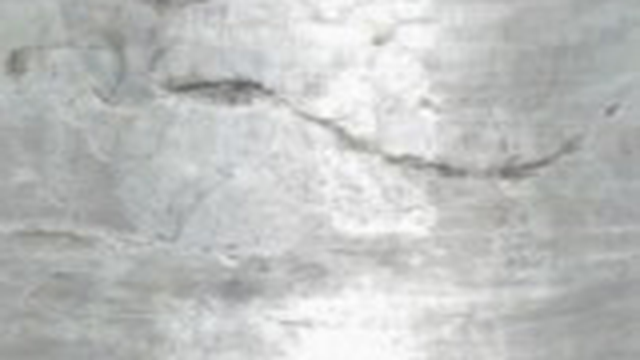

Supplement: S2 File — (ZIP) [file pone.0299471.s002.zip › 0235.png]

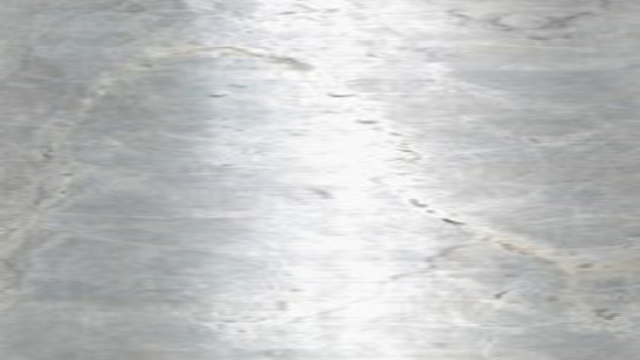

Supplement: S2 File — (ZIP) [file pone.0299471.s002.zip › 0236.png]

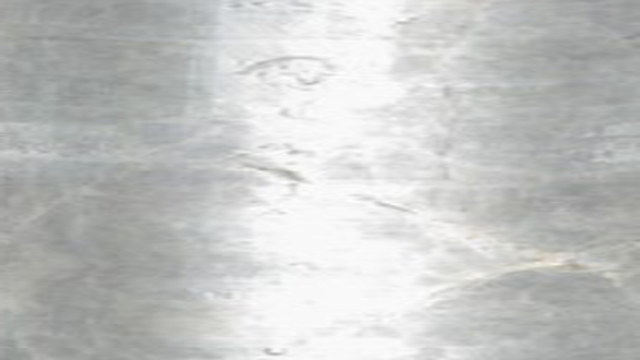

Supplement: S2 File — (ZIP) [file pone.0299471.s002.zip › 0237.png]

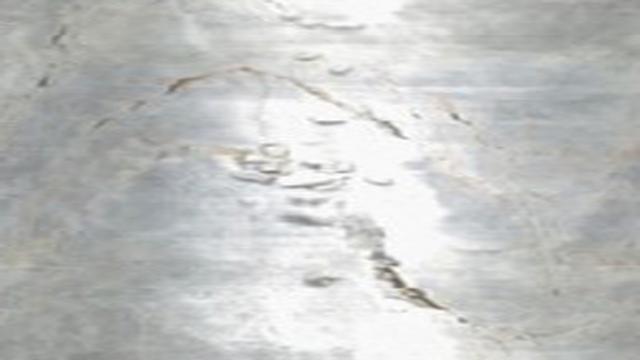

Supplement: S2 File — (ZIP) [file pone.0299471.s002.zip › 0238.jpg]

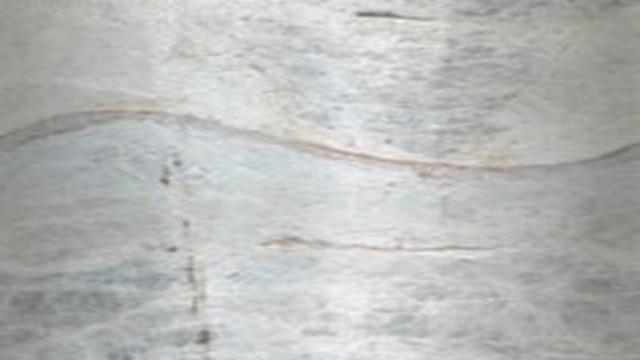

Supplement: S2 File — (ZIP) [file pone.0299471.s002.zip › 0239.jpg]

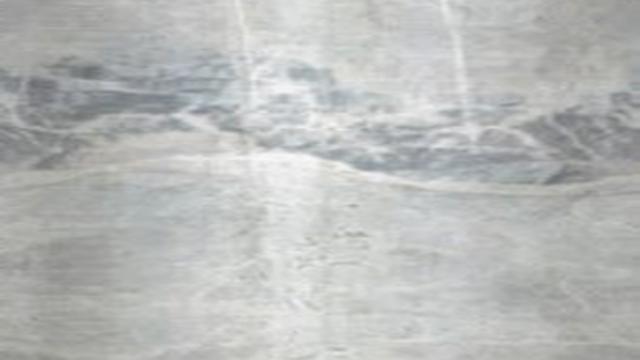

Supplement: S2 File — (ZIP) [file pone.0299471.s002.zip › 0240.jpg]

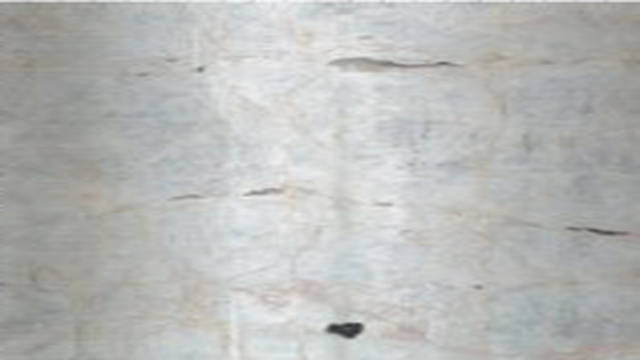

Supplement: S2 File — (ZIP) [file pone.0299471.s002.zip › 0241.png]

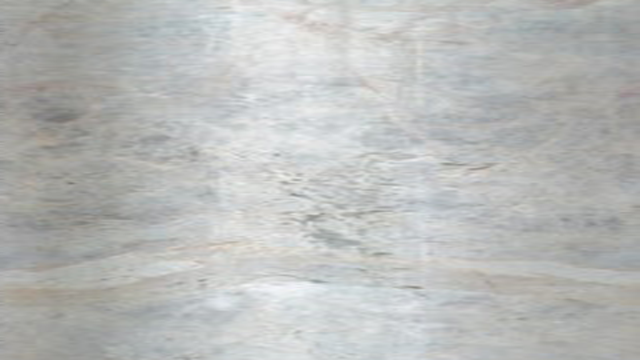

Supplement: S2 File — (ZIP) [file pone.0299471.s002.zip › 0242.png]

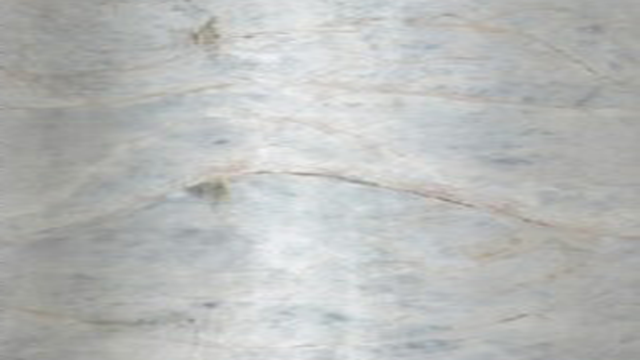

Supplement: S2 File — (ZIP) [file pone.0299471.s002.zip › 0243.png]

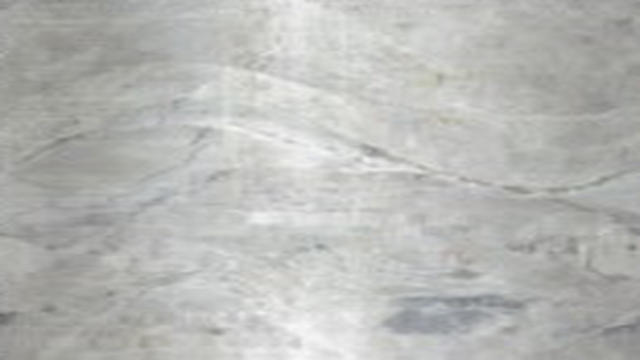

Supplement: S2 File — (ZIP) [file pone.0299471.s002.zip › 0244.png]

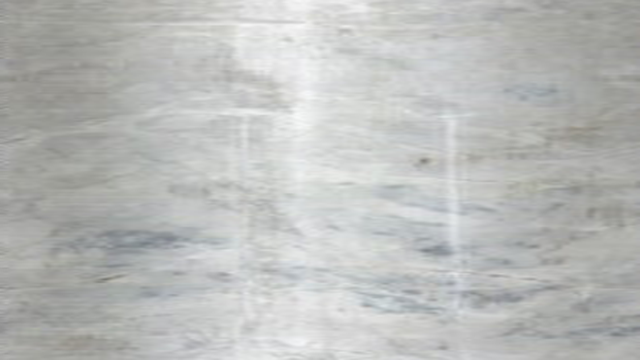

Supplement: S2 File — (ZIP) [file pone.0299471.s002.zip › 0245.png]

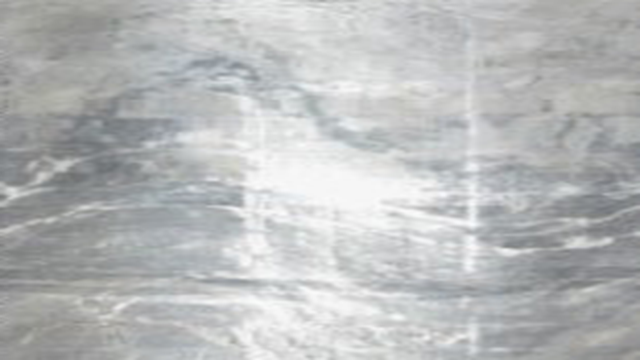

Supplement: S2 File — (ZIP) [file pone.0299471.s002.zip › 0246.png]

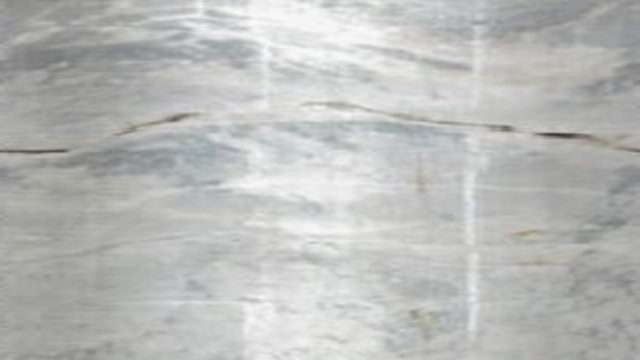

Supplement: S2 File — (ZIP) [file pone.0299471.s002.zip › 0247.png]

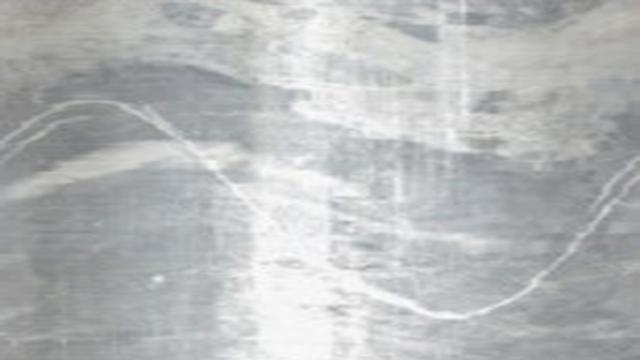

Supplement: S2 File — (ZIP) [file pone.0299471.s002.zip › 0248.jpg]

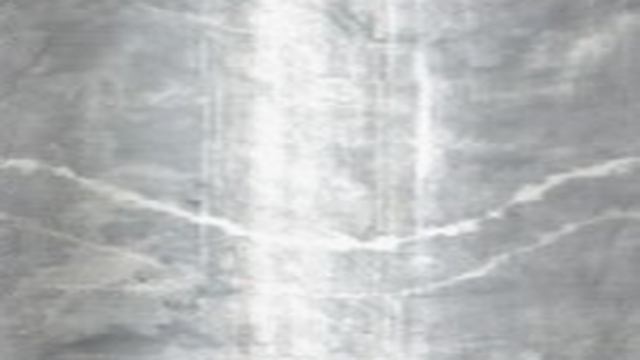

Supplement: S2 File — (ZIP) [file pone.0299471.s002.zip › 0249.png]

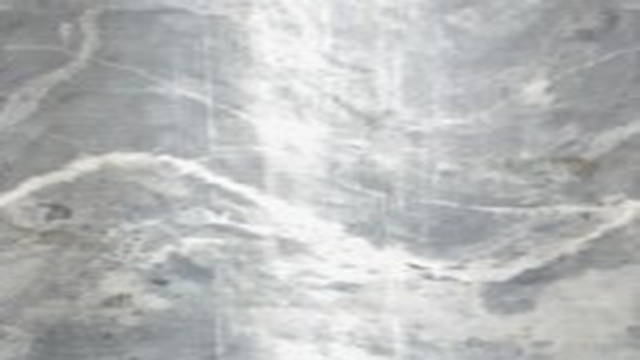

Supplement: S2 File — (ZIP) [file pone.0299471.s002.zip › 0250.png]

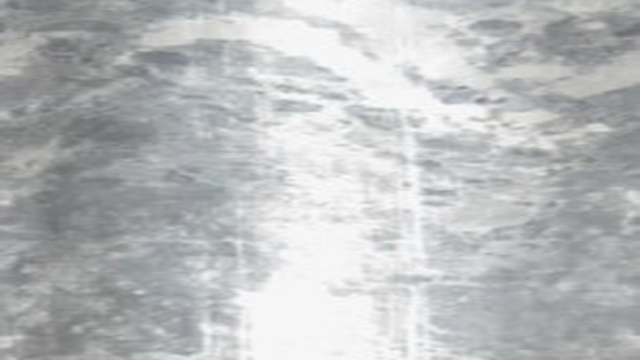

Supplement: S2 File — (ZIP) [file pone.0299471.s002.zip › 0251.png]

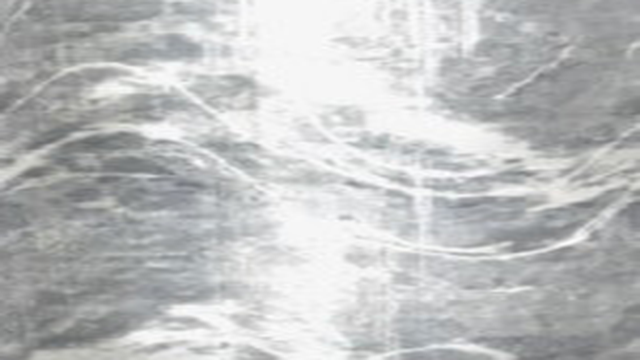

Supplement: S2 File — (ZIP) [file pone.0299471.s002.zip › 0252.png]

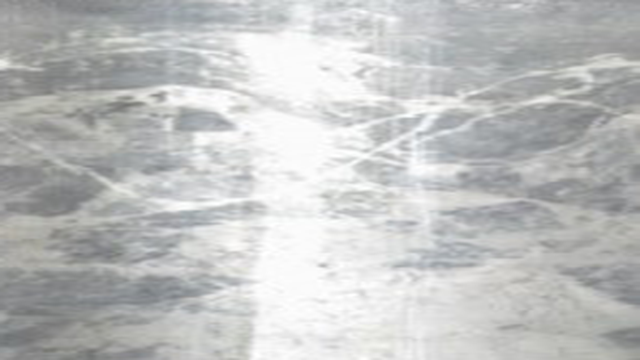

Supplement: S2 File — (ZIP) [file pone.0299471.s002.zip › 0253.png]

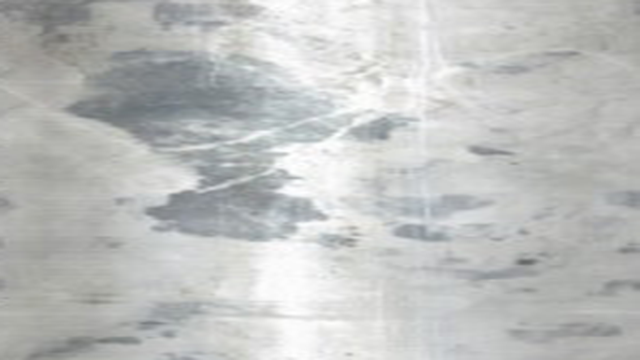

Supplement: S2 File — (ZIP) [file pone.0299471.s002.zip › 0254.png]

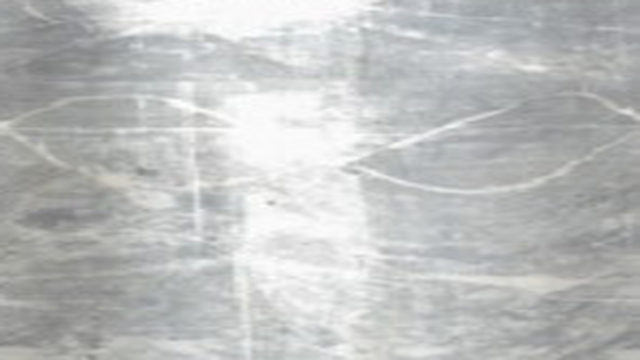

Supplement: S2 File — (ZIP) [file pone.0299471.s002.zip › 0255.png]

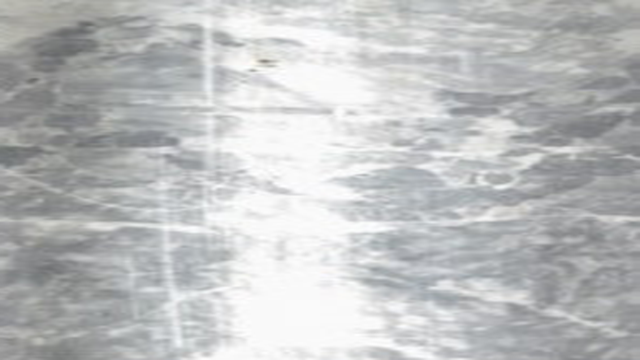

Supplement: S2 File — (ZIP) [file pone.0299471.s002.zip › 0256.png]

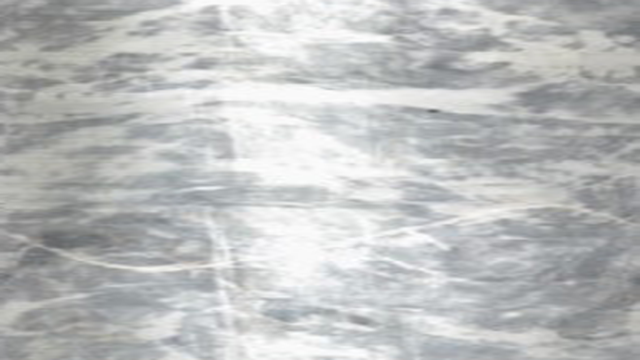

Supplement: S2 File — (ZIP) [file pone.0299471.s002.zip › 0257.png]

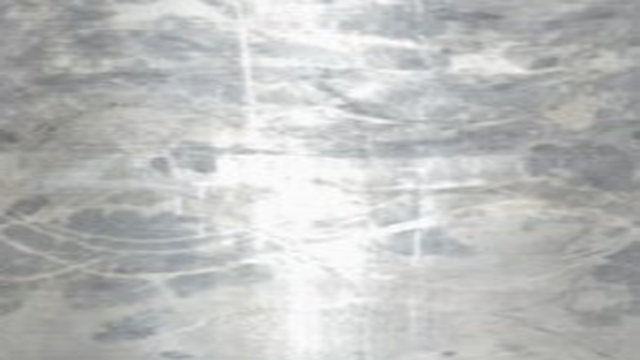

Supplement: S2 File — (ZIP) [file pone.0299471.s002.zip › 0258.png]

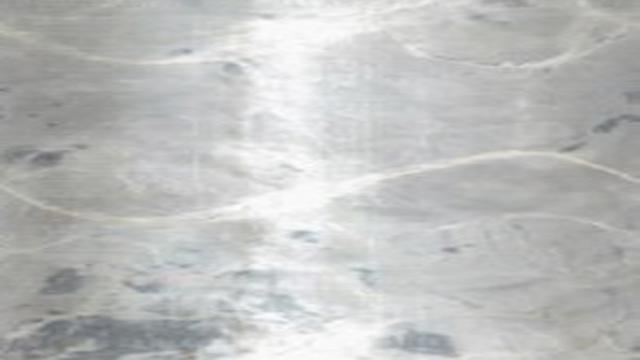

Supplement: S2 File — (ZIP) [file pone.0299471.s002.zip › 0259.jpg]

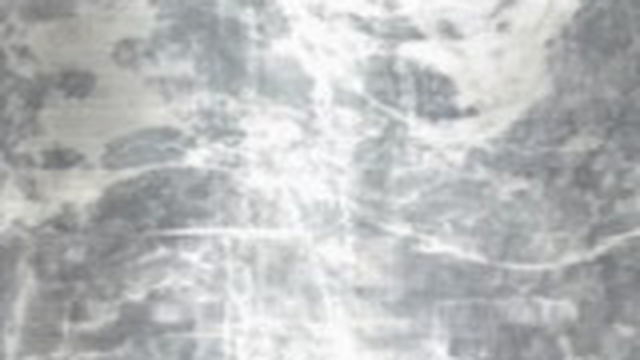

Supplement: S2 File — (ZIP) [file pone.0299471.s002.zip › 0260.png]

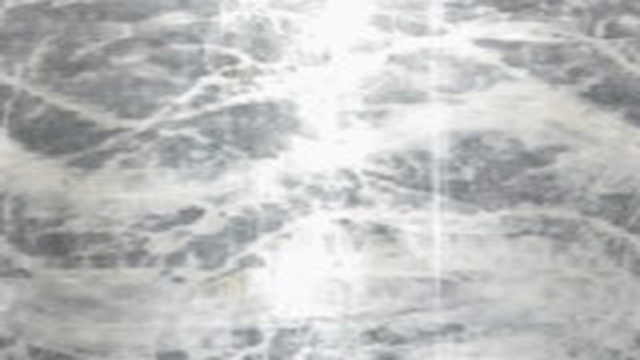

Supplement: S2 File — (ZIP) [file pone.0299471.s002.zip › 0261.png]

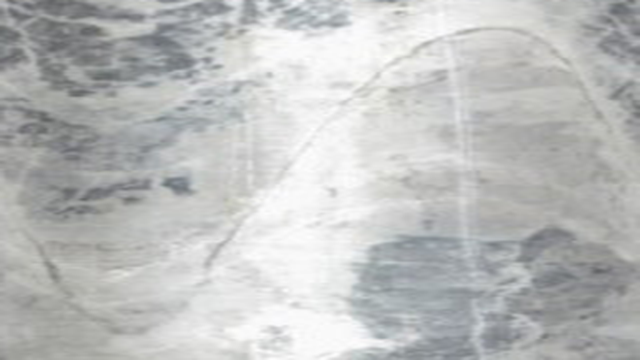

Supplement: S2 File — (ZIP) [file pone.0299471.s002.zip › 0262.png]

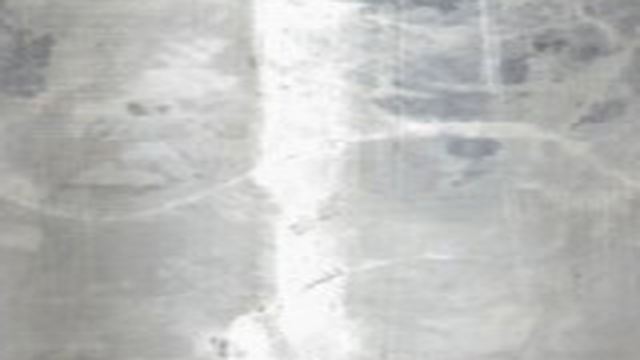

Supplement: S2 File — (ZIP) [file pone.0299471.s002.zip › 0263.png]

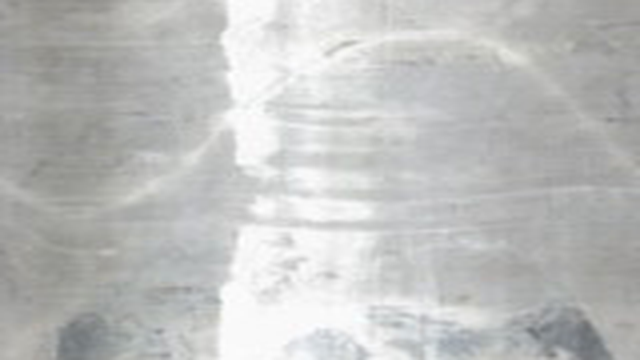

Supplement: S2 File — (ZIP) [file pone.0299471.s002.zip › 0264.png]

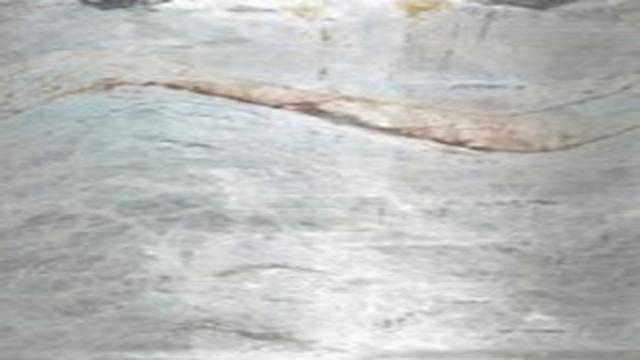

Supplement: S2 File — (ZIP) [file pone.0299471.s002.zip › 0265.jpg]

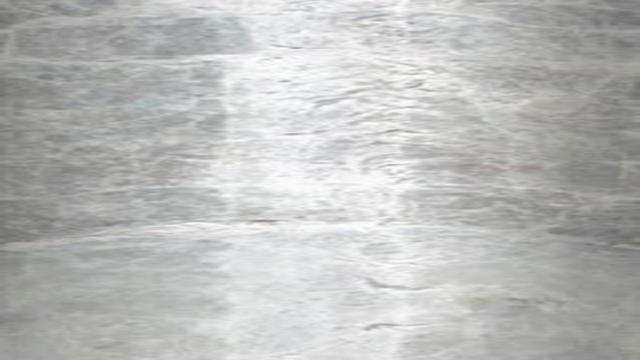

Supplement: S2 File — (ZIP) [file pone.0299471.s002.zip › 0266.jpg]

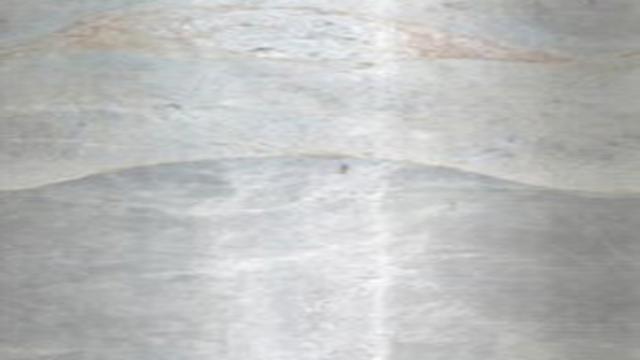

Supplement: S2 File — (ZIP) [file pone.0299471.s002.zip › 0267.jpg]

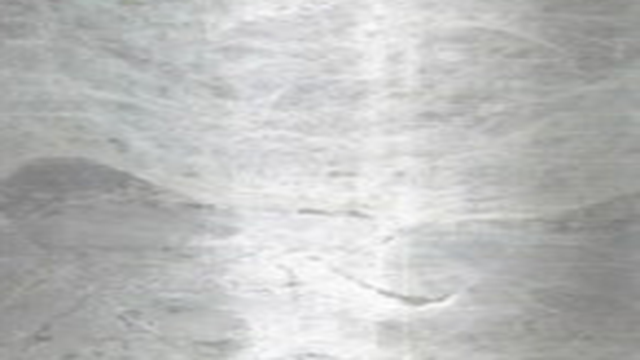

Supplement: S2 File — (ZIP) [file pone.0299471.s002.zip › 0268.png]

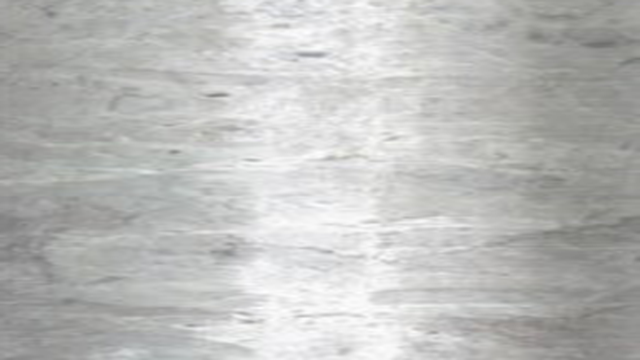

Supplement: S2 File — (ZIP) [file pone.0299471.s002.zip › 0269.png]

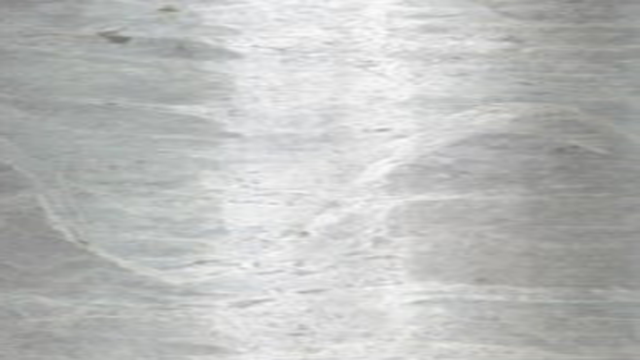

Supplement: S2 File — (ZIP) [file pone.0299471.s002.zip › 0270.png]

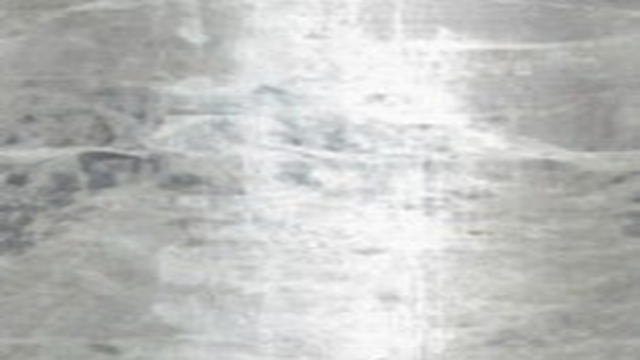

Supplement: S2 File — (ZIP) [file pone.0299471.s002.zip › 0271.png]

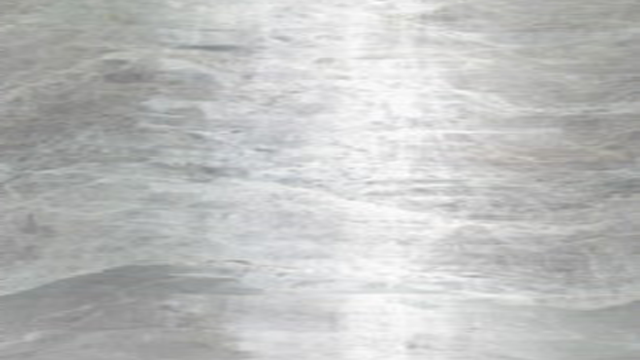

Supplement: S2 File — (ZIP) [file pone.0299471.s002.zip › 0272.png]

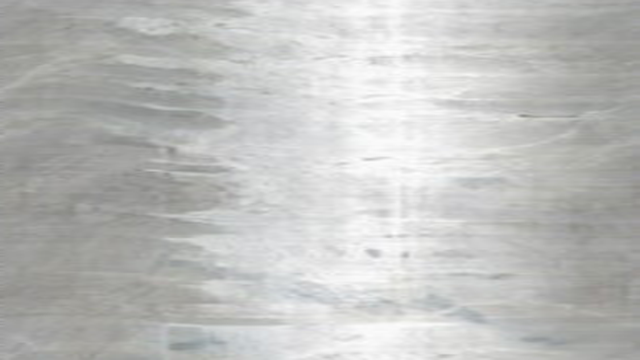

Supplement: S2 File — (ZIP) [file pone.0299471.s002.zip › 0273.png]

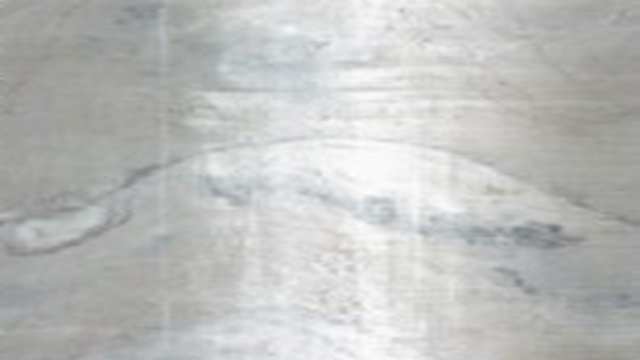

Supplement: S2 File — (ZIP) [file pone.0299471.s002.zip › 0274.png]

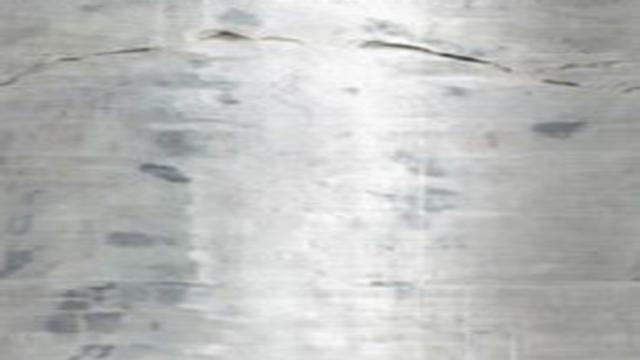

Supplement: S2 File — (ZIP) [file pone.0299471.s002.zip › 0275.jpg]

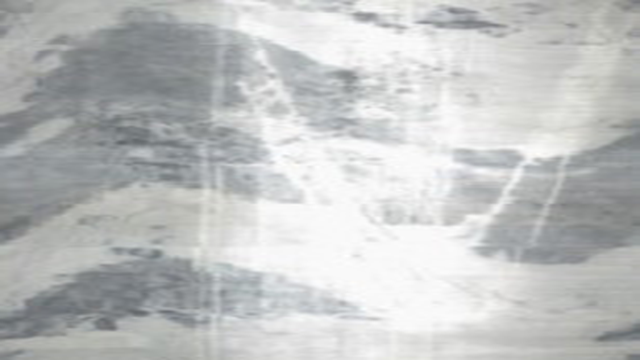

Supplement: S2 File — (ZIP) [file pone.0299471.s002.zip › 0276.png]

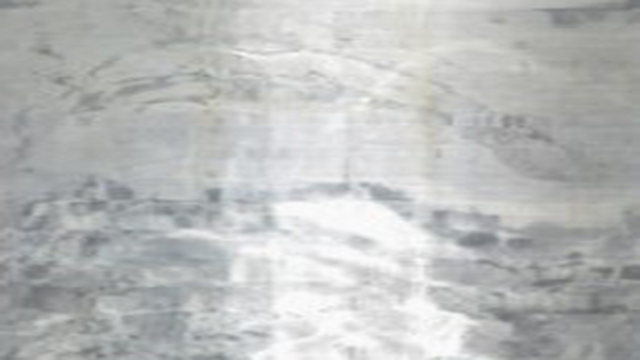

Supplement: S2 File — (ZIP) [file pone.0299471.s002.zip › 0277.png]

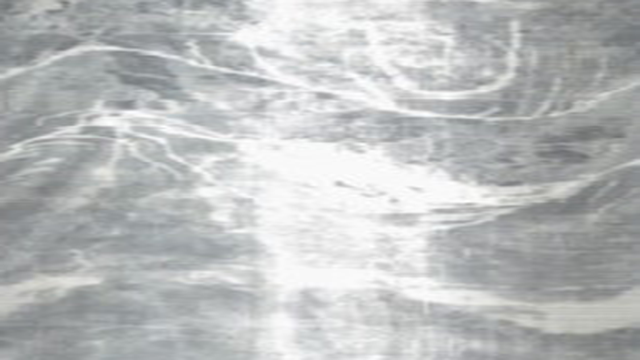

Supplement: S2 File — (ZIP) [file pone.0299471.s002.zip › 0278.png]

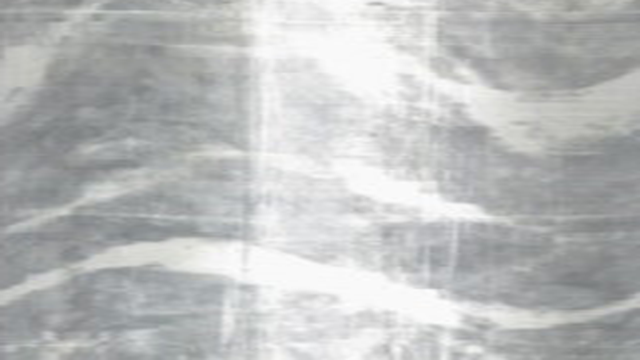

Supplement: S2 File — (ZIP) [file pone.0299471.s002.zip › 0279.png]

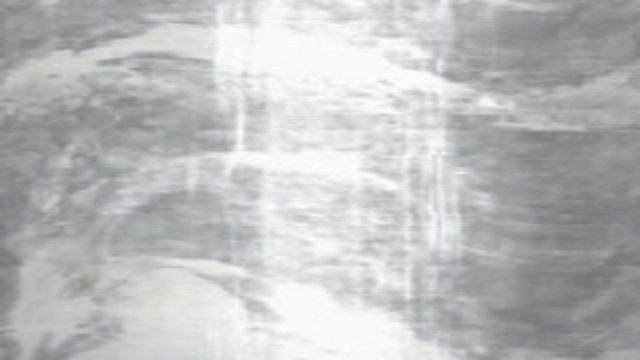

Supplement: S2 File — (ZIP) [file pone.0299471.s002.zip › 0280.png]

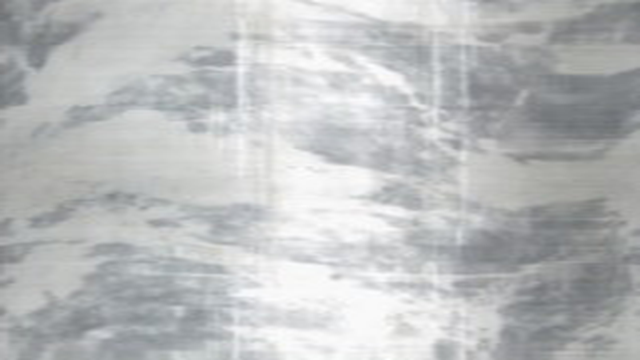

Supplement: S2 File — (ZIP) [file pone.0299471.s002.zip › 0281.png]

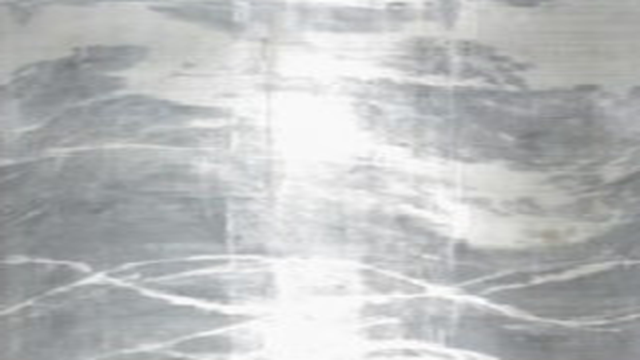

Supplement: S2 File — (ZIP) [file pone.0299471.s002.zip › 0282.png]

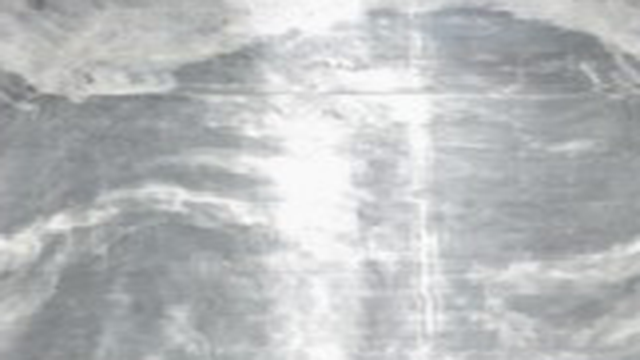

Supplement: S2 File — (ZIP) [file pone.0299471.s002.zip › 0283.png]

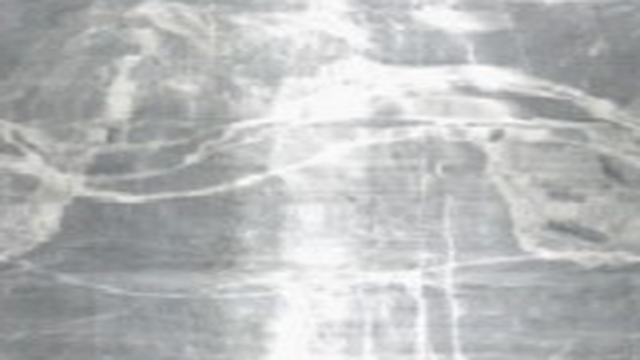

Supplement: S2 File — (ZIP) [file pone.0299471.s002.zip › 0284.png]

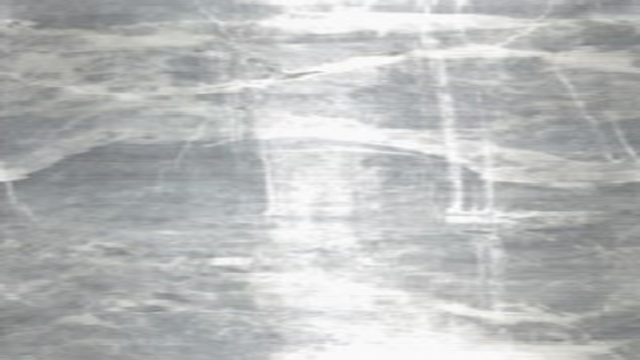

Supplement: S2 File — (ZIP) [file pone.0299471.s002.zip › 0285.png]

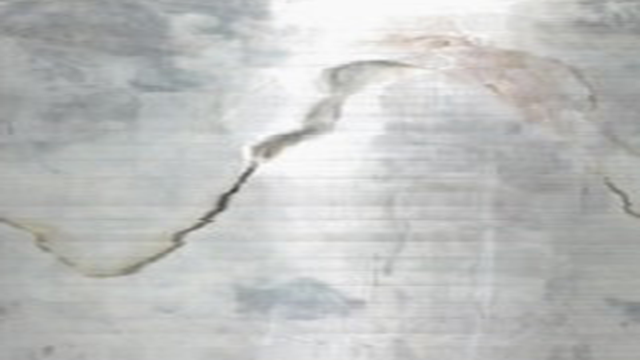

Supplement: S2 File — (ZIP) [file pone.0299471.s002.zip › 0286.png]

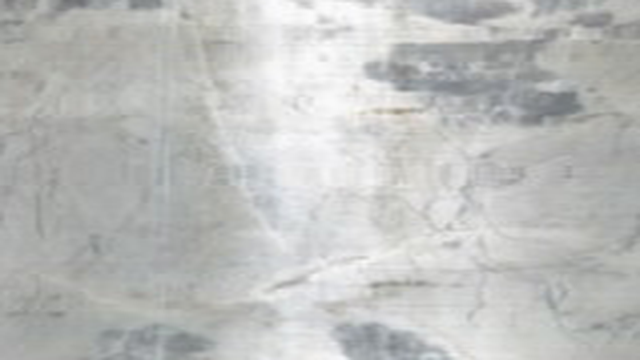

Supplement: S2 File — (ZIP) [file pone.0299471.s002.zip › 0287.png]

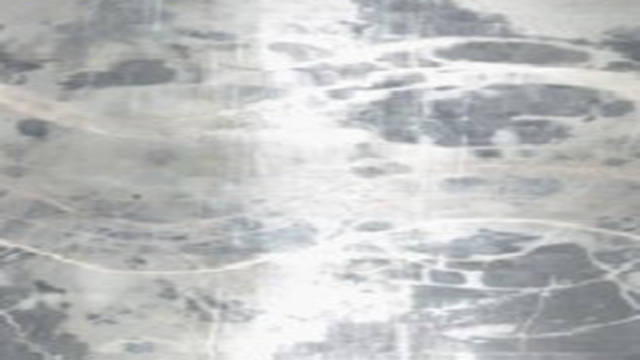

Supplement: S2 File — (ZIP) [file pone.0299471.s002.zip › 0288.png]

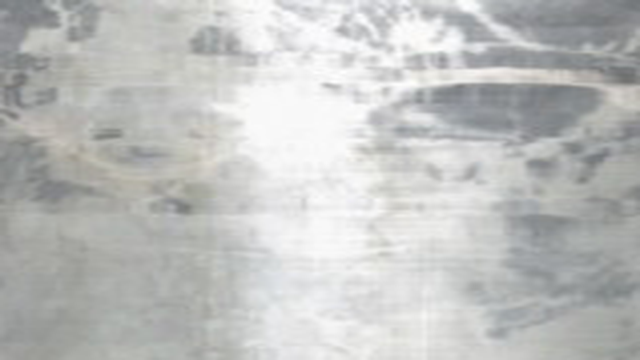

Supplement: S2 File — (ZIP) [file pone.0299471.s002.zip › 0289.png]

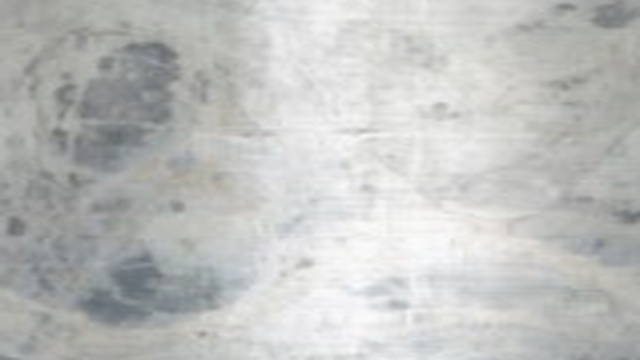

Supplement: S2 File — (ZIP) [file pone.0299471.s002.zip › 0290.png]

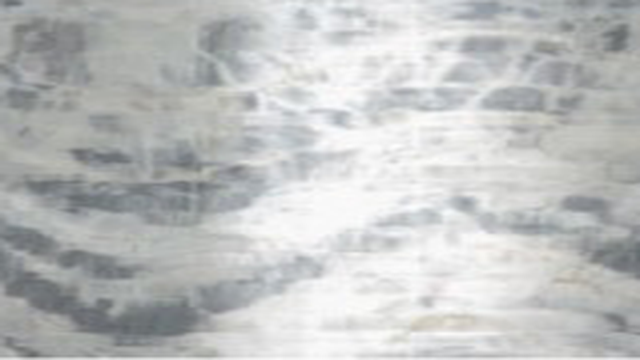

Supplement: S2 File — (ZIP) [file pone.0299471.s002.zip › 0291.png]

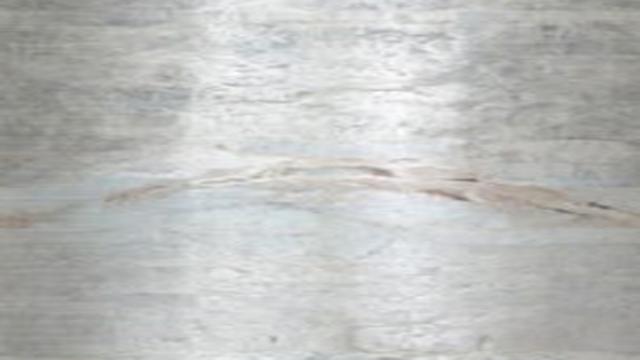

Supplement: S2 File — (ZIP) [file pone.0299471.s002.zip › 0292.jpg]

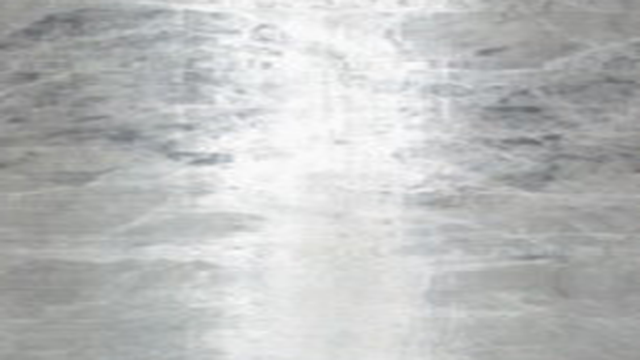

Supplement: S2 File — (ZIP) [file pone.0299471.s002.zip › 0293.png]

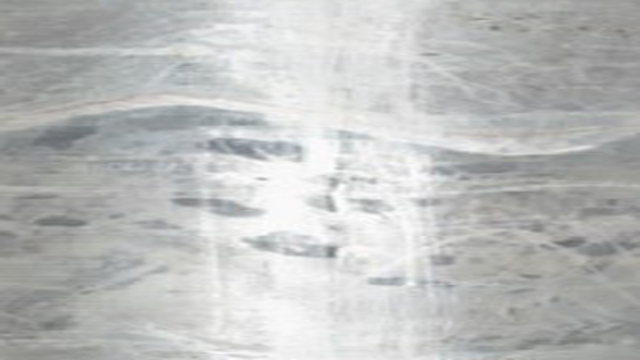

Supplement: S2 File — (ZIP) [file pone.0299471.s002.zip › 0294.png]

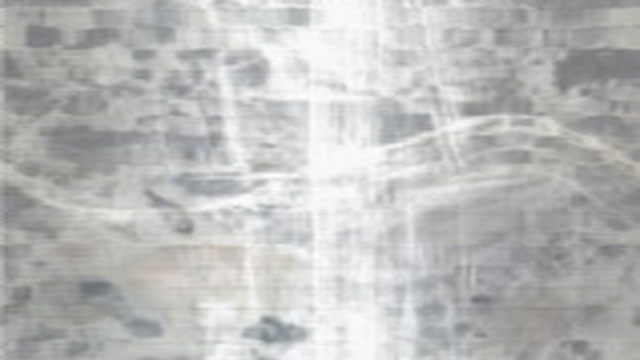

Supplement: S2 File — (ZIP) [file pone.0299471.s002.zip › 0295.png]

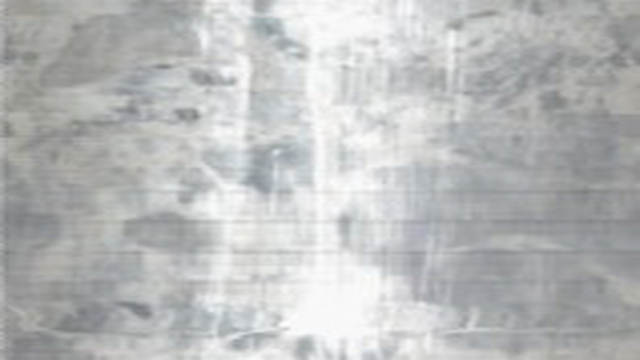

Supplement: S2 File — (ZIP) [file pone.0299471.s002.zip › 0296.png]

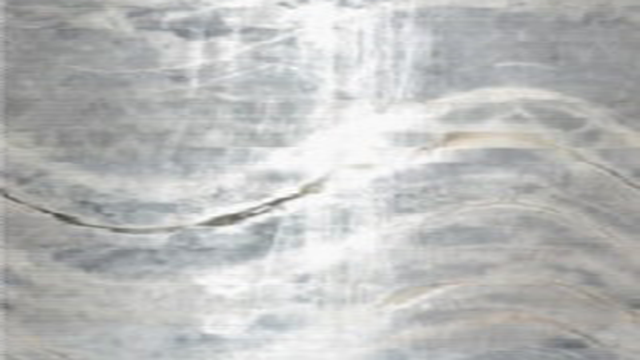

Supplement: S2 File — (ZIP) [file pone.0299471.s002.zip › 0297.png]

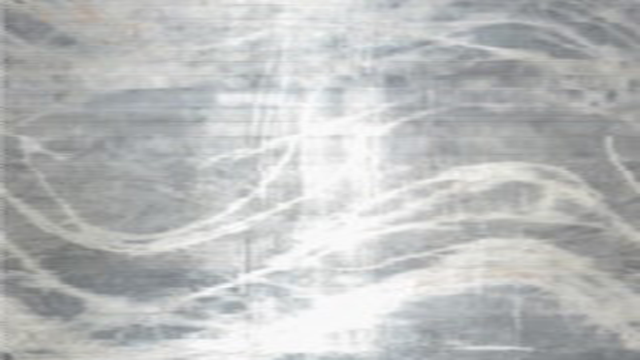

Supplement: S2 File — (ZIP) [file pone.0299471.s002.zip › 0298.png]

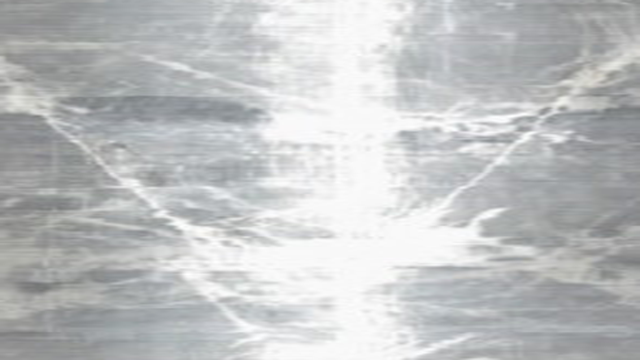

Supplement: S2 File — (ZIP) [file pone.0299471.s002.zip › 0299.png]
